# Correction: Synaptic proteins promote calcium-triggered fast transition from point contact to full fusion

**DOI:** 10.7554/eLife.12289

**Published:** 2015-10-21

**Authors:** Jiajie Diao, Patricia Grob, Daniel J Cipriano, Minjoung Kyoung, Yunxiang Zhang, Sachi Shah, Amie Nguyen, Mark Padolina, Ankita Srivastava, Marija Vrljic, Ankita Shah, Eva Nogales, Steven Chu, Axel T Brunger

Diao J, Grob P, Cipriano DJ, Kyoung M, Zhang Y, Shah S, Nguyen A, Padolina M, Srivastava A, Vrljic M, Shah A, Nogales E, Chu S, Brunger AT. 2012. Synaptic proteins promote calcium-triggered fast transition from point contact to full fusion. *eLife* **1**:e00109. doi: 10.7554/eLife.00109

Published 13 December 2012

In the published article, a number of small bars were missing from the lower panel of Figure 6A in the time range 10 to 20 sec. The fit to the data and the conclusions are not affected by the inadvertent omission of these small bars.

Original panel:

**Figure 1:**
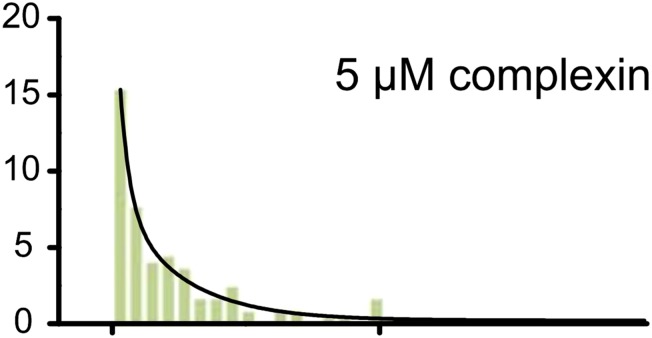

Corrected panel:

**Figure 2:**
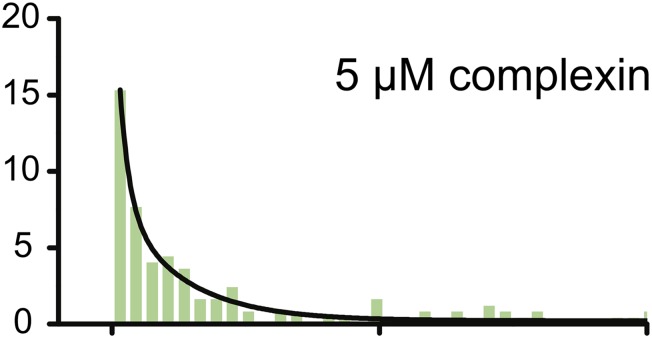

The article has now been corrected.

