# Supplementary material for: Synaptic proteins promote calcium-triggered fast transition from point contact to full fusion
Source: eLife. 2012 Dec 13;1:e00109. doi: 10.7554/eLife.00109 (PMC3514886; doi:10.7554/eLife.00109)
Supplement: Figure 2—source data 1. — This zip archive contains all cryo-EM images used for the quantitative analyses shown in Fig. 2. The folder named “No_Ca++” contains the images before Ca++ addition (individual files are named P3_1_**. tif or jpg), and folder named “With_Ca++” contains the images ∼35s after Ca++ addition (individual files are named P3_3_**.tif or jpg). Images were collected in low dose conditions at 200 kV acceleration voltage on a CM200 FEG electron microscope (FEI) with a 2k × 2k Gatan UltraScan 1000 camera, at 50,000× magnification and 1.5 mm underfocus. The full resolution data were exported as 16 bit “tif” files (2048 × 2048 pixels, scale 0.2 nm/pixel at specimen (the corresponding files have the extension “tif”). Note that these files cannot not be viewed with a standard picture viewer, but must be viewed with a program, such as “ImageJ”. To facilitate easier viewing, the original images were converted to smaller (1024×1024, 0.4 nm/pixel), contrast adjusted jpeg images (8 bits) for easy and immediate visualization with commonly used picture viewers (the corresponding files have the extension “jpg”). DOI: http://dx.doi.org/10.7554/eLife.00109.005 [file elife00109s001.zip › elife00109s001/Cryo_EM.docx]

**Source files for Cryo-EM data (related to Figure 2)**

This zip archive contains all cryo-EM images used for the quantitative analyses shown in Fig. 2. The folder named "No_Ca++" contains the images before Ca++ addition (individual files are named P3_1_**. tif or jpg), and folder named "With_Ca++" contains the images ~35s after Ca++ addition (individual files are named P3_3_**.tif or jpg).

Images were collected in low dose conditions at 200 kV acceleration voltage on a CM200 FEG electron microscope (FEI) with a 2k x 2k Gatan UltraScan 1000 camera, at 50,000x magnification and 1.5 μm underfocus.

The full resolution data were exported as 16 bit "tif" files (2048 x 2048 pixels, scale 0.2 nm/pixel at specimen (the corresponding files have the extension "tif"). Note that these files cannot not be viewed with a standard picture viewer, but must be viewed with a program, such as "ImageJ". To facilitate easier viewing, the original images were converted to smaller (1024x1024, 0.4 nm/pixel), contrast adjusted jpeg images (8 bits) for easy and immediate visualization with commonly used picture viewers (the corresponding files have the extension "jpg").
