# Supplementary figures and images for: Synaptic proteins promote calcium-triggered fast transition from point contact to full fusion
Source: eLife. 2012 Dec 13;1:e00109. doi: 10.7554/eLife.00109 (PMC3514886; doi:10.7554/eLife.00109)

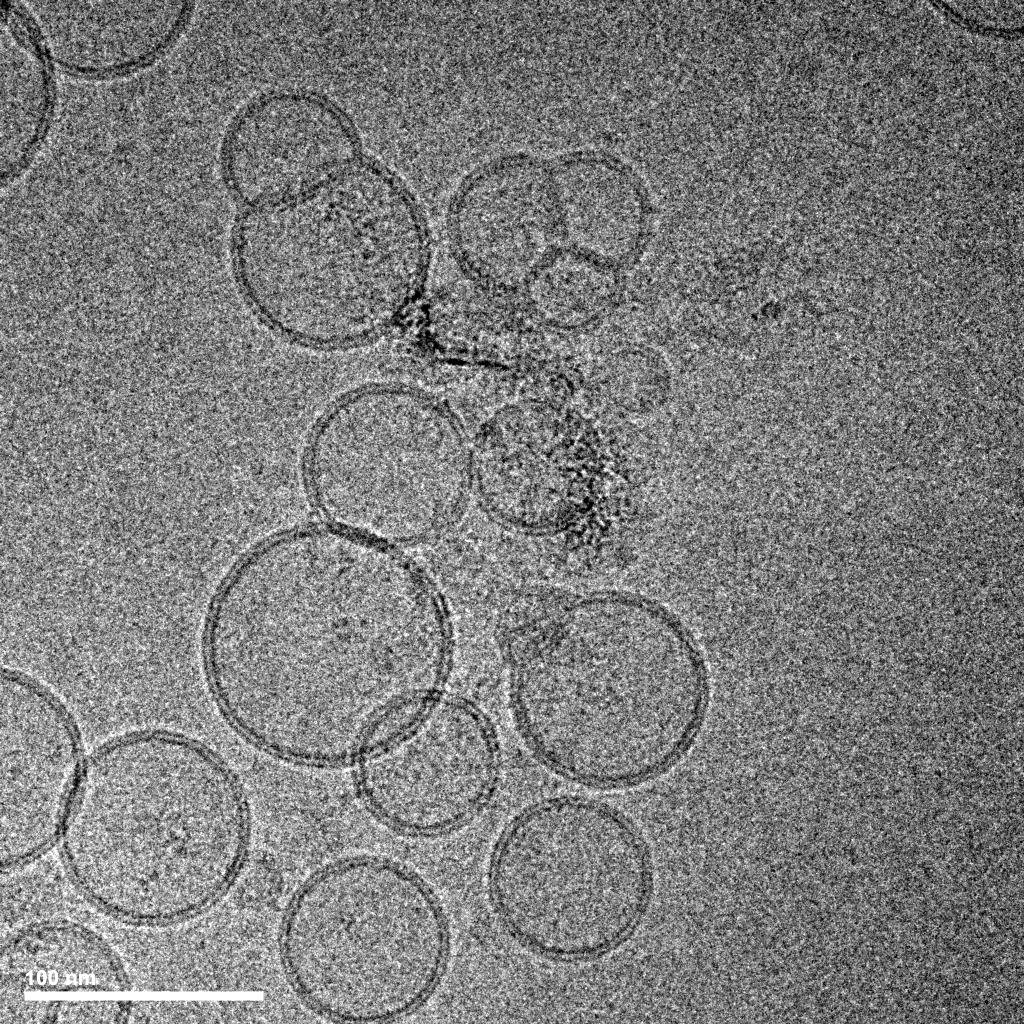

Supplement: Figure 2—source data 1. — This zip archive contains all cryo-EM images used for the quantitative analyses shown in Fig. 2. The folder named “No_Ca++” contains the images before Ca++ addition (individual files are named P3_1_**. tif or jpg), and folder named “With_Ca++” contains the images ∼35s after Ca++ addition (individual files are named P3_3_**.tif or jpg). Images were collected in low dose conditions at 200 kV acceleration voltage on a CM200 FEG electron microscope (FEI) with a 2k × 2k Gatan UltraScan 1000 camera, at 50,000× magnification and 1.5 mm underfocus. The full resolution data were exported as 16 bit “tif” files (2048 × 2048 pixels, scale 0.2 nm/pixel at specimen (the corresponding files have the extension “tif”). Note that these files cannot not be viewed with a standard picture viewer, but must be viewed with a program, such as “ImageJ”. To facilitate easier viewing, the original images were converted to smaller (1024×1024, 0.4 nm/pixel), contrast adjusted jpeg images (8 bits) for easy and immediate visualization with commonly used picture viewers (the corresponding files have the extension “jpg”). DOI: http://dx.doi.org/10.7554/eLife.00109.005 [file elife00109s001.zip › elife00109s001/NO_Ca++/P3_1_02.jpg]

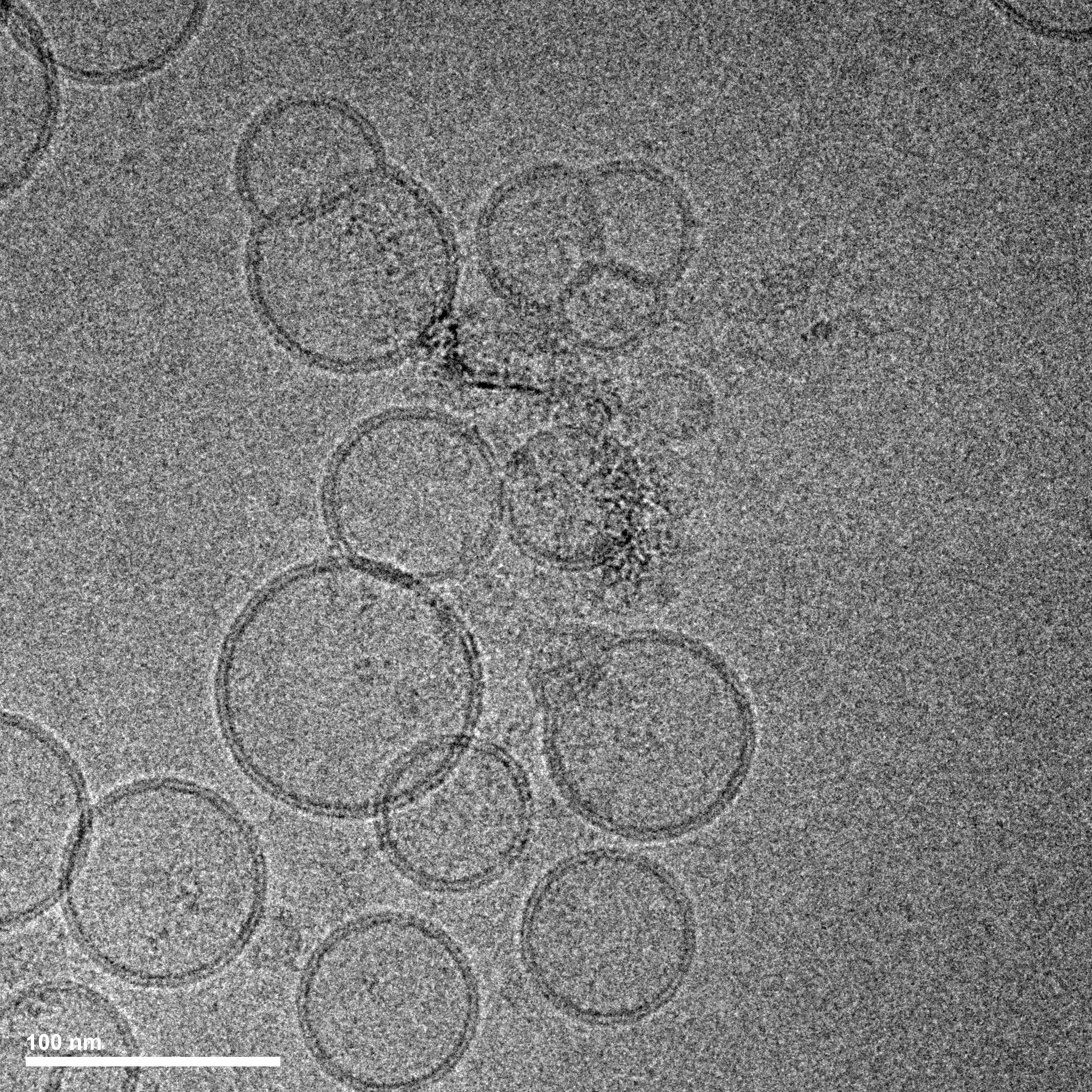

Supplement: Figure 2—source data 1. — This zip archive contains all cryo-EM images used for the quantitative analyses shown in Fig. 2. The folder named “No_Ca++” contains the images before Ca++ addition (individual files are named P3_1_**. tif or jpg), and folder named “With_Ca++” contains the images ∼35s after Ca++ addition (individual files are named P3_3_**.tif or jpg). Images were collected in low dose conditions at 200 kV acceleration voltage on a CM200 FEG electron microscope (FEI) with a 2k × 2k Gatan UltraScan 1000 camera, at 50,000× magnification and 1.5 mm underfocus. The full resolution data were exported as 16 bit “tif” files (2048 × 2048 pixels, scale 0.2 nm/pixel at specimen (the corresponding files have the extension “tif”). Note that these files cannot not be viewed with a standard picture viewer, but must be viewed with a program, such as “ImageJ”. To facilitate easier viewing, the original images were converted to smaller (1024×1024, 0.4 nm/pixel), contrast adjusted jpeg images (8 bits) for easy and immediate visualization with commonly used picture viewers (the corresponding files have the extension “jpg”). DOI: http://dx.doi.org/10.7554/eLife.00109.005 [file elife00109s001.zip › elife00109s001/NO_Ca++/P3_1_02.tif]

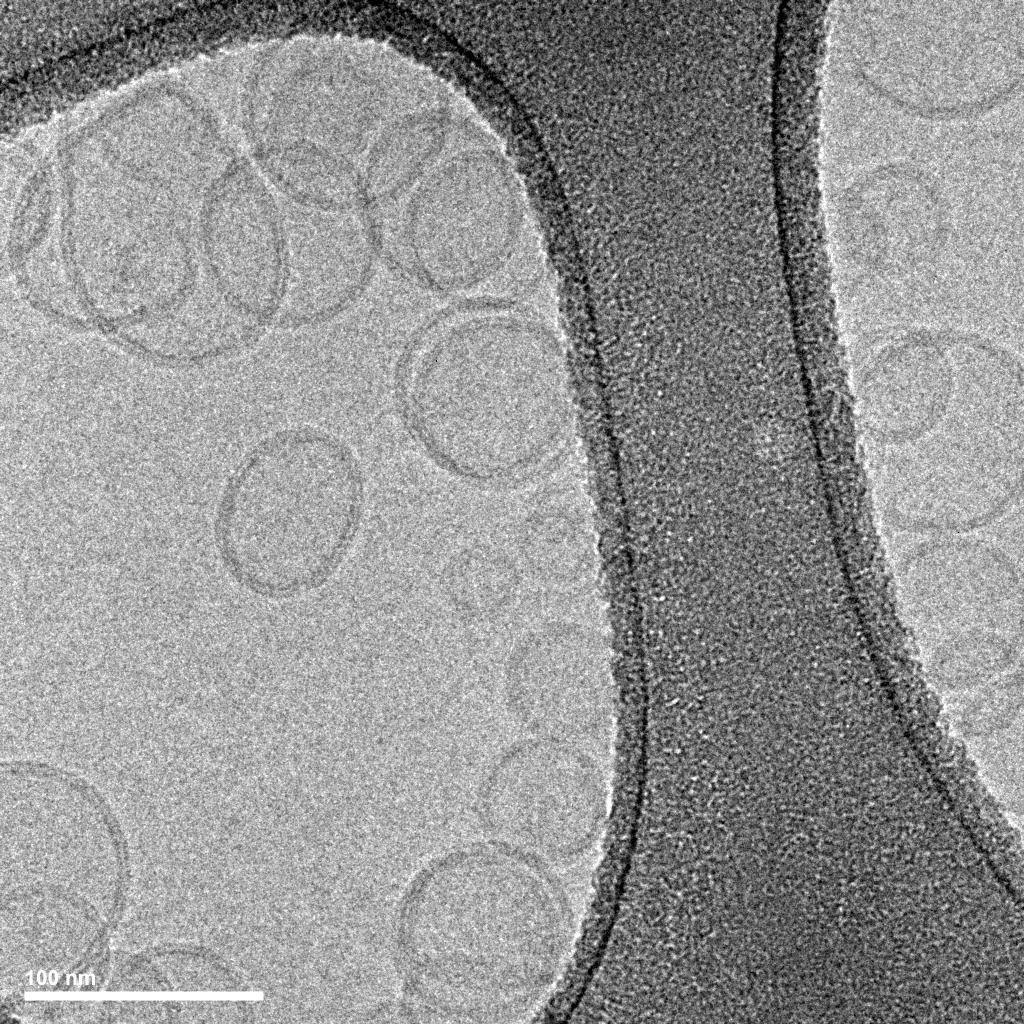

Supplement: Figure 2—source data 1. — This zip archive contains all cryo-EM images used for the quantitative analyses shown in Fig. 2. The folder named “No_Ca++” contains the images before Ca++ addition (individual files are named P3_1_**. tif or jpg), and folder named “With_Ca++” contains the images ∼35s after Ca++ addition (individual files are named P3_3_**.tif or jpg). Images were collected in low dose conditions at 200 kV acceleration voltage on a CM200 FEG electron microscope (FEI) with a 2k × 2k Gatan UltraScan 1000 camera, at 50,000× magnification and 1.5 mm underfocus. The full resolution data were exported as 16 bit “tif” files (2048 × 2048 pixels, scale 0.2 nm/pixel at specimen (the corresponding files have the extension “tif”). Note that these files cannot not be viewed with a standard picture viewer, but must be viewed with a program, such as “ImageJ”. To facilitate easier viewing, the original images were converted to smaller (1024×1024, 0.4 nm/pixel), contrast adjusted jpeg images (8 bits) for easy and immediate visualization with commonly used picture viewers (the corresponding files have the extension “jpg”). DOI: http://dx.doi.org/10.7554/eLife.00109.005 [file elife00109s001.zip › elife00109s001/NO_Ca++/P3_1_03.jpg]

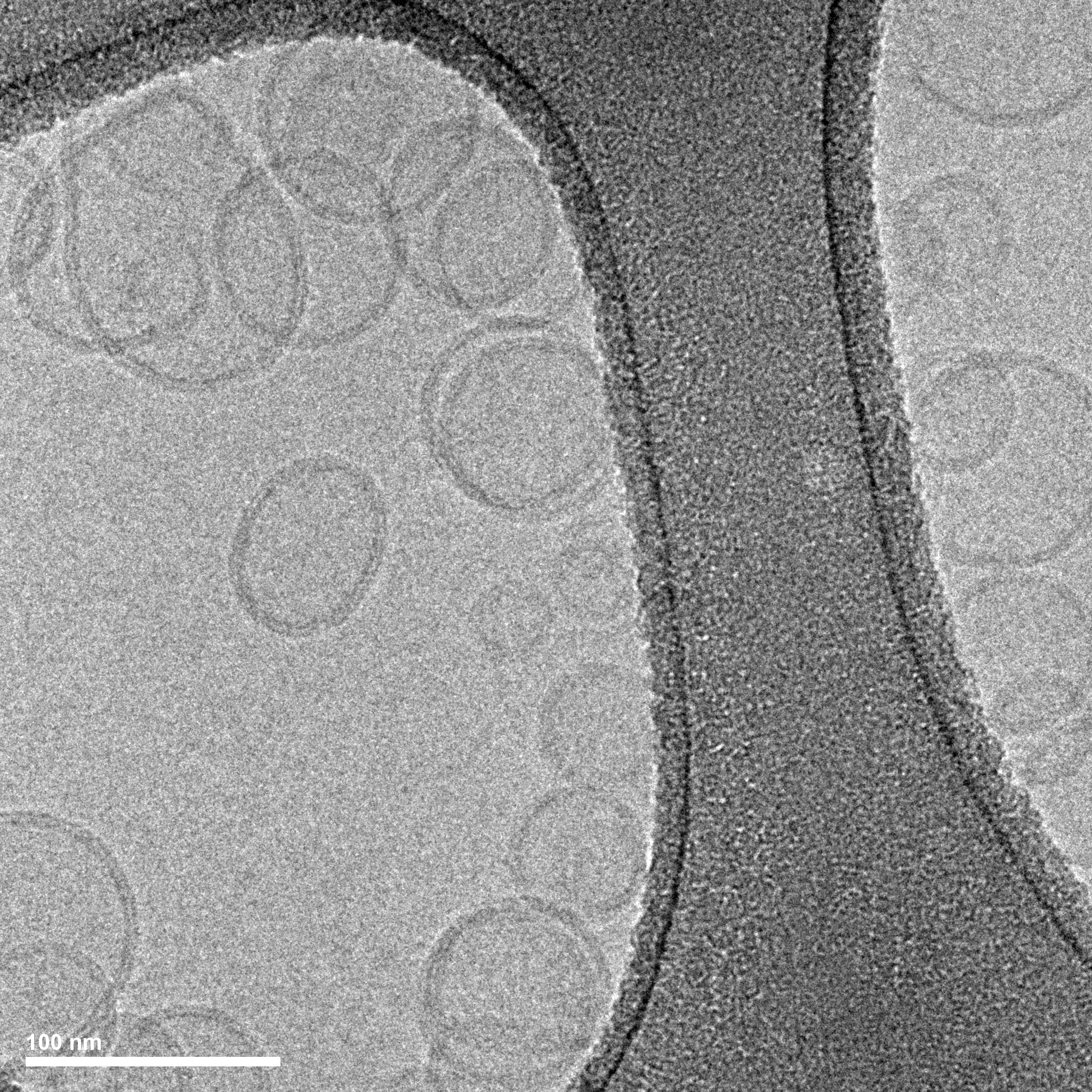

Supplement: Figure 2—source data 1. — This zip archive contains all cryo-EM images used for the quantitative analyses shown in Fig. 2. The folder named “No_Ca++” contains the images before Ca++ addition (individual files are named P3_1_**. tif or jpg), and folder named “With_Ca++” contains the images ∼35s after Ca++ addition (individual files are named P3_3_**.tif or jpg). Images were collected in low dose conditions at 200 kV acceleration voltage on a CM200 FEG electron microscope (FEI) with a 2k × 2k Gatan UltraScan 1000 camera, at 50,000× magnification and 1.5 mm underfocus. The full resolution data were exported as 16 bit “tif” files (2048 × 2048 pixels, scale 0.2 nm/pixel at specimen (the corresponding files have the extension “tif”). Note that these files cannot not be viewed with a standard picture viewer, but must be viewed with a program, such as “ImageJ”. To facilitate easier viewing, the original images were converted to smaller (1024×1024, 0.4 nm/pixel), contrast adjusted jpeg images (8 bits) for easy and immediate visualization with commonly used picture viewers (the corresponding files have the extension “jpg”). DOI: http://dx.doi.org/10.7554/eLife.00109.005 [file elife00109s001.zip › elife00109s001/NO_Ca++/P3_1_03.tif]

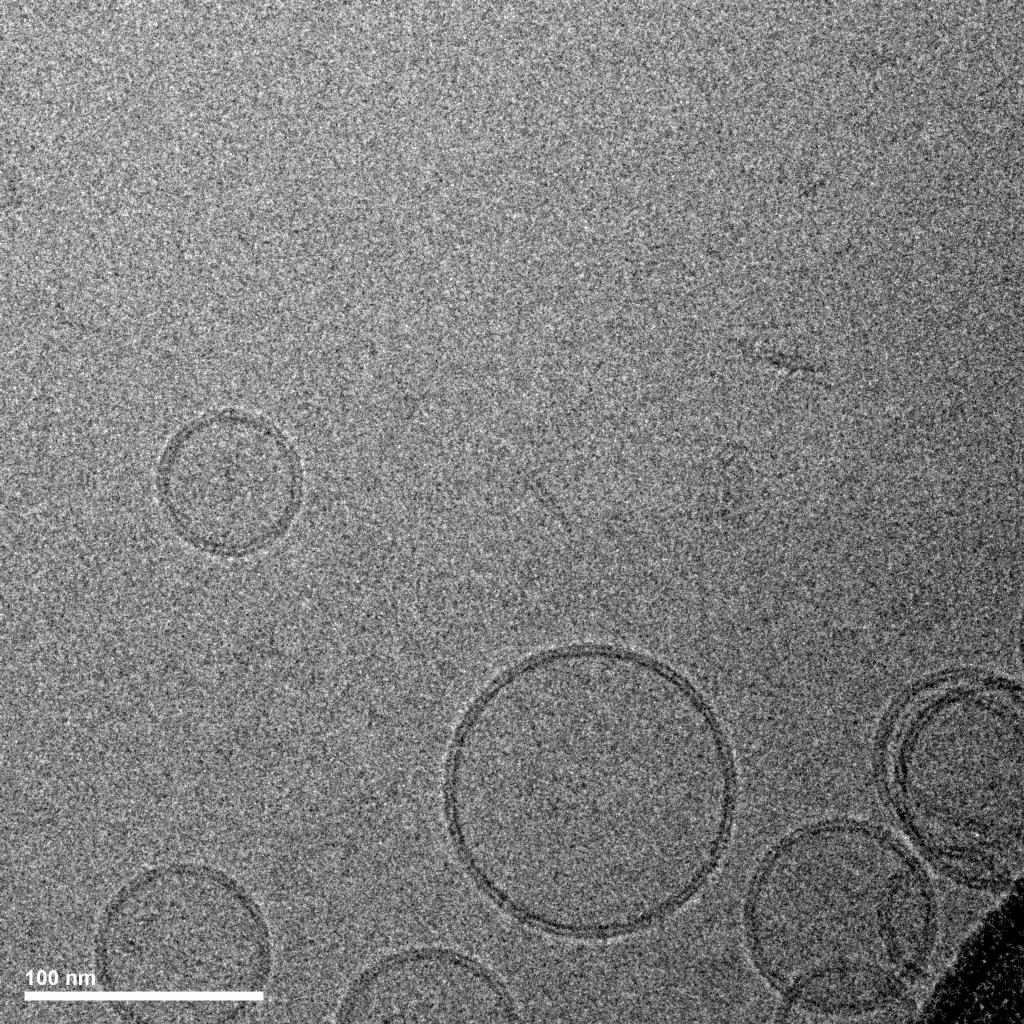

Supplement: Figure 2—source data 1. — This zip archive contains all cryo-EM images used for the quantitative analyses shown in Fig. 2. The folder named “No_Ca++” contains the images before Ca++ addition (individual files are named P3_1_**. tif or jpg), and folder named “With_Ca++” contains the images ∼35s after Ca++ addition (individual files are named P3_3_**.tif or jpg). Images were collected in low dose conditions at 200 kV acceleration voltage on a CM200 FEG electron microscope (FEI) with a 2k × 2k Gatan UltraScan 1000 camera, at 50,000× magnification and 1.5 mm underfocus. The full resolution data were exported as 16 bit “tif” files (2048 × 2048 pixels, scale 0.2 nm/pixel at specimen (the corresponding files have the extension “tif”). Note that these files cannot not be viewed with a standard picture viewer, but must be viewed with a program, such as “ImageJ”. To facilitate easier viewing, the original images were converted to smaller (1024×1024, 0.4 nm/pixel), contrast adjusted jpeg images (8 bits) for easy and immediate visualization with commonly used picture viewers (the corresponding files have the extension “jpg”). DOI: http://dx.doi.org/10.7554/eLife.00109.005 [file elife00109s001.zip › elife00109s001/NO_Ca++/P3_1_05.jpg]

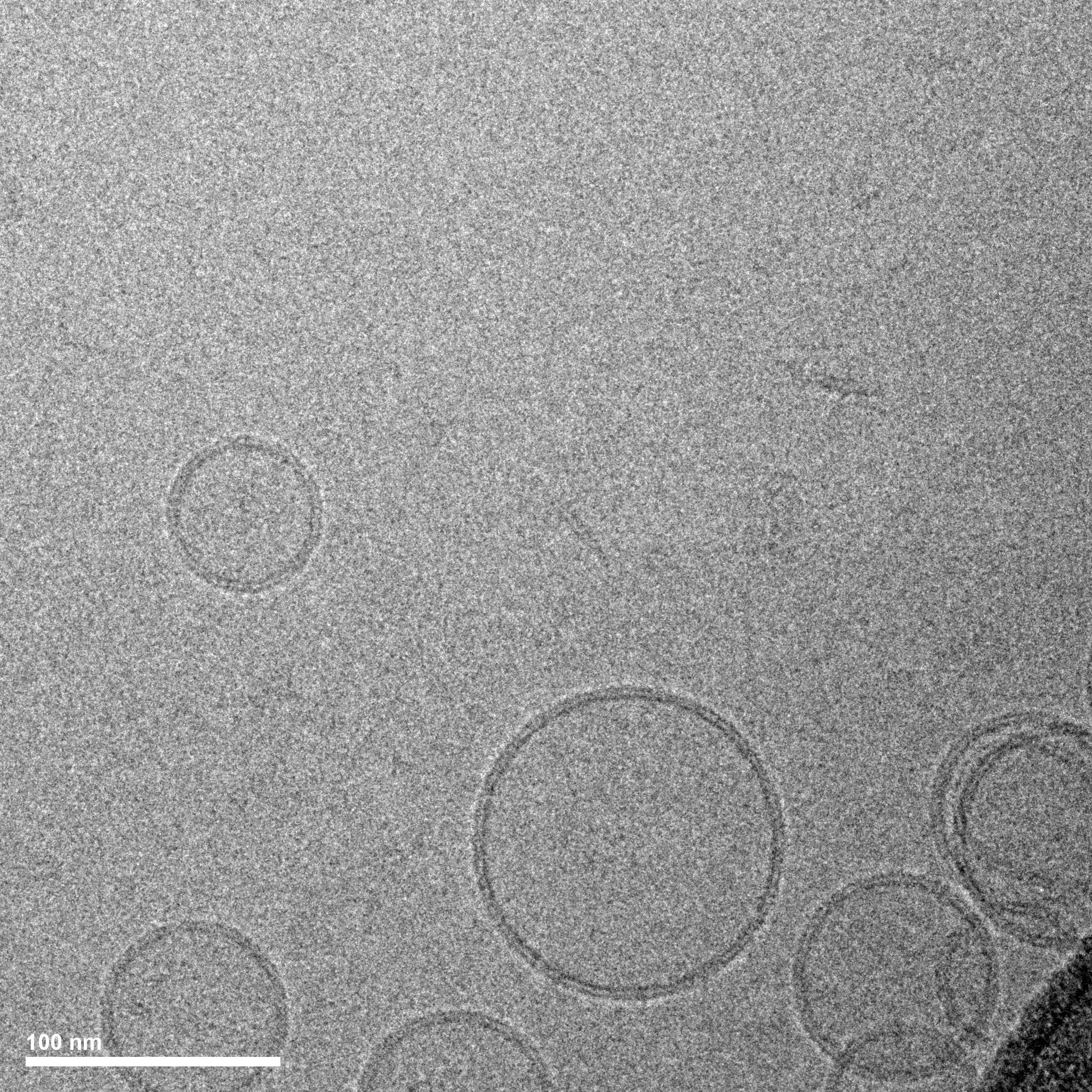

Supplement: Figure 2—source data 1. — This zip archive contains all cryo-EM images used for the quantitative analyses shown in Fig. 2. The folder named “No_Ca++” contains the images before Ca++ addition (individual files are named P3_1_**. tif or jpg), and folder named “With_Ca++” contains the images ∼35s after Ca++ addition (individual files are named P3_3_**.tif or jpg). Images were collected in low dose conditions at 200 kV acceleration voltage on a CM200 FEG electron microscope (FEI) with a 2k × 2k Gatan UltraScan 1000 camera, at 50,000× magnification and 1.5 mm underfocus. The full resolution data were exported as 16 bit “tif” files (2048 × 2048 pixels, scale 0.2 nm/pixel at specimen (the corresponding files have the extension “tif”). Note that these files cannot not be viewed with a standard picture viewer, but must be viewed with a program, such as “ImageJ”. To facilitate easier viewing, the original images were converted to smaller (1024×1024, 0.4 nm/pixel), contrast adjusted jpeg images (8 bits) for easy and immediate visualization with commonly used picture viewers (the corresponding files have the extension “jpg”). DOI: http://dx.doi.org/10.7554/eLife.00109.005 [file elife00109s001.zip › elife00109s001/NO_Ca++/P3_1_05.tif]

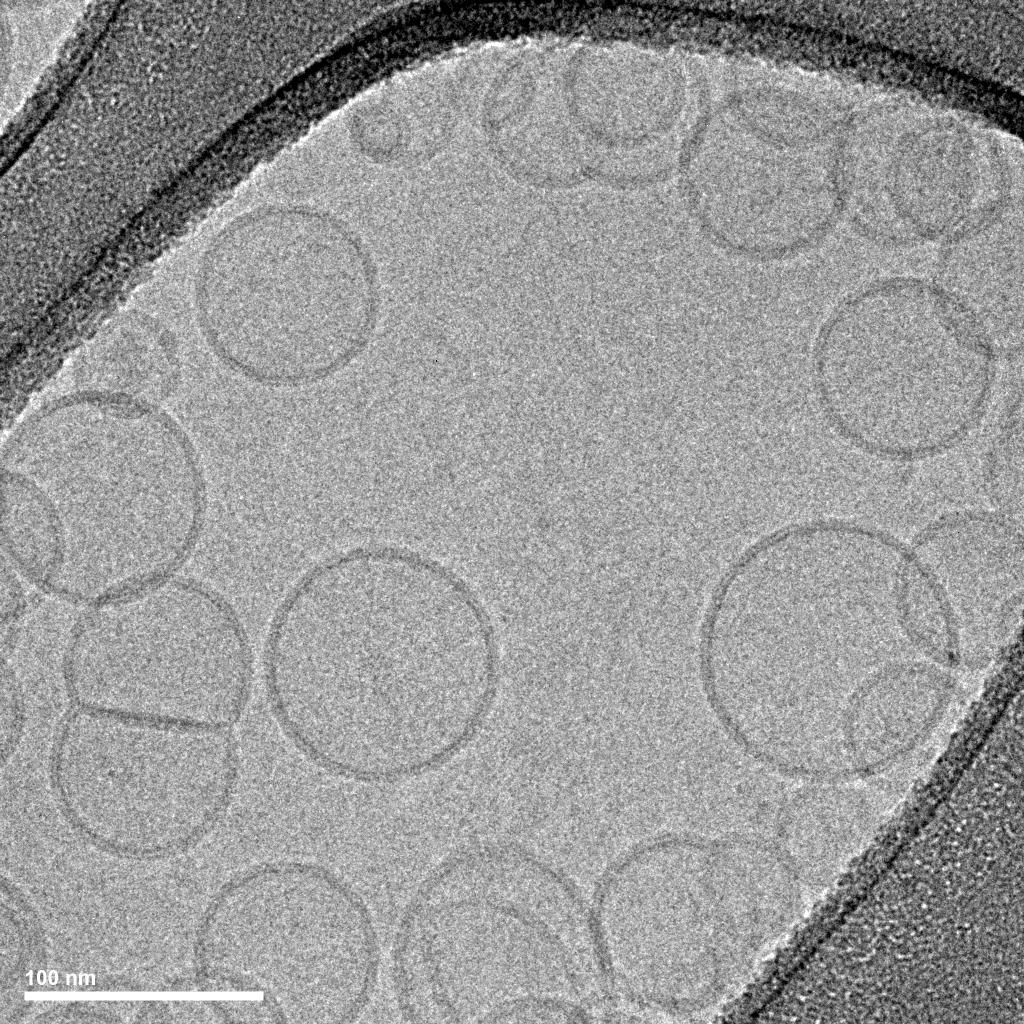

Supplement: Figure 2—source data 1. — This zip archive contains all cryo-EM images used for the quantitative analyses shown in Fig. 2. The folder named “No_Ca++” contains the images before Ca++ addition (individual files are named P3_1_**. tif or jpg), and folder named “With_Ca++” contains the images ∼35s after Ca++ addition (individual files are named P3_3_**.tif or jpg). Images were collected in low dose conditions at 200 kV acceleration voltage on a CM200 FEG electron microscope (FEI) with a 2k × 2k Gatan UltraScan 1000 camera, at 50,000× magnification and 1.5 mm underfocus. The full resolution data were exported as 16 bit “tif” files (2048 × 2048 pixels, scale 0.2 nm/pixel at specimen (the corresponding files have the extension “tif”). Note that these files cannot not be viewed with a standard picture viewer, but must be viewed with a program, such as “ImageJ”. To facilitate easier viewing, the original images were converted to smaller (1024×1024, 0.4 nm/pixel), contrast adjusted jpeg images (8 bits) for easy and immediate visualization with commonly used picture viewers (the corresponding files have the extension “jpg”). DOI: http://dx.doi.org/10.7554/eLife.00109.005 [file elife00109s001.zip › elife00109s001/NO_Ca++/P3_1_06.jpg]

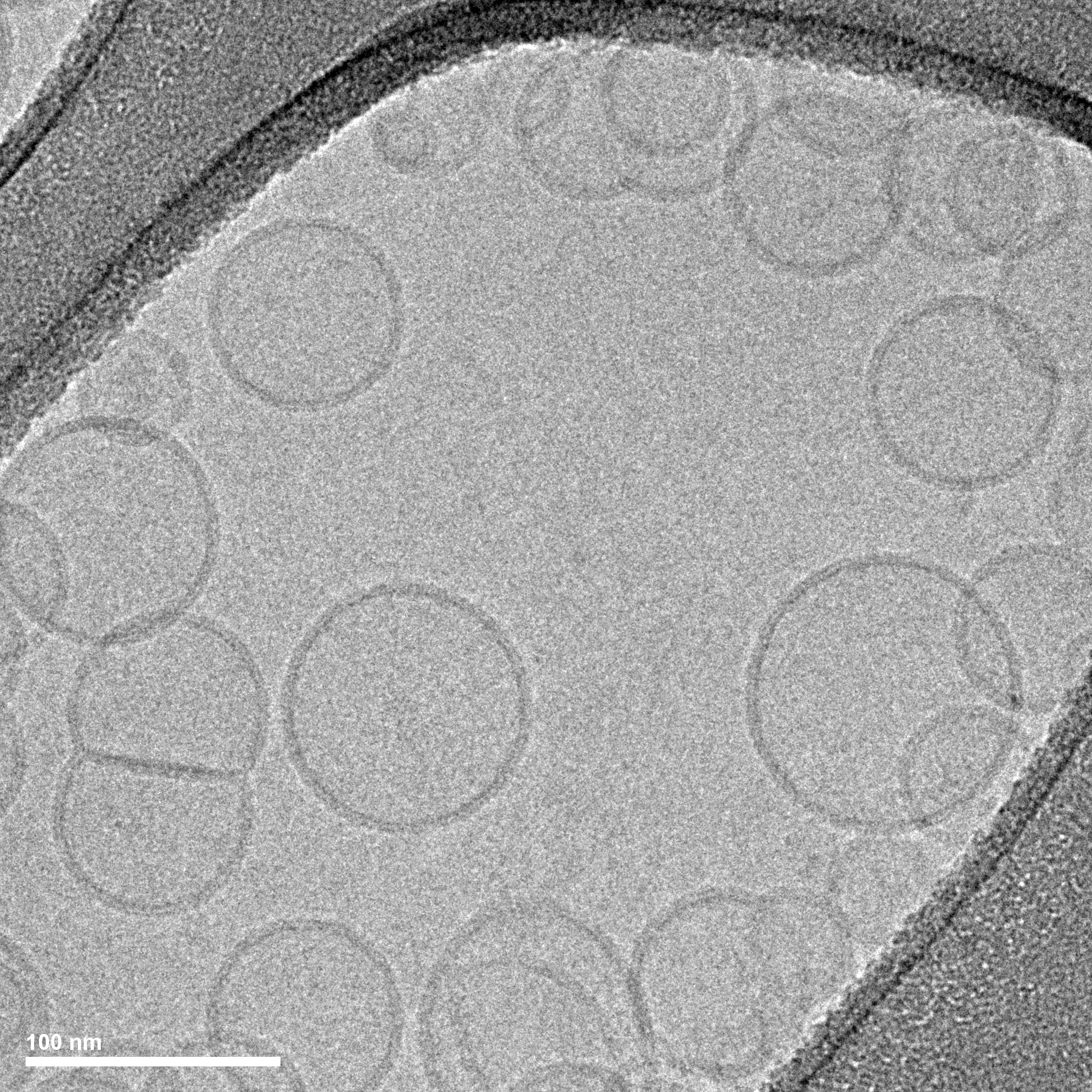

Supplement: Figure 2—source data 1. — This zip archive contains all cryo-EM images used for the quantitative analyses shown in Fig. 2. The folder named “No_Ca++” contains the images before Ca++ addition (individual files are named P3_1_**. tif or jpg), and folder named “With_Ca++” contains the images ∼35s after Ca++ addition (individual files are named P3_3_**.tif or jpg). Images were collected in low dose conditions at 200 kV acceleration voltage on a CM200 FEG electron microscope (FEI) with a 2k × 2k Gatan UltraScan 1000 camera, at 50,000× magnification and 1.5 mm underfocus. The full resolution data were exported as 16 bit “tif” files (2048 × 2048 pixels, scale 0.2 nm/pixel at specimen (the corresponding files have the extension “tif”). Note that these files cannot not be viewed with a standard picture viewer, but must be viewed with a program, such as “ImageJ”. To facilitate easier viewing, the original images were converted to smaller (1024×1024, 0.4 nm/pixel), contrast adjusted jpeg images (8 bits) for easy and immediate visualization with commonly used picture viewers (the corresponding files have the extension “jpg”). DOI: http://dx.doi.org/10.7554/eLife.00109.005 [file elife00109s001.zip › elife00109s001/NO_Ca++/P3_1_06.tif]

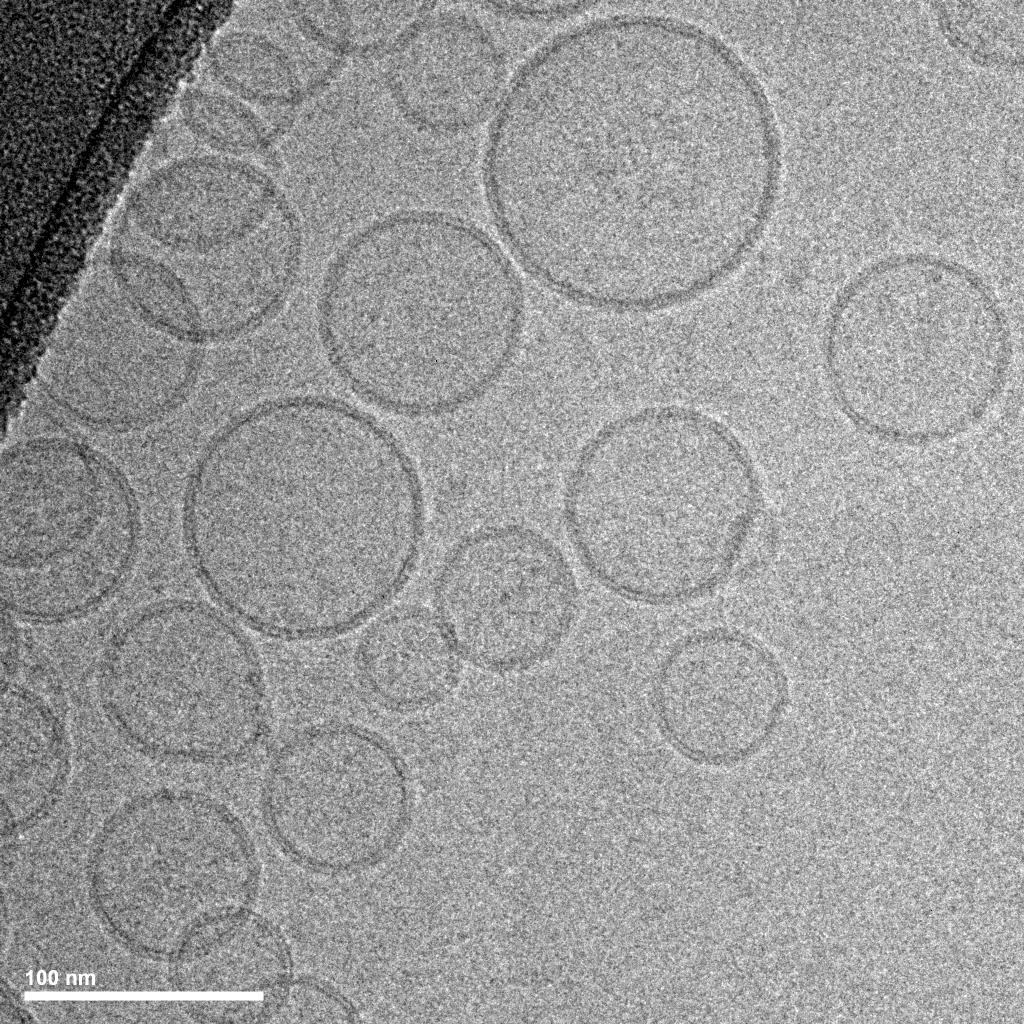

Supplement: Figure 2—source data 1. — This zip archive contains all cryo-EM images used for the quantitative analyses shown in Fig. 2. The folder named “No_Ca++” contains the images before Ca++ addition (individual files are named P3_1_**. tif or jpg), and folder named “With_Ca++” contains the images ∼35s after Ca++ addition (individual files are named P3_3_**.tif or jpg). Images were collected in low dose conditions at 200 kV acceleration voltage on a CM200 FEG electron microscope (FEI) with a 2k × 2k Gatan UltraScan 1000 camera, at 50,000× magnification and 1.5 mm underfocus. The full resolution data were exported as 16 bit “tif” files (2048 × 2048 pixels, scale 0.2 nm/pixel at specimen (the corresponding files have the extension “tif”). Note that these files cannot not be viewed with a standard picture viewer, but must be viewed with a program, such as “ImageJ”. To facilitate easier viewing, the original images were converted to smaller (1024×1024, 0.4 nm/pixel), contrast adjusted jpeg images (8 bits) for easy and immediate visualization with commonly used picture viewers (the corresponding files have the extension “jpg”). DOI: http://dx.doi.org/10.7554/eLife.00109.005 [file elife00109s001.zip › elife00109s001/NO_Ca++/P3_1_07.jpg]

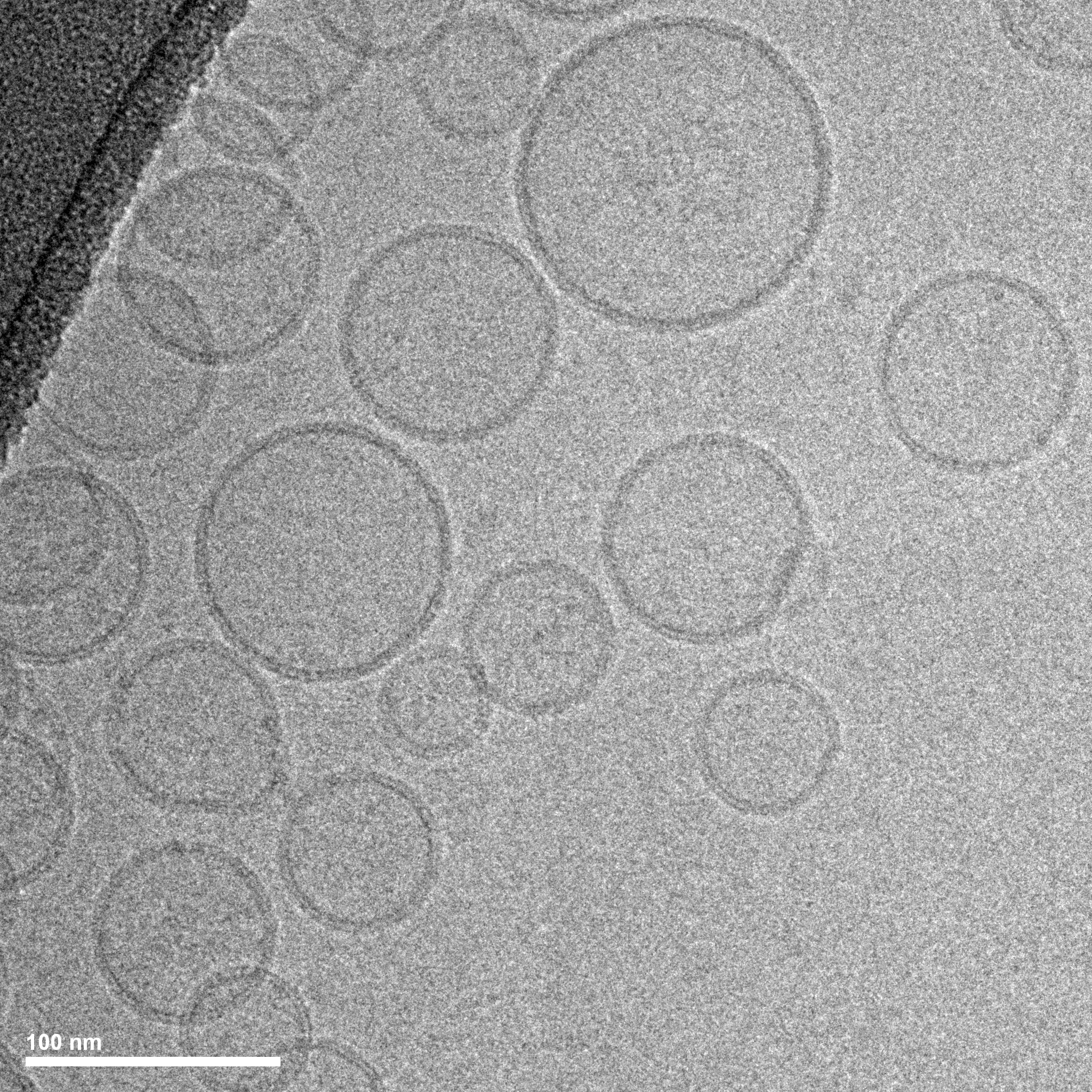

Supplement: Figure 2—source data 1. — This zip archive contains all cryo-EM images used for the quantitative analyses shown in Fig. 2. The folder named “No_Ca++” contains the images before Ca++ addition (individual files are named P3_1_**. tif or jpg), and folder named “With_Ca++” contains the images ∼35s after Ca++ addition (individual files are named P3_3_**.tif or jpg). Images were collected in low dose conditions at 200 kV acceleration voltage on a CM200 FEG electron microscope (FEI) with a 2k × 2k Gatan UltraScan 1000 camera, at 50,000× magnification and 1.5 mm underfocus. The full resolution data were exported as 16 bit “tif” files (2048 × 2048 pixels, scale 0.2 nm/pixel at specimen (the corresponding files have the extension “tif”). Note that these files cannot not be viewed with a standard picture viewer, but must be viewed with a program, such as “ImageJ”. To facilitate easier viewing, the original images were converted to smaller (1024×1024, 0.4 nm/pixel), contrast adjusted jpeg images (8 bits) for easy and immediate visualization with commonly used picture viewers (the corresponding files have the extension “jpg”). DOI: http://dx.doi.org/10.7554/eLife.00109.005 [file elife00109s001.zip › elife00109s001/NO_Ca++/P3_1_07.tif]

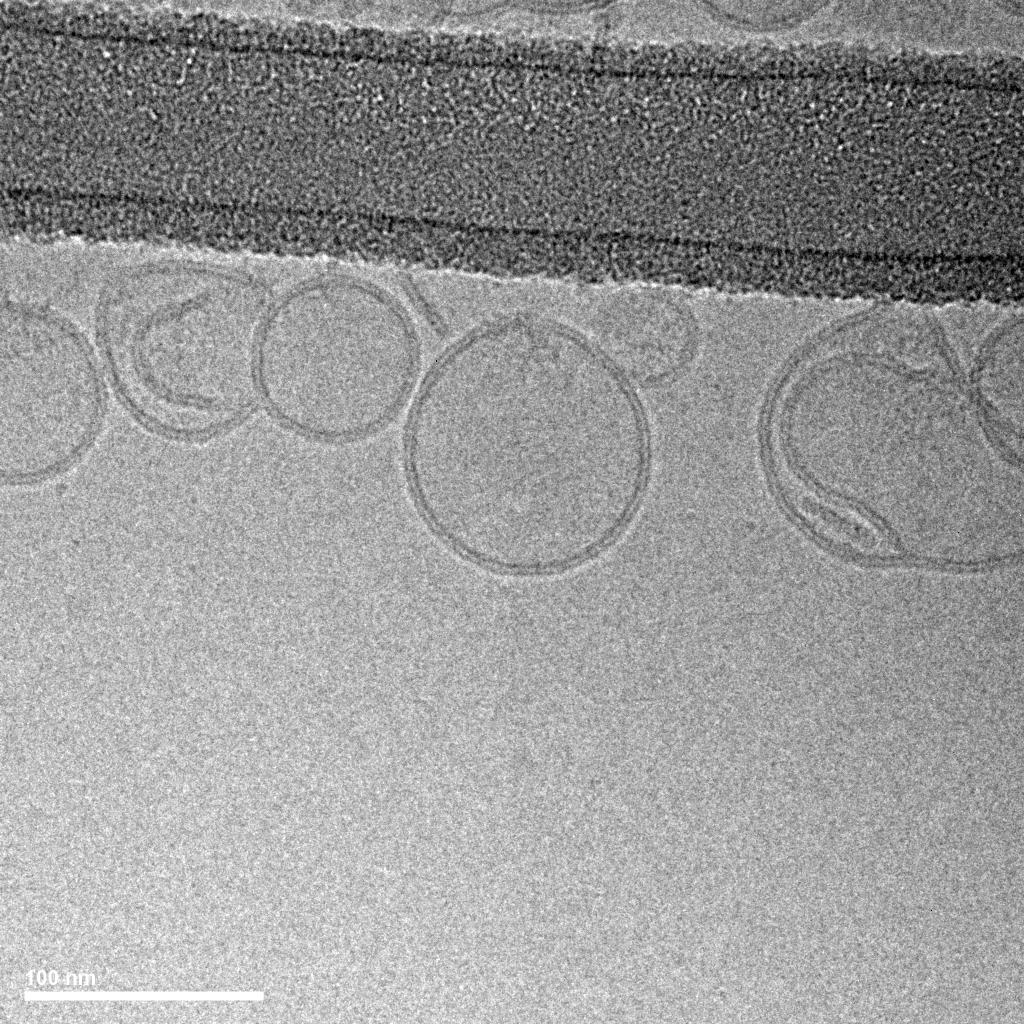

Supplement: Figure 2—source data 1. — This zip archive contains all cryo-EM images used for the quantitative analyses shown in Fig. 2. The folder named “No_Ca++” contains the images before Ca++ addition (individual files are named P3_1_**. tif or jpg), and folder named “With_Ca++” contains the images ∼35s after Ca++ addition (individual files are named P3_3_**.tif or jpg). Images were collected in low dose conditions at 200 kV acceleration voltage on a CM200 FEG electron microscope (FEI) with a 2k × 2k Gatan UltraScan 1000 camera, at 50,000× magnification and 1.5 mm underfocus. The full resolution data were exported as 16 bit “tif” files (2048 × 2048 pixels, scale 0.2 nm/pixel at specimen (the corresponding files have the extension “tif”). Note that these files cannot not be viewed with a standard picture viewer, but must be viewed with a program, such as “ImageJ”. To facilitate easier viewing, the original images were converted to smaller (1024×1024, 0.4 nm/pixel), contrast adjusted jpeg images (8 bits) for easy and immediate visualization with commonly used picture viewers (the corresponding files have the extension “jpg”). DOI: http://dx.doi.org/10.7554/eLife.00109.005 [file elife00109s001.zip › elife00109s001/NO_Ca++/P3_1_08.jpg]

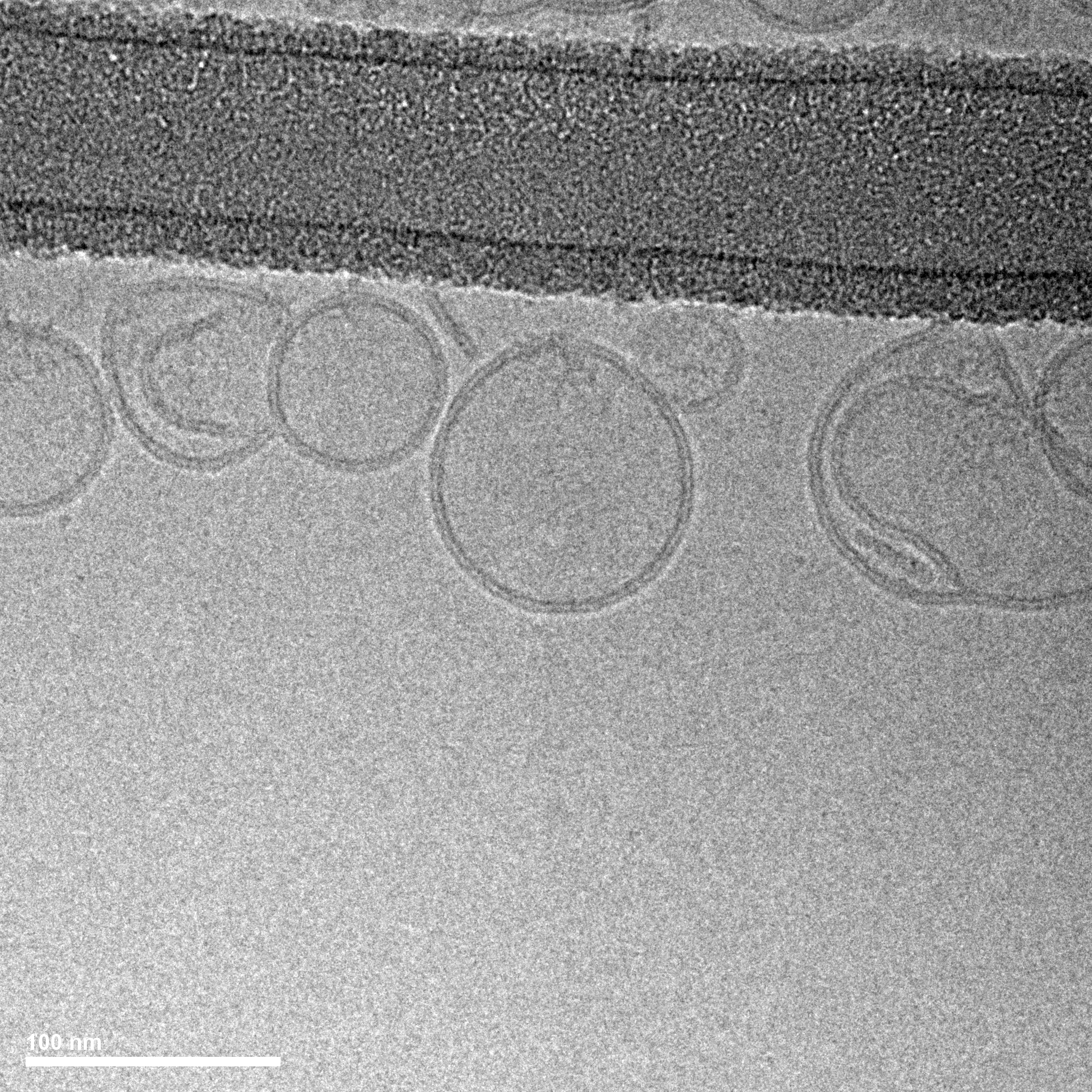

Supplement: Figure 2—source data 1. — This zip archive contains all cryo-EM images used for the quantitative analyses shown in Fig. 2. The folder named “No_Ca++” contains the images before Ca++ addition (individual files are named P3_1_**. tif or jpg), and folder named “With_Ca++” contains the images ∼35s after Ca++ addition (individual files are named P3_3_**.tif or jpg). Images were collected in low dose conditions at 200 kV acceleration voltage on a CM200 FEG electron microscope (FEI) with a 2k × 2k Gatan UltraScan 1000 camera, at 50,000× magnification and 1.5 mm underfocus. The full resolution data were exported as 16 bit “tif” files (2048 × 2048 pixels, scale 0.2 nm/pixel at specimen (the corresponding files have the extension “tif”). Note that these files cannot not be viewed with a standard picture viewer, but must be viewed with a program, such as “ImageJ”. To facilitate easier viewing, the original images were converted to smaller (1024×1024, 0.4 nm/pixel), contrast adjusted jpeg images (8 bits) for easy and immediate visualization with commonly used picture viewers (the corresponding files have the extension “jpg”). DOI: http://dx.doi.org/10.7554/eLife.00109.005 [file elife00109s001.zip › elife00109s001/NO_Ca++/P3_1_08.tif]

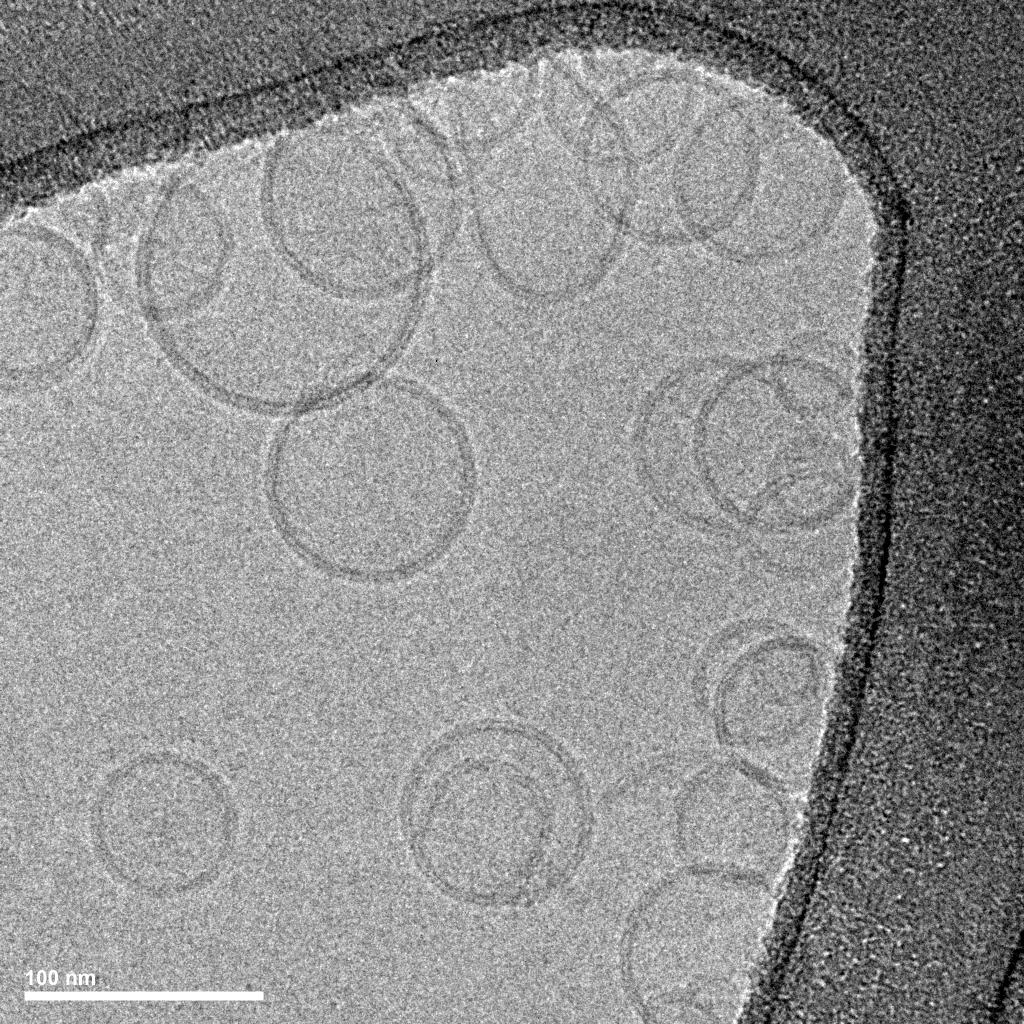

Supplement: Figure 2—source data 1. — This zip archive contains all cryo-EM images used for the quantitative analyses shown in Fig. 2. The folder named “No_Ca++” contains the images before Ca++ addition (individual files are named P3_1_**. tif or jpg), and folder named “With_Ca++” contains the images ∼35s after Ca++ addition (individual files are named P3_3_**.tif or jpg). Images were collected in low dose conditions at 200 kV acceleration voltage on a CM200 FEG electron microscope (FEI) with a 2k × 2k Gatan UltraScan 1000 camera, at 50,000× magnification and 1.5 mm underfocus. The full resolution data were exported as 16 bit “tif” files (2048 × 2048 pixels, scale 0.2 nm/pixel at specimen (the corresponding files have the extension “tif”). Note that these files cannot not be viewed with a standard picture viewer, but must be viewed with a program, such as “ImageJ”. To facilitate easier viewing, the original images were converted to smaller (1024×1024, 0.4 nm/pixel), contrast adjusted jpeg images (8 bits) for easy and immediate visualization with commonly used picture viewers (the corresponding files have the extension “jpg”). DOI: http://dx.doi.org/10.7554/eLife.00109.005 [file elife00109s001.zip › elife00109s001/NO_Ca++/P3_1_09.jpg]

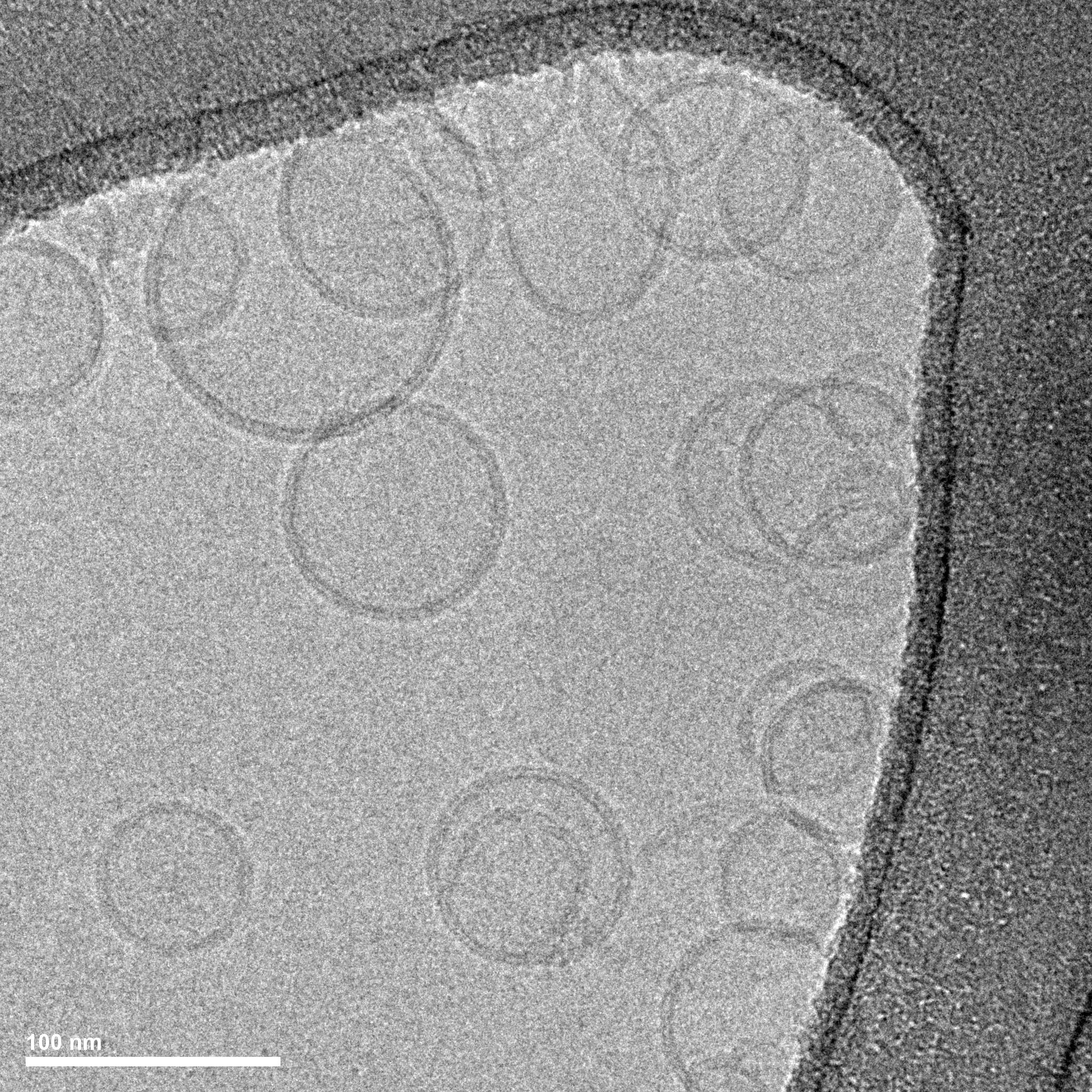

Supplement: Figure 2—source data 1. — This zip archive contains all cryo-EM images used for the quantitative analyses shown in Fig. 2. The folder named “No_Ca++” contains the images before Ca++ addition (individual files are named P3_1_**. tif or jpg), and folder named “With_Ca++” contains the images ∼35s after Ca++ addition (individual files are named P3_3_**.tif or jpg). Images were collected in low dose conditions at 200 kV acceleration voltage on a CM200 FEG electron microscope (FEI) with a 2k × 2k Gatan UltraScan 1000 camera, at 50,000× magnification and 1.5 mm underfocus. The full resolution data were exported as 16 bit “tif” files (2048 × 2048 pixels, scale 0.2 nm/pixel at specimen (the corresponding files have the extension “tif”). Note that these files cannot not be viewed with a standard picture viewer, but must be viewed with a program, such as “ImageJ”. To facilitate easier viewing, the original images were converted to smaller (1024×1024, 0.4 nm/pixel), contrast adjusted jpeg images (8 bits) for easy and immediate visualization with commonly used picture viewers (the corresponding files have the extension “jpg”). DOI: http://dx.doi.org/10.7554/eLife.00109.005 [file elife00109s001.zip › elife00109s001/NO_Ca++/P3_1_09.tif]

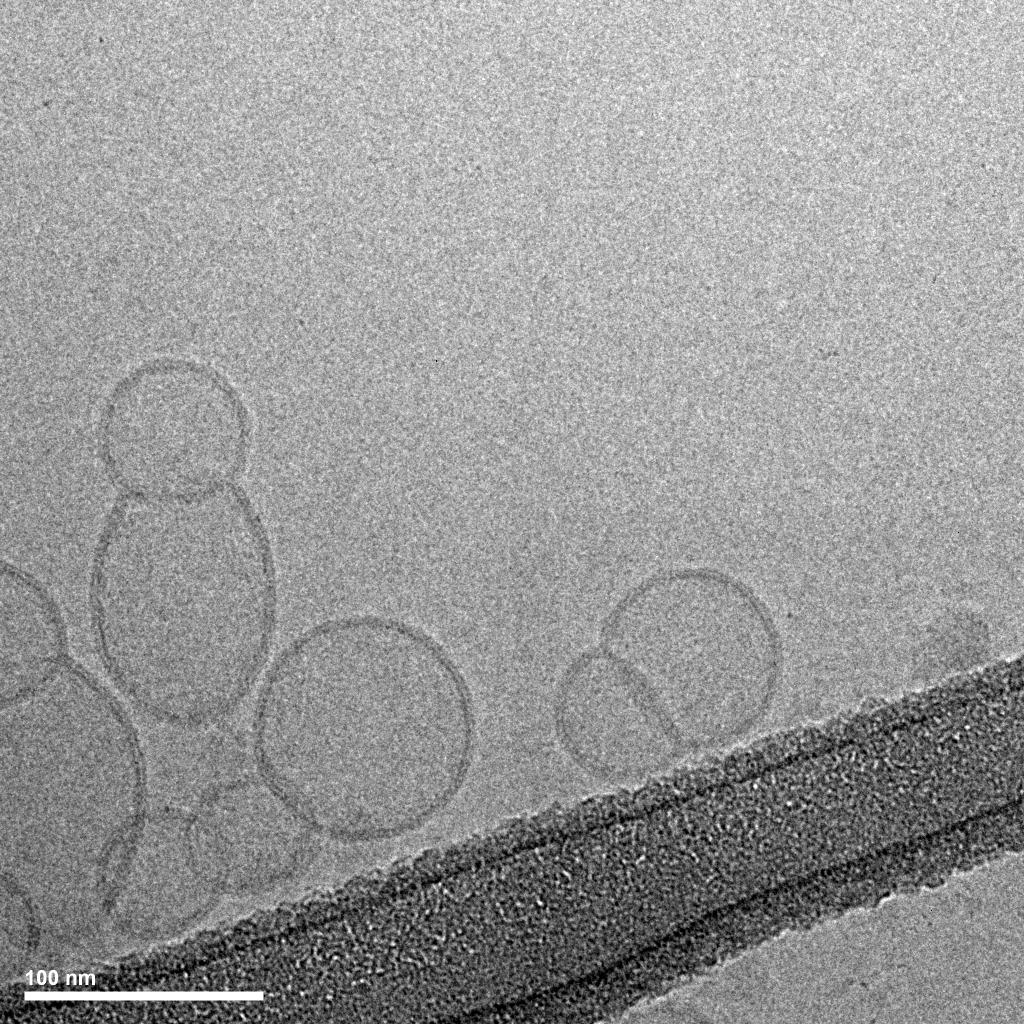

Supplement: Figure 2—source data 1. — This zip archive contains all cryo-EM images used for the quantitative analyses shown in Fig. 2. The folder named “No_Ca++” contains the images before Ca++ addition (individual files are named P3_1_**. tif or jpg), and folder named “With_Ca++” contains the images ∼35s after Ca++ addition (individual files are named P3_3_**.tif or jpg). Images were collected in low dose conditions at 200 kV acceleration voltage on a CM200 FEG electron microscope (FEI) with a 2k × 2k Gatan UltraScan 1000 camera, at 50,000× magnification and 1.5 mm underfocus. The full resolution data were exported as 16 bit “tif” files (2048 × 2048 pixels, scale 0.2 nm/pixel at specimen (the corresponding files have the extension “tif”). Note that these files cannot not be viewed with a standard picture viewer, but must be viewed with a program, such as “ImageJ”. To facilitate easier viewing, the original images were converted to smaller (1024×1024, 0.4 nm/pixel), contrast adjusted jpeg images (8 bits) for easy and immediate visualization with commonly used picture viewers (the corresponding files have the extension “jpg”). DOI: http://dx.doi.org/10.7554/eLife.00109.005 [file elife00109s001.zip › elife00109s001/NO_Ca++/P3_1_10.jpg]

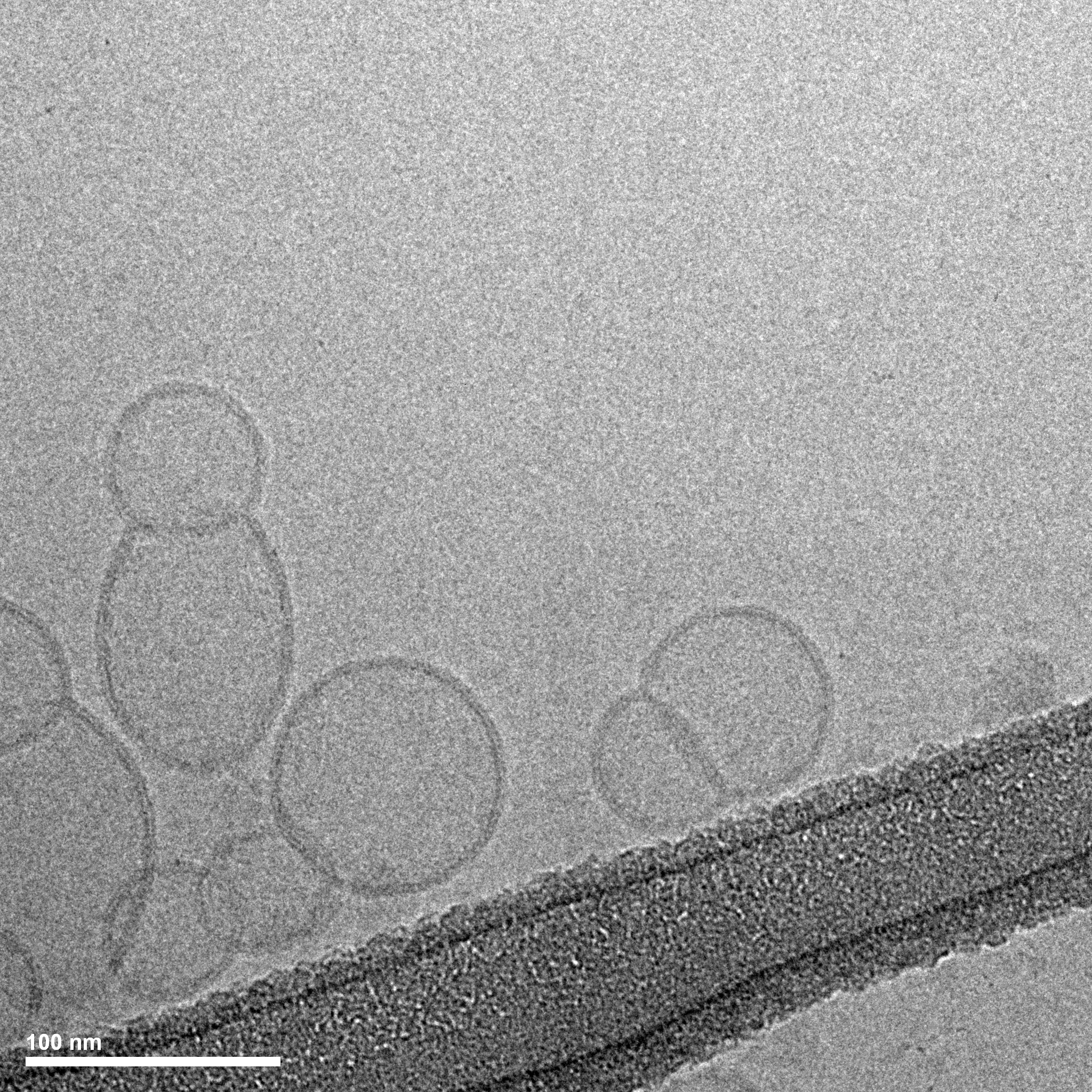

Supplement: Figure 2—source data 1. — This zip archive contains all cryo-EM images used for the quantitative analyses shown in Fig. 2. The folder named “No_Ca++” contains the images before Ca++ addition (individual files are named P3_1_**. tif or jpg), and folder named “With_Ca++” contains the images ∼35s after Ca++ addition (individual files are named P3_3_**.tif or jpg). Images were collected in low dose conditions at 200 kV acceleration voltage on a CM200 FEG electron microscope (FEI) with a 2k × 2k Gatan UltraScan 1000 camera, at 50,000× magnification and 1.5 mm underfocus. The full resolution data were exported as 16 bit “tif” files (2048 × 2048 pixels, scale 0.2 nm/pixel at specimen (the corresponding files have the extension “tif”). Note that these files cannot not be viewed with a standard picture viewer, but must be viewed with a program, such as “ImageJ”. To facilitate easier viewing, the original images were converted to smaller (1024×1024, 0.4 nm/pixel), contrast adjusted jpeg images (8 bits) for easy and immediate visualization with commonly used picture viewers (the corresponding files have the extension “jpg”). DOI: http://dx.doi.org/10.7554/eLife.00109.005 [file elife00109s001.zip › elife00109s001/NO_Ca++/P3_1_10.tif]

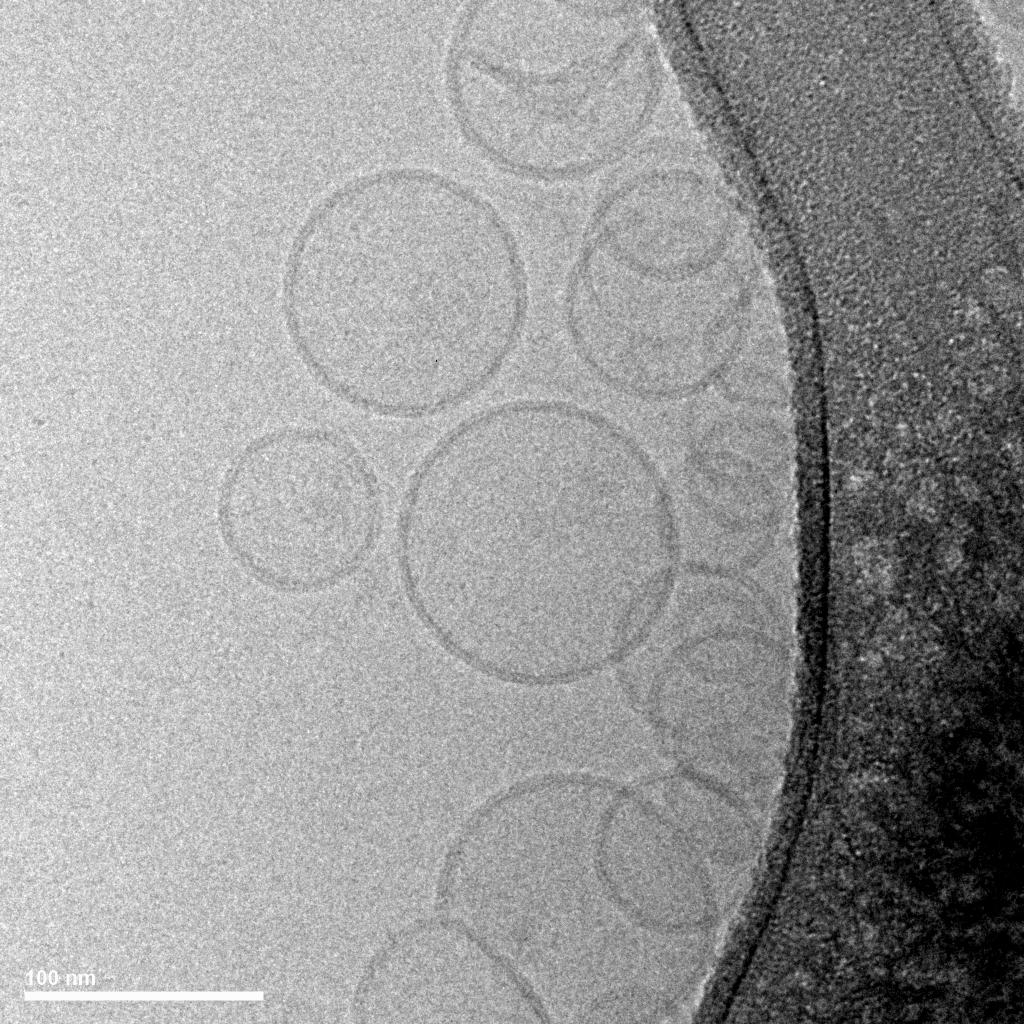

Supplement: Figure 2—source data 1. — This zip archive contains all cryo-EM images used for the quantitative analyses shown in Fig. 2. The folder named “No_Ca++” contains the images before Ca++ addition (individual files are named P3_1_**. tif or jpg), and folder named “With_Ca++” contains the images ∼35s after Ca++ addition (individual files are named P3_3_**.tif or jpg). Images were collected in low dose conditions at 200 kV acceleration voltage on a CM200 FEG electron microscope (FEI) with a 2k × 2k Gatan UltraScan 1000 camera, at 50,000× magnification and 1.5 mm underfocus. The full resolution data were exported as 16 bit “tif” files (2048 × 2048 pixels, scale 0.2 nm/pixel at specimen (the corresponding files have the extension “tif”). Note that these files cannot not be viewed with a standard picture viewer, but must be viewed with a program, such as “ImageJ”. To facilitate easier viewing, the original images were converted to smaller (1024×1024, 0.4 nm/pixel), contrast adjusted jpeg images (8 bits) for easy and immediate visualization with commonly used picture viewers (the corresponding files have the extension “jpg”). DOI: http://dx.doi.org/10.7554/eLife.00109.005 [file elife00109s001.zip › elife00109s001/NO_Ca++/P3_1_11.jpg]

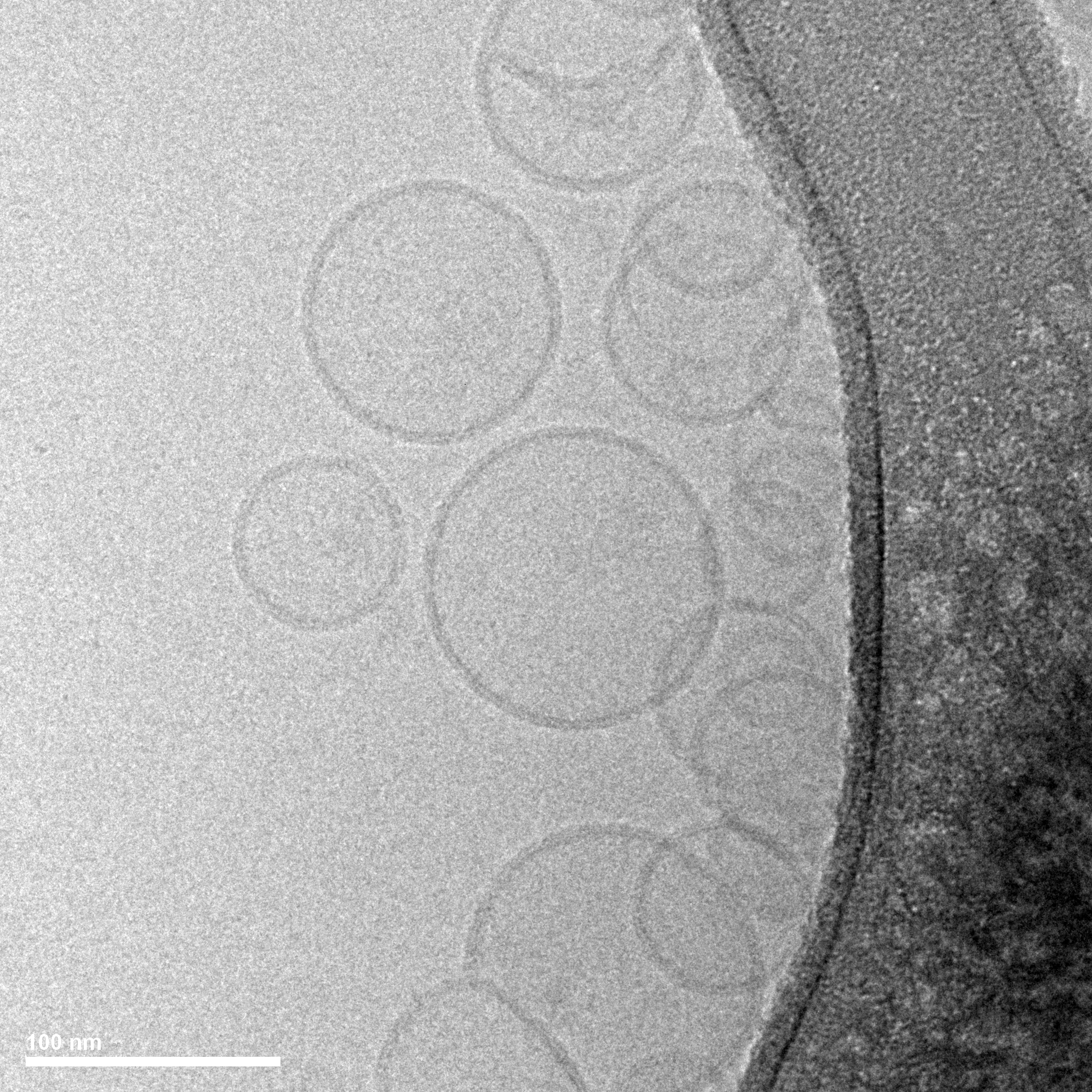

Supplement: Figure 2—source data 1. — This zip archive contains all cryo-EM images used for the quantitative analyses shown in Fig. 2. The folder named “No_Ca++” contains the images before Ca++ addition (individual files are named P3_1_**. tif or jpg), and folder named “With_Ca++” contains the images ∼35s after Ca++ addition (individual files are named P3_3_**.tif or jpg). Images were collected in low dose conditions at 200 kV acceleration voltage on a CM200 FEG electron microscope (FEI) with a 2k × 2k Gatan UltraScan 1000 camera, at 50,000× magnification and 1.5 mm underfocus. The full resolution data were exported as 16 bit “tif” files (2048 × 2048 pixels, scale 0.2 nm/pixel at specimen (the corresponding files have the extension “tif”). Note that these files cannot not be viewed with a standard picture viewer, but must be viewed with a program, such as “ImageJ”. To facilitate easier viewing, the original images were converted to smaller (1024×1024, 0.4 nm/pixel), contrast adjusted jpeg images (8 bits) for easy and immediate visualization with commonly used picture viewers (the corresponding files have the extension “jpg”). DOI: http://dx.doi.org/10.7554/eLife.00109.005 [file elife00109s001.zip › elife00109s001/NO_Ca++/P3_1_11.tif]

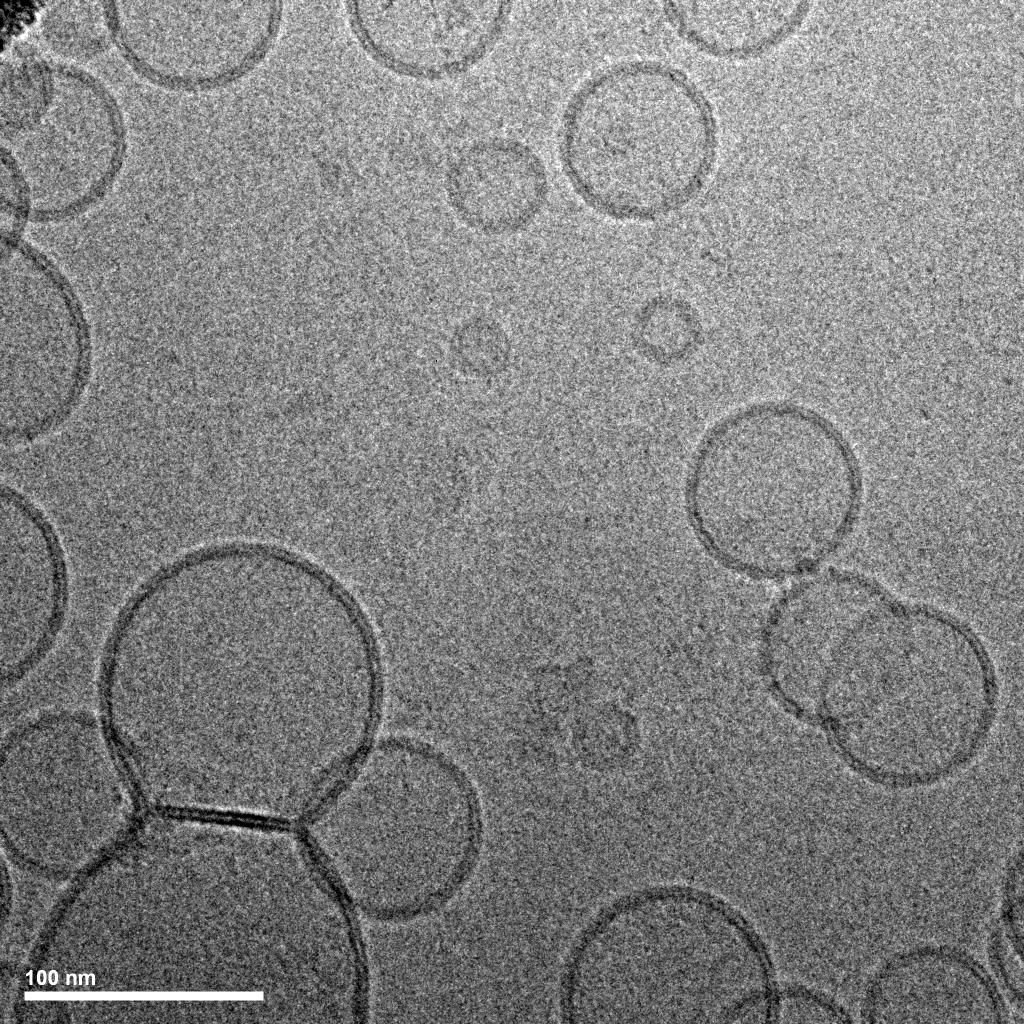

Supplement: Figure 2—source data 1. — This zip archive contains all cryo-EM images used for the quantitative analyses shown in Fig. 2. The folder named “No_Ca++” contains the images before Ca++ addition (individual files are named P3_1_**. tif or jpg), and folder named “With_Ca++” contains the images ∼35s after Ca++ addition (individual files are named P3_3_**.tif or jpg). Images were collected in low dose conditions at 200 kV acceleration voltage on a CM200 FEG electron microscope (FEI) with a 2k × 2k Gatan UltraScan 1000 camera, at 50,000× magnification and 1.5 mm underfocus. The full resolution data were exported as 16 bit “tif” files (2048 × 2048 pixels, scale 0.2 nm/pixel at specimen (the corresponding files have the extension “tif”). Note that these files cannot not be viewed with a standard picture viewer, but must be viewed with a program, such as “ImageJ”. To facilitate easier viewing, the original images were converted to smaller (1024×1024, 0.4 nm/pixel), contrast adjusted jpeg images (8 bits) for easy and immediate visualization with commonly used picture viewers (the corresponding files have the extension “jpg”). DOI: http://dx.doi.org/10.7554/eLife.00109.005 [file elife00109s001.zip › elife00109s001/NO_Ca++/P3_1_12.jpg]

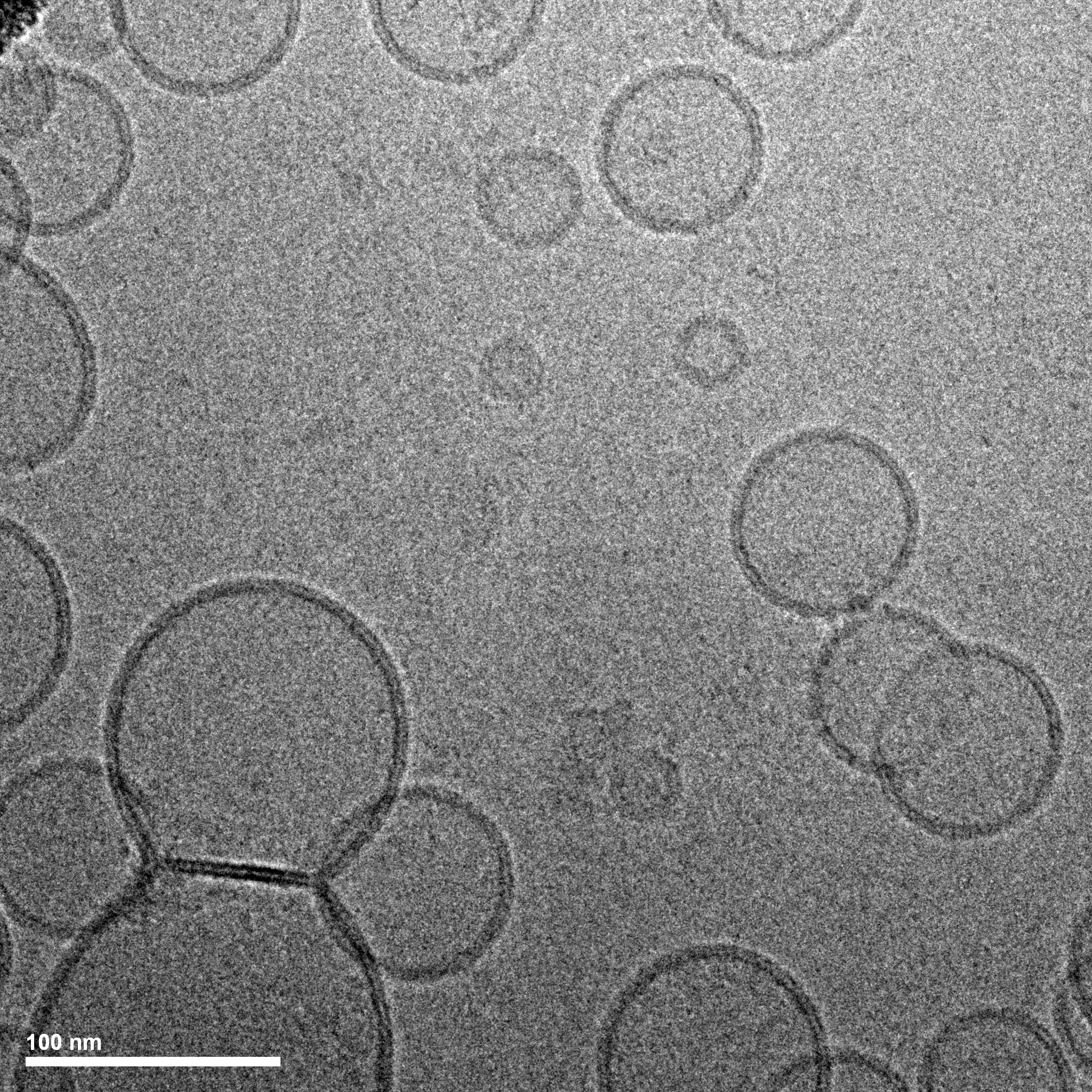

Supplement: Figure 2—source data 1. — This zip archive contains all cryo-EM images used for the quantitative analyses shown in Fig. 2. The folder named “No_Ca++” contains the images before Ca++ addition (individual files are named P3_1_**. tif or jpg), and folder named “With_Ca++” contains the images ∼35s after Ca++ addition (individual files are named P3_3_**.tif or jpg). Images were collected in low dose conditions at 200 kV acceleration voltage on a CM200 FEG electron microscope (FEI) with a 2k × 2k Gatan UltraScan 1000 camera, at 50,000× magnification and 1.5 mm underfocus. The full resolution data were exported as 16 bit “tif” files (2048 × 2048 pixels, scale 0.2 nm/pixel at specimen (the corresponding files have the extension “tif”). Note that these files cannot not be viewed with a standard picture viewer, but must be viewed with a program, such as “ImageJ”. To facilitate easier viewing, the original images were converted to smaller (1024×1024, 0.4 nm/pixel), contrast adjusted jpeg images (8 bits) for easy and immediate visualization with commonly used picture viewers (the corresponding files have the extension “jpg”). DOI: http://dx.doi.org/10.7554/eLife.00109.005 [file elife00109s001.zip › elife00109s001/NO_Ca++/P3_1_12.tif]

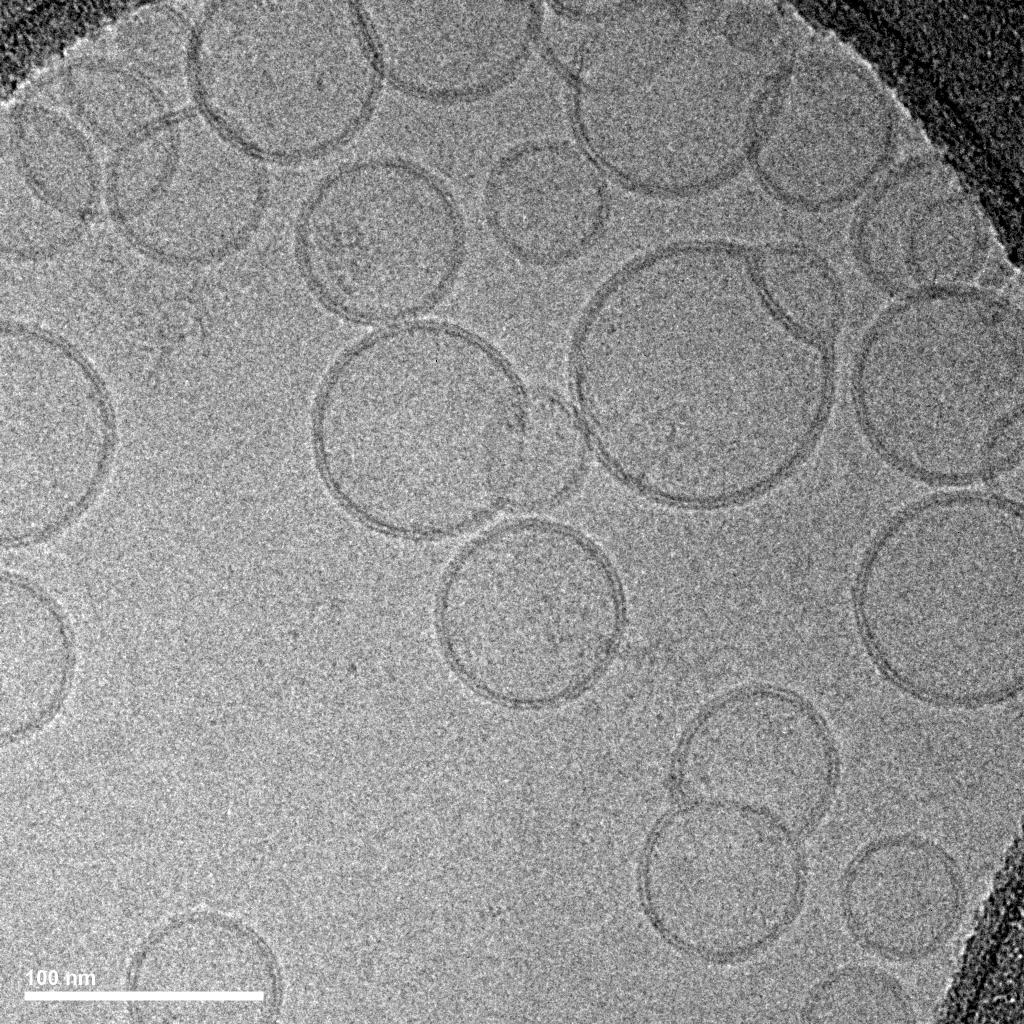

Supplement: Figure 2—source data 1. — This zip archive contains all cryo-EM images used for the quantitative analyses shown in Fig. 2. The folder named “No_Ca++” contains the images before Ca++ addition (individual files are named P3_1_**. tif or jpg), and folder named “With_Ca++” contains the images ∼35s after Ca++ addition (individual files are named P3_3_**.tif or jpg). Images were collected in low dose conditions at 200 kV acceleration voltage on a CM200 FEG electron microscope (FEI) with a 2k × 2k Gatan UltraScan 1000 camera, at 50,000× magnification and 1.5 mm underfocus. The full resolution data were exported as 16 bit “tif” files (2048 × 2048 pixels, scale 0.2 nm/pixel at specimen (the corresponding files have the extension “tif”). Note that these files cannot not be viewed with a standard picture viewer, but must be viewed with a program, such as “ImageJ”. To facilitate easier viewing, the original images were converted to smaller (1024×1024, 0.4 nm/pixel), contrast adjusted jpeg images (8 bits) for easy and immediate visualization with commonly used picture viewers (the corresponding files have the extension “jpg”). DOI: http://dx.doi.org/10.7554/eLife.00109.005 [file elife00109s001.zip › elife00109s001/NO_Ca++/P3_1_13.jpg]

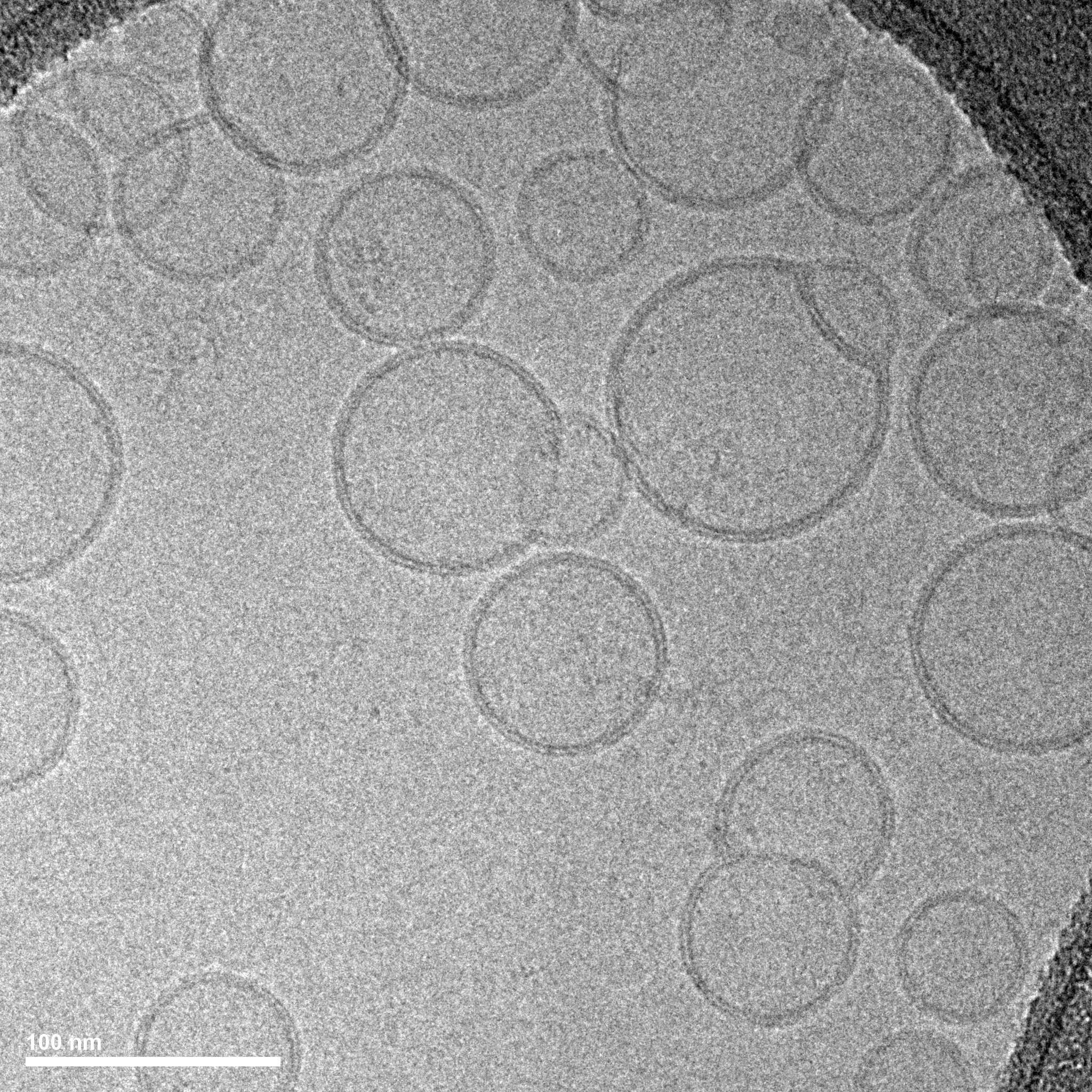

Supplement: Figure 2—source data 1. — This zip archive contains all cryo-EM images used for the quantitative analyses shown in Fig. 2. The folder named “No_Ca++” contains the images before Ca++ addition (individual files are named P3_1_**. tif or jpg), and folder named “With_Ca++” contains the images ∼35s after Ca++ addition (individual files are named P3_3_**.tif or jpg). Images were collected in low dose conditions at 200 kV acceleration voltage on a CM200 FEG electron microscope (FEI) with a 2k × 2k Gatan UltraScan 1000 camera, at 50,000× magnification and 1.5 mm underfocus. The full resolution data were exported as 16 bit “tif” files (2048 × 2048 pixels, scale 0.2 nm/pixel at specimen (the corresponding files have the extension “tif”). Note that these files cannot not be viewed with a standard picture viewer, but must be viewed with a program, such as “ImageJ”. To facilitate easier viewing, the original images were converted to smaller (1024×1024, 0.4 nm/pixel), contrast adjusted jpeg images (8 bits) for easy and immediate visualization with commonly used picture viewers (the corresponding files have the extension “jpg”). DOI: http://dx.doi.org/10.7554/eLife.00109.005 [file elife00109s001.zip › elife00109s001/NO_Ca++/P3_1_13.tif]

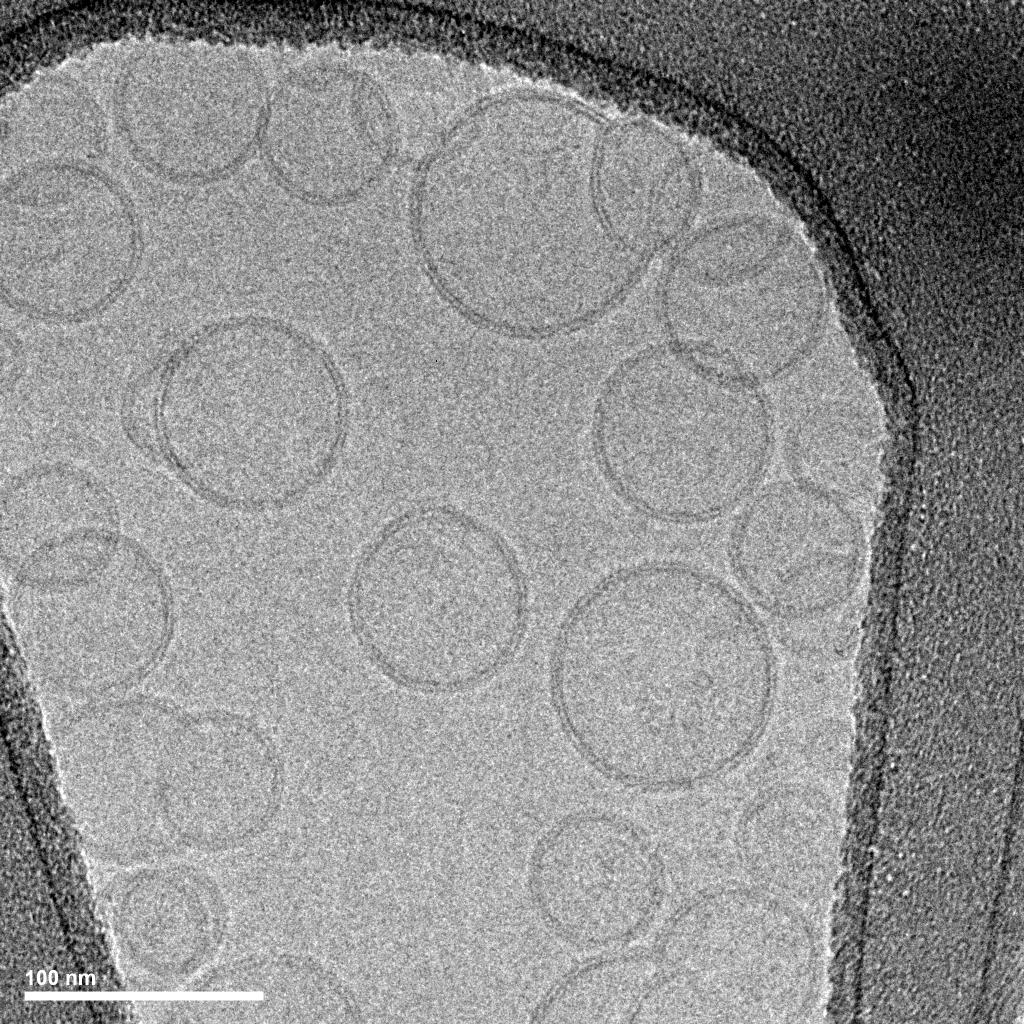

Supplement: Figure 2—source data 1. — This zip archive contains all cryo-EM images used for the quantitative analyses shown in Fig. 2. The folder named “No_Ca++” contains the images before Ca++ addition (individual files are named P3_1_**. tif or jpg), and folder named “With_Ca++” contains the images ∼35s after Ca++ addition (individual files are named P3_3_**.tif or jpg). Images were collected in low dose conditions at 200 kV acceleration voltage on a CM200 FEG electron microscope (FEI) with a 2k × 2k Gatan UltraScan 1000 camera, at 50,000× magnification and 1.5 mm underfocus. The full resolution data were exported as 16 bit “tif” files (2048 × 2048 pixels, scale 0.2 nm/pixel at specimen (the corresponding files have the extension “tif”). Note that these files cannot not be viewed with a standard picture viewer, but must be viewed with a program, such as “ImageJ”. To facilitate easier viewing, the original images were converted to smaller (1024×1024, 0.4 nm/pixel), contrast adjusted jpeg images (8 bits) for easy and immediate visualization with commonly used picture viewers (the corresponding files have the extension “jpg”). DOI: http://dx.doi.org/10.7554/eLife.00109.005 [file elife00109s001.zip › elife00109s001/NO_Ca++/P3_1_15.jpg]

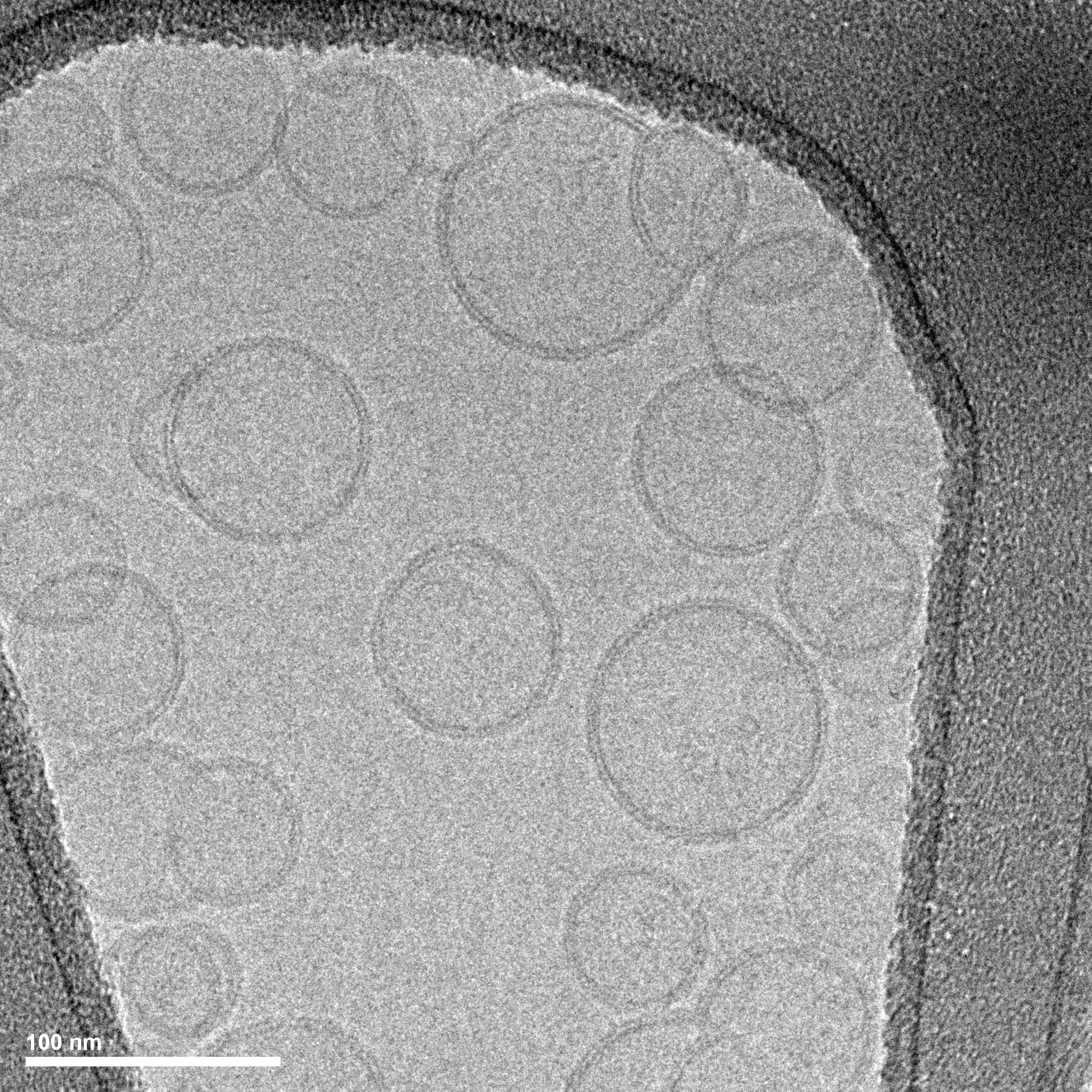

Supplement: Figure 2—source data 1. — This zip archive contains all cryo-EM images used for the quantitative analyses shown in Fig. 2. The folder named “No_Ca++” contains the images before Ca++ addition (individual files are named P3_1_**. tif or jpg), and folder named “With_Ca++” contains the images ∼35s after Ca++ addition (individual files are named P3_3_**.tif or jpg). Images were collected in low dose conditions at 200 kV acceleration voltage on a CM200 FEG electron microscope (FEI) with a 2k × 2k Gatan UltraScan 1000 camera, at 50,000× magnification and 1.5 mm underfocus. The full resolution data were exported as 16 bit “tif” files (2048 × 2048 pixels, scale 0.2 nm/pixel at specimen (the corresponding files have the extension “tif”). Note that these files cannot not be viewed with a standard picture viewer, but must be viewed with a program, such as “ImageJ”. To facilitate easier viewing, the original images were converted to smaller (1024×1024, 0.4 nm/pixel), contrast adjusted jpeg images (8 bits) for easy and immediate visualization with commonly used picture viewers (the corresponding files have the extension “jpg”). DOI: http://dx.doi.org/10.7554/eLife.00109.005 [file elife00109s001.zip › elife00109s001/NO_Ca++/P3_1_15.tif]

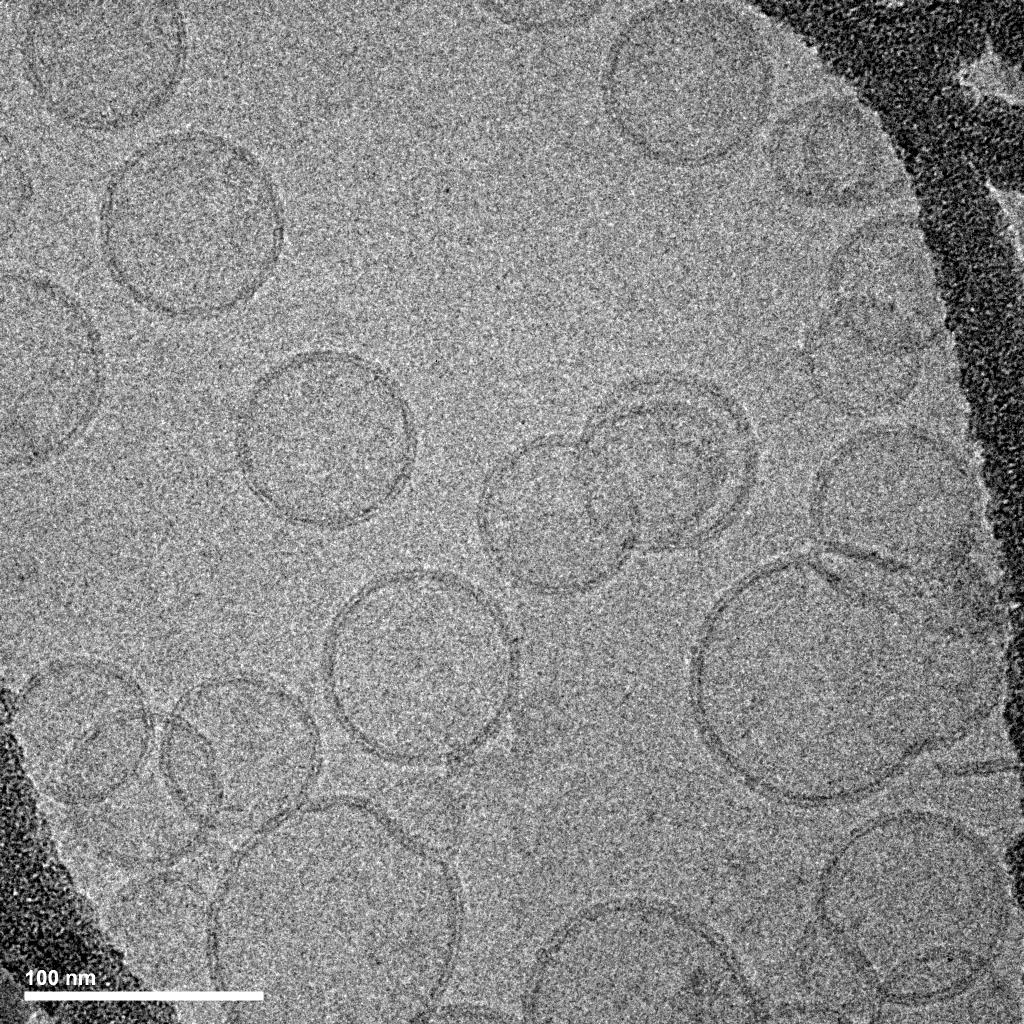

Supplement: Figure 2—source data 1. — This zip archive contains all cryo-EM images used for the quantitative analyses shown in Fig. 2. The folder named “No_Ca++” contains the images before Ca++ addition (individual files are named P3_1_**. tif or jpg), and folder named “With_Ca++” contains the images ∼35s after Ca++ addition (individual files are named P3_3_**.tif or jpg). Images were collected in low dose conditions at 200 kV acceleration voltage on a CM200 FEG electron microscope (FEI) with a 2k × 2k Gatan UltraScan 1000 camera, at 50,000× magnification and 1.5 mm underfocus. The full resolution data were exported as 16 bit “tif” files (2048 × 2048 pixels, scale 0.2 nm/pixel at specimen (the corresponding files have the extension “tif”). Note that these files cannot not be viewed with a standard picture viewer, but must be viewed with a program, such as “ImageJ”. To facilitate easier viewing, the original images were converted to smaller (1024×1024, 0.4 nm/pixel), contrast adjusted jpeg images (8 bits) for easy and immediate visualization with commonly used picture viewers (the corresponding files have the extension “jpg”). DOI: http://dx.doi.org/10.7554/eLife.00109.005 [file elife00109s001.zip › elife00109s001/NO_Ca++/P3_1_16.jpg]

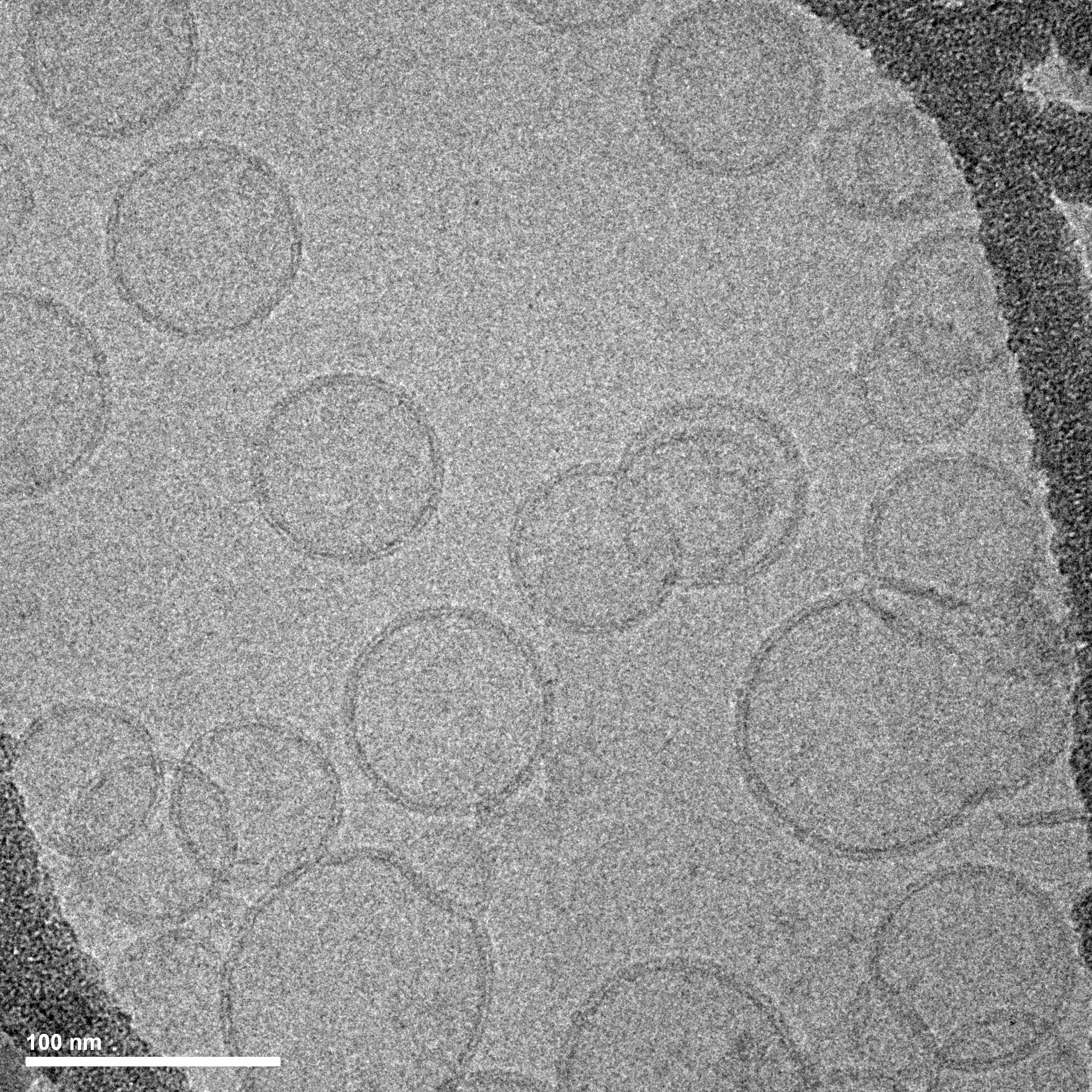

Supplement: Figure 2—source data 1. — This zip archive contains all cryo-EM images used for the quantitative analyses shown in Fig. 2. The folder named “No_Ca++” contains the images before Ca++ addition (individual files are named P3_1_**. tif or jpg), and folder named “With_Ca++” contains the images ∼35s after Ca++ addition (individual files are named P3_3_**.tif or jpg). Images were collected in low dose conditions at 200 kV acceleration voltage on a CM200 FEG electron microscope (FEI) with a 2k × 2k Gatan UltraScan 1000 camera, at 50,000× magnification and 1.5 mm underfocus. The full resolution data were exported as 16 bit “tif” files (2048 × 2048 pixels, scale 0.2 nm/pixel at specimen (the corresponding files have the extension “tif”). Note that these files cannot not be viewed with a standard picture viewer, but must be viewed with a program, such as “ImageJ”. To facilitate easier viewing, the original images were converted to smaller (1024×1024, 0.4 nm/pixel), contrast adjusted jpeg images (8 bits) for easy and immediate visualization with commonly used picture viewers (the corresponding files have the extension “jpg”). DOI: http://dx.doi.org/10.7554/eLife.00109.005 [file elife00109s001.zip › elife00109s001/NO_Ca++/P3_1_16.tif]

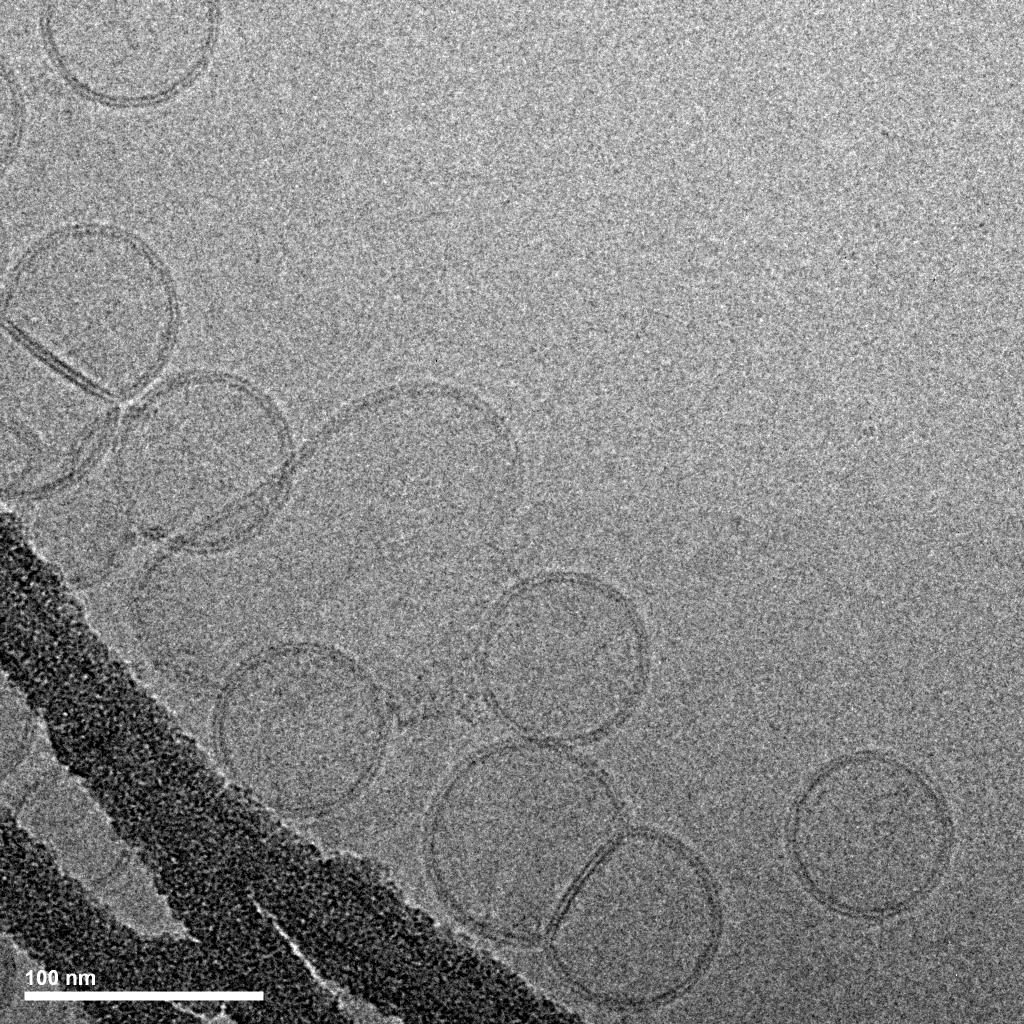

Supplement: Figure 2—source data 1. — This zip archive contains all cryo-EM images used for the quantitative analyses shown in Fig. 2. The folder named “No_Ca++” contains the images before Ca++ addition (individual files are named P3_1_**. tif or jpg), and folder named “With_Ca++” contains the images ∼35s after Ca++ addition (individual files are named P3_3_**.tif or jpg). Images were collected in low dose conditions at 200 kV acceleration voltage on a CM200 FEG electron microscope (FEI) with a 2k × 2k Gatan UltraScan 1000 camera, at 50,000× magnification and 1.5 mm underfocus. The full resolution data were exported as 16 bit “tif” files (2048 × 2048 pixels, scale 0.2 nm/pixel at specimen (the corresponding files have the extension “tif”). Note that these files cannot not be viewed with a standard picture viewer, but must be viewed with a program, such as “ImageJ”. To facilitate easier viewing, the original images were converted to smaller (1024×1024, 0.4 nm/pixel), contrast adjusted jpeg images (8 bits) for easy and immediate visualization with commonly used picture viewers (the corresponding files have the extension “jpg”). DOI: http://dx.doi.org/10.7554/eLife.00109.005 [file elife00109s001.zip › elife00109s001/NO_Ca++/P3_1_18.jpg]

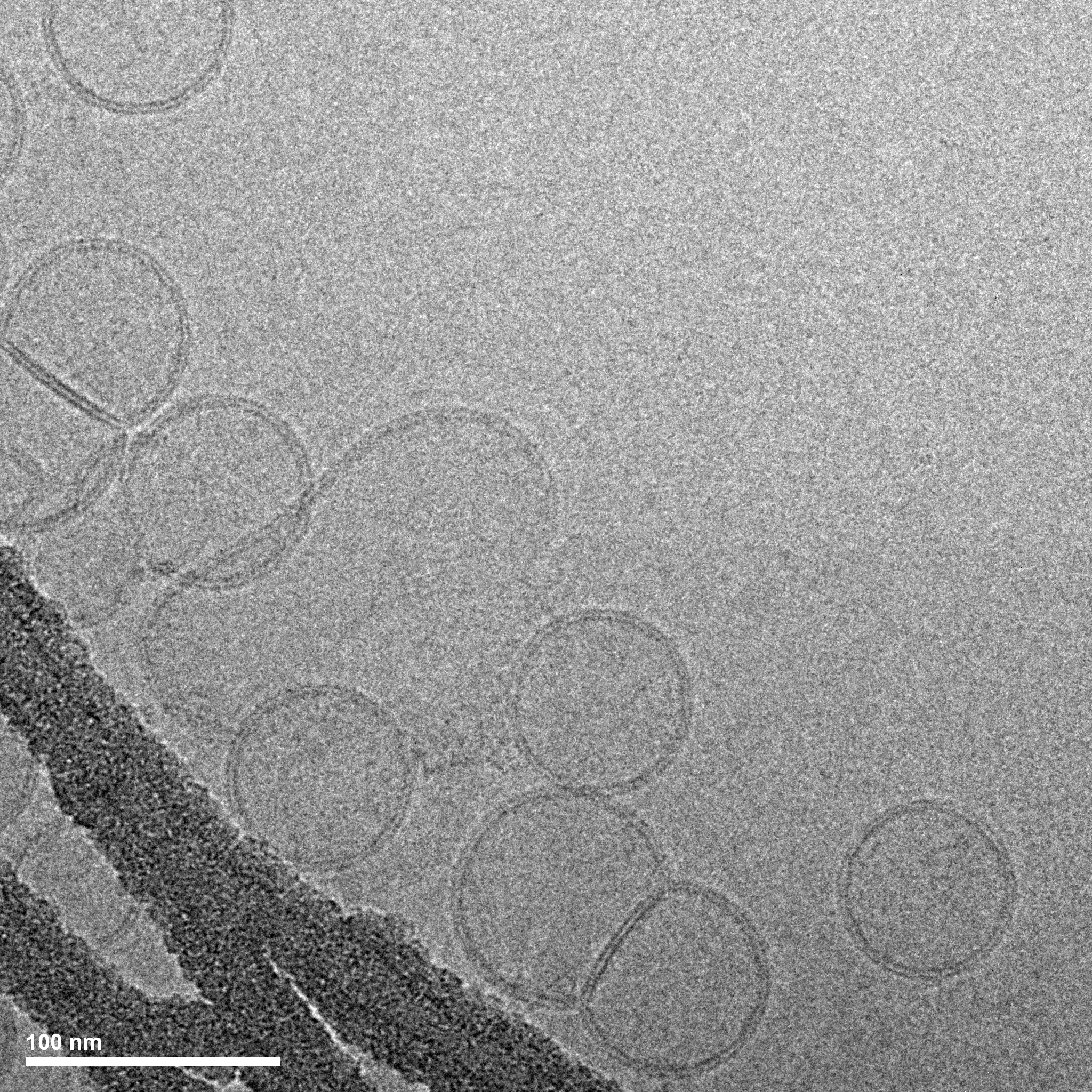

Supplement: Figure 2—source data 1. — This zip archive contains all cryo-EM images used for the quantitative analyses shown in Fig. 2. The folder named “No_Ca++” contains the images before Ca++ addition (individual files are named P3_1_**. tif or jpg), and folder named “With_Ca++” contains the images ∼35s after Ca++ addition (individual files are named P3_3_**.tif or jpg). Images were collected in low dose conditions at 200 kV acceleration voltage on a CM200 FEG electron microscope (FEI) with a 2k × 2k Gatan UltraScan 1000 camera, at 50,000× magnification and 1.5 mm underfocus. The full resolution data were exported as 16 bit “tif” files (2048 × 2048 pixels, scale 0.2 nm/pixel at specimen (the corresponding files have the extension “tif”). Note that these files cannot not be viewed with a standard picture viewer, but must be viewed with a program, such as “ImageJ”. To facilitate easier viewing, the original images were converted to smaller (1024×1024, 0.4 nm/pixel), contrast adjusted jpeg images (8 bits) for easy and immediate visualization with commonly used picture viewers (the corresponding files have the extension “jpg”). DOI: http://dx.doi.org/10.7554/eLife.00109.005 [file elife00109s001.zip › elife00109s001/NO_Ca++/P3_1_18.tif]

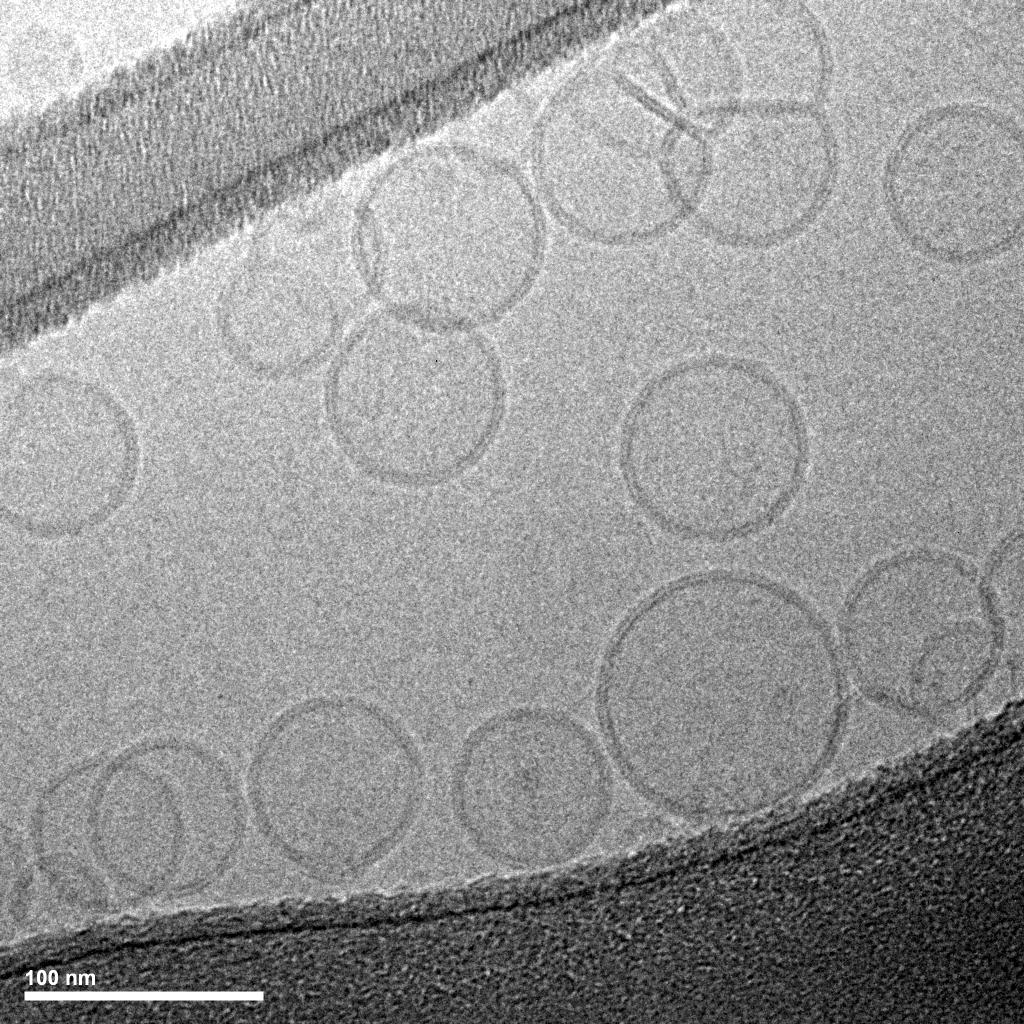

Supplement: Figure 2—source data 1. — This zip archive contains all cryo-EM images used for the quantitative analyses shown in Fig. 2. The folder named “No_Ca++” contains the images before Ca++ addition (individual files are named P3_1_**. tif or jpg), and folder named “With_Ca++” contains the images ∼35s after Ca++ addition (individual files are named P3_3_**.tif or jpg). Images were collected in low dose conditions at 200 kV acceleration voltage on a CM200 FEG electron microscope (FEI) with a 2k × 2k Gatan UltraScan 1000 camera, at 50,000× magnification and 1.5 mm underfocus. The full resolution data were exported as 16 bit “tif” files (2048 × 2048 pixels, scale 0.2 nm/pixel at specimen (the corresponding files have the extension “tif”). Note that these files cannot not be viewed with a standard picture viewer, but must be viewed with a program, such as “ImageJ”. To facilitate easier viewing, the original images were converted to smaller (1024×1024, 0.4 nm/pixel), contrast adjusted jpeg images (8 bits) for easy and immediate visualization with commonly used picture viewers (the corresponding files have the extension “jpg”). DOI: http://dx.doi.org/10.7554/eLife.00109.005 [file elife00109s001.zip › elife00109s001/NO_Ca++/P3_1_20.jpg]

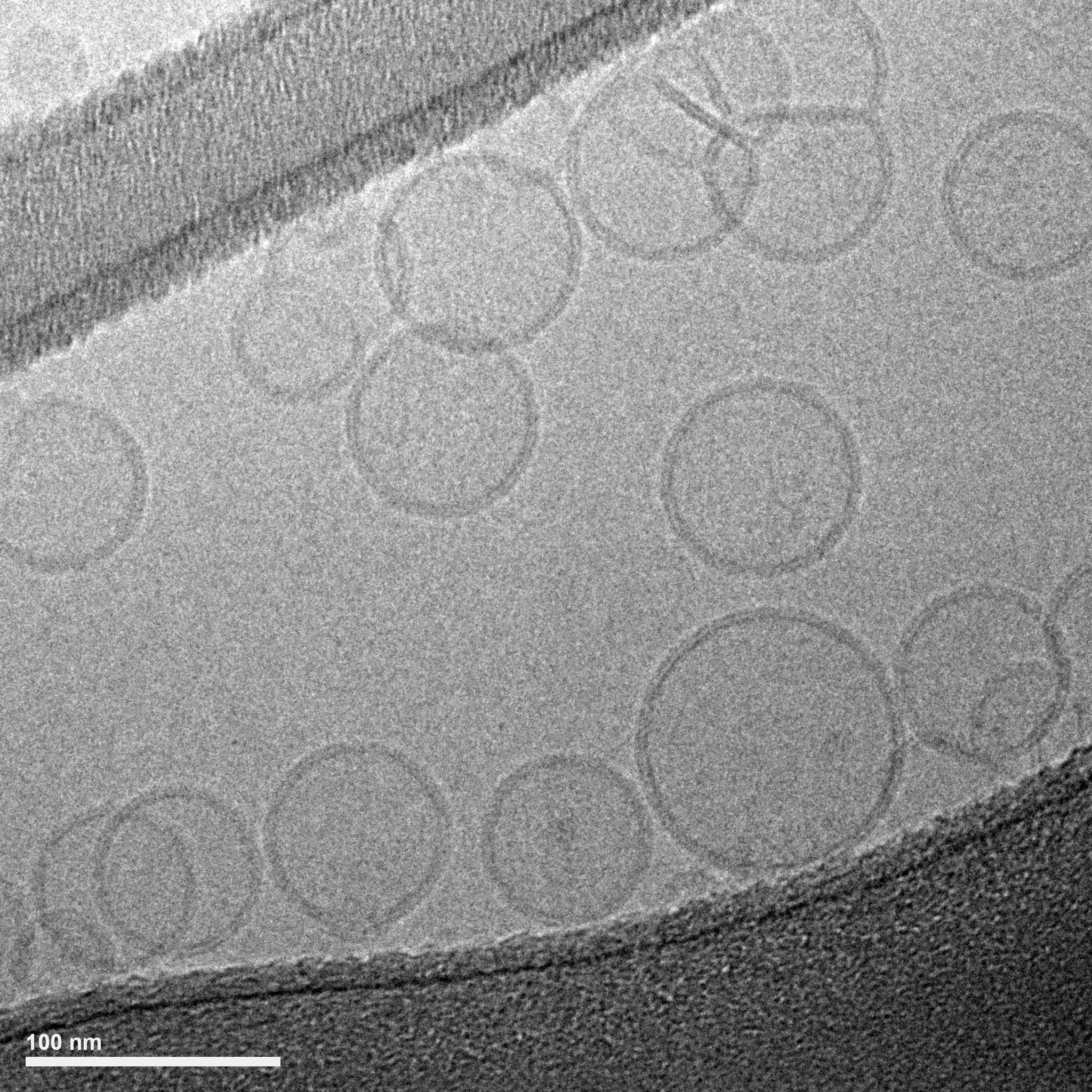

Supplement: Figure 2—source data 1. — This zip archive contains all cryo-EM images used for the quantitative analyses shown in Fig. 2. The folder named “No_Ca++” contains the images before Ca++ addition (individual files are named P3_1_**. tif or jpg), and folder named “With_Ca++” contains the images ∼35s after Ca++ addition (individual files are named P3_3_**.tif or jpg). Images were collected in low dose conditions at 200 kV acceleration voltage on a CM200 FEG electron microscope (FEI) with a 2k × 2k Gatan UltraScan 1000 camera, at 50,000× magnification and 1.5 mm underfocus. The full resolution data were exported as 16 bit “tif” files (2048 × 2048 pixels, scale 0.2 nm/pixel at specimen (the corresponding files have the extension “tif”). Note that these files cannot not be viewed with a standard picture viewer, but must be viewed with a program, such as “ImageJ”. To facilitate easier viewing, the original images were converted to smaller (1024×1024, 0.4 nm/pixel), contrast adjusted jpeg images (8 bits) for easy and immediate visualization with commonly used picture viewers (the corresponding files have the extension “jpg”). DOI: http://dx.doi.org/10.7554/eLife.00109.005 [file elife00109s001.zip › elife00109s001/NO_Ca++/P3_1_20.tif]

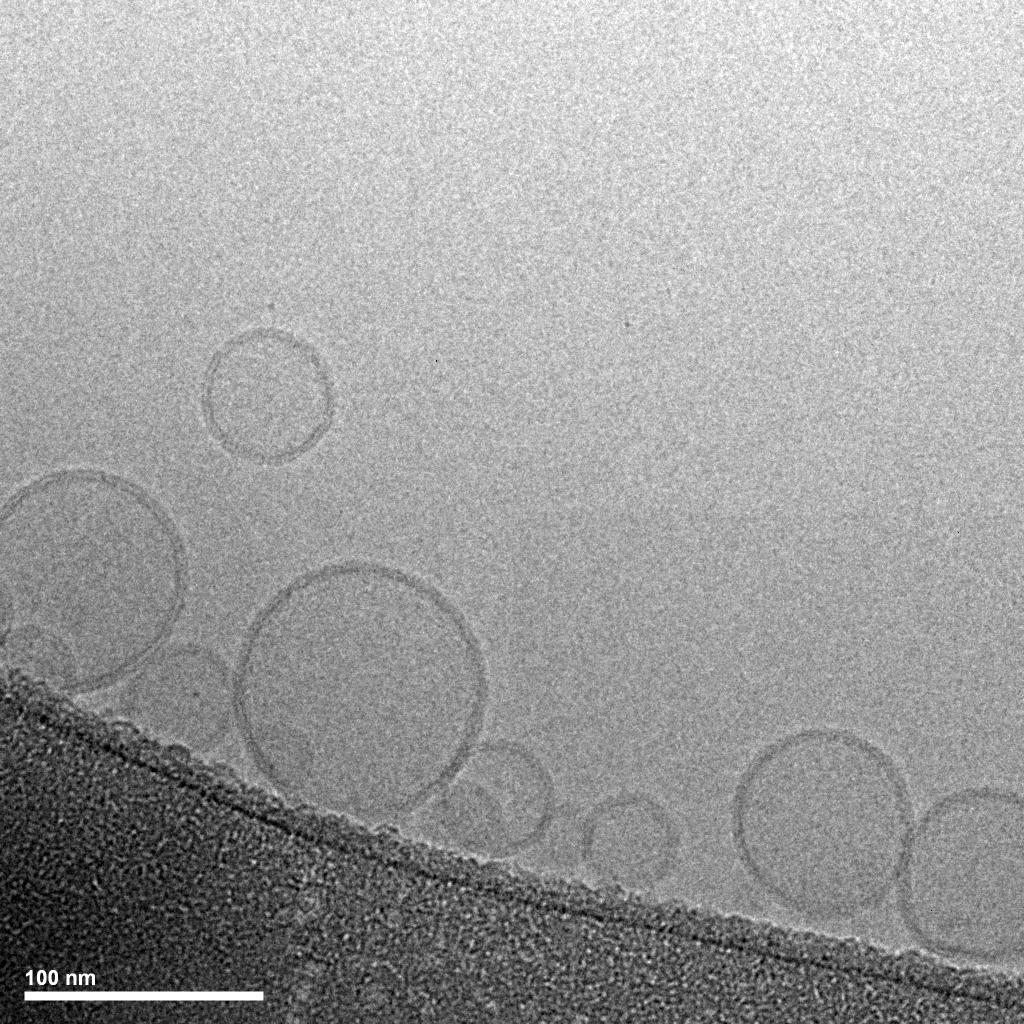

Supplement: Figure 2—source data 1. — This zip archive contains all cryo-EM images used for the quantitative analyses shown in Fig. 2. The folder named “No_Ca++” contains the images before Ca++ addition (individual files are named P3_1_**. tif or jpg), and folder named “With_Ca++” contains the images ∼35s after Ca++ addition (individual files are named P3_3_**.tif or jpg). Images were collected in low dose conditions at 200 kV acceleration voltage on a CM200 FEG electron microscope (FEI) with a 2k × 2k Gatan UltraScan 1000 camera, at 50,000× magnification and 1.5 mm underfocus. The full resolution data were exported as 16 bit “tif” files (2048 × 2048 pixels, scale 0.2 nm/pixel at specimen (the corresponding files have the extension “tif”). Note that these files cannot not be viewed with a standard picture viewer, but must be viewed with a program, such as “ImageJ”. To facilitate easier viewing, the original images were converted to smaller (1024×1024, 0.4 nm/pixel), contrast adjusted jpeg images (8 bits) for easy and immediate visualization with commonly used picture viewers (the corresponding files have the extension “jpg”). DOI: http://dx.doi.org/10.7554/eLife.00109.005 [file elife00109s001.zip › elife00109s001/NO_Ca++/P3_1_21.jpg]

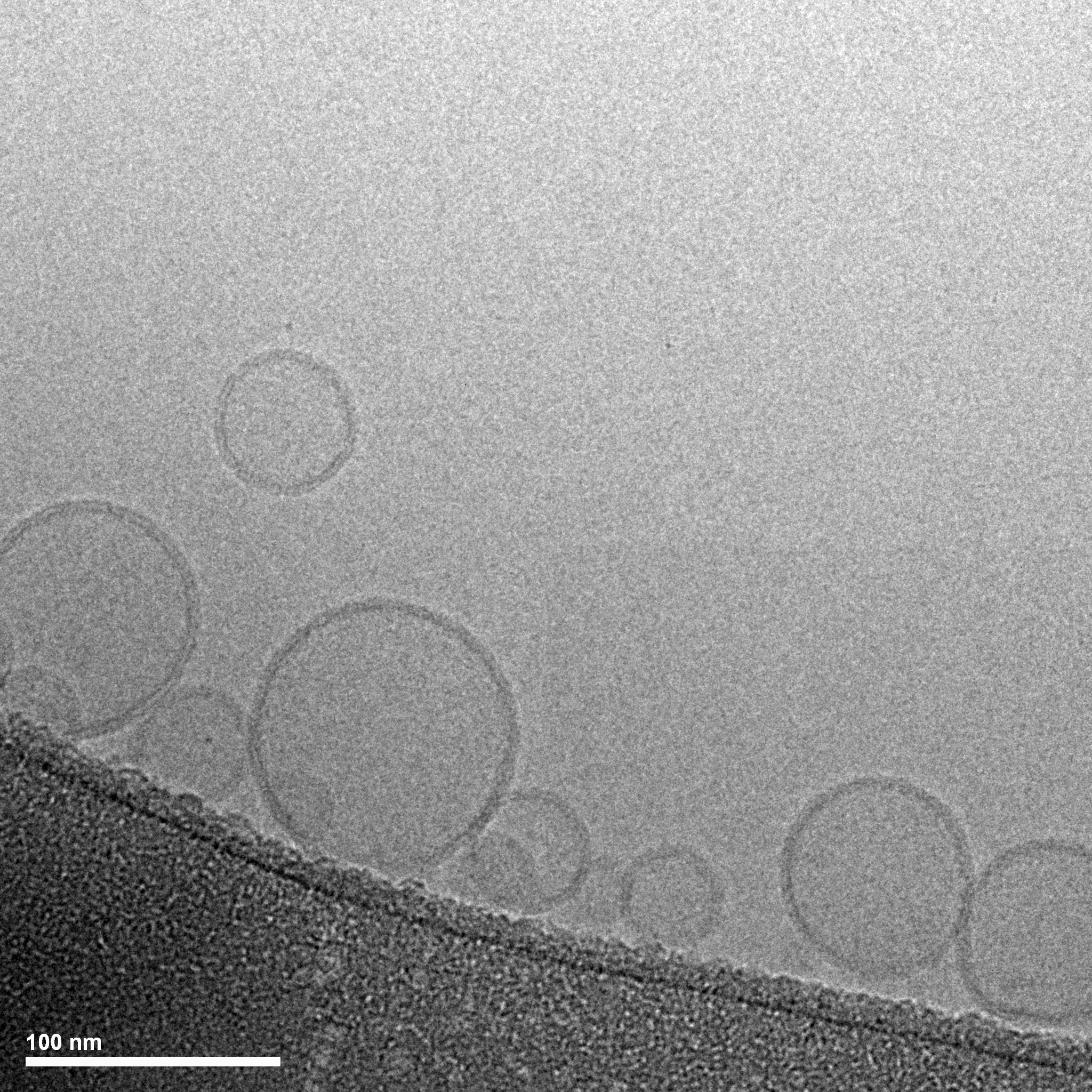

Supplement: Figure 2—source data 1. — This zip archive contains all cryo-EM images used for the quantitative analyses shown in Fig. 2. The folder named “No_Ca++” contains the images before Ca++ addition (individual files are named P3_1_**. tif or jpg), and folder named “With_Ca++” contains the images ∼35s after Ca++ addition (individual files are named P3_3_**.tif or jpg). Images were collected in low dose conditions at 200 kV acceleration voltage on a CM200 FEG electron microscope (FEI) with a 2k × 2k Gatan UltraScan 1000 camera, at 50,000× magnification and 1.5 mm underfocus. The full resolution data were exported as 16 bit “tif” files (2048 × 2048 pixels, scale 0.2 nm/pixel at specimen (the corresponding files have the extension “tif”). Note that these files cannot not be viewed with a standard picture viewer, but must be viewed with a program, such as “ImageJ”. To facilitate easier viewing, the original images were converted to smaller (1024×1024, 0.4 nm/pixel), contrast adjusted jpeg images (8 bits) for easy and immediate visualization with commonly used picture viewers (the corresponding files have the extension “jpg”). DOI: http://dx.doi.org/10.7554/eLife.00109.005 [file elife00109s001.zip › elife00109s001/NO_Ca++/P3_1_21.tif]

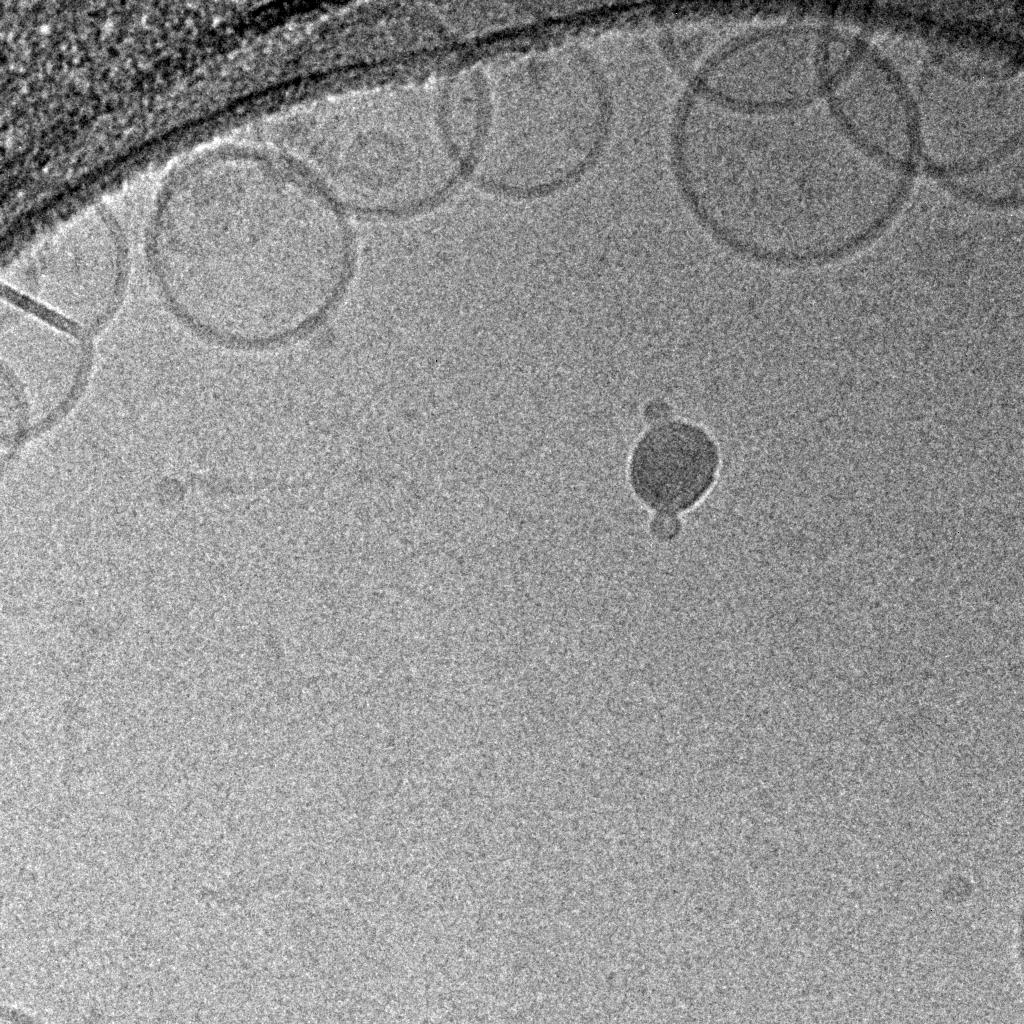

Supplement: Figure 2—source data 1. — This zip archive contains all cryo-EM images used for the quantitative analyses shown in Fig. 2. The folder named “No_Ca++” contains the images before Ca++ addition (individual files are named P3_1_**. tif or jpg), and folder named “With_Ca++” contains the images ∼35s after Ca++ addition (individual files are named P3_3_**.tif or jpg). Images were collected in low dose conditions at 200 kV acceleration voltage on a CM200 FEG electron microscope (FEI) with a 2k × 2k Gatan UltraScan 1000 camera, at 50,000× magnification and 1.5 mm underfocus. The full resolution data were exported as 16 bit “tif” files (2048 × 2048 pixels, scale 0.2 nm/pixel at specimen (the corresponding files have the extension “tif”). Note that these files cannot not be viewed with a standard picture viewer, but must be viewed with a program, such as “ImageJ”. To facilitate easier viewing, the original images were converted to smaller (1024×1024, 0.4 nm/pixel), contrast adjusted jpeg images (8 bits) for easy and immediate visualization with commonly used picture viewers (the corresponding files have the extension “jpg”). DOI: http://dx.doi.org/10.7554/eLife.00109.005 [file elife00109s001.zip › elife00109s001/With_Ca++/P3_3_02.jpg]

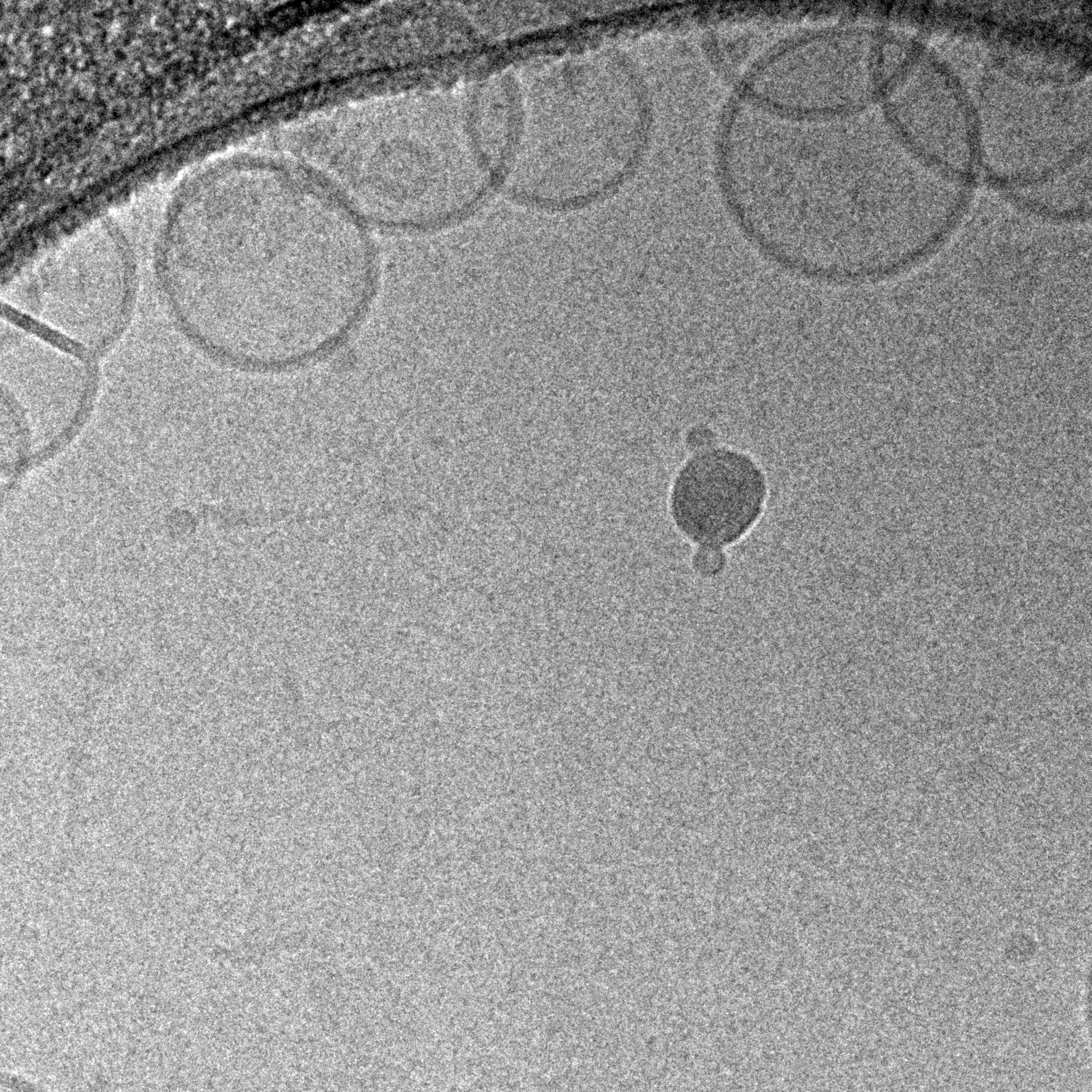

Supplement: Figure 2—source data 1. — This zip archive contains all cryo-EM images used for the quantitative analyses shown in Fig. 2. The folder named “No_Ca++” contains the images before Ca++ addition (individual files are named P3_1_**. tif or jpg), and folder named “With_Ca++” contains the images ∼35s after Ca++ addition (individual files are named P3_3_**.tif or jpg). Images were collected in low dose conditions at 200 kV acceleration voltage on a CM200 FEG electron microscope (FEI) with a 2k × 2k Gatan UltraScan 1000 camera, at 50,000× magnification and 1.5 mm underfocus. The full resolution data were exported as 16 bit “tif” files (2048 × 2048 pixels, scale 0.2 nm/pixel at specimen (the corresponding files have the extension “tif”). Note that these files cannot not be viewed with a standard picture viewer, but must be viewed with a program, such as “ImageJ”. To facilitate easier viewing, the original images were converted to smaller (1024×1024, 0.4 nm/pixel), contrast adjusted jpeg images (8 bits) for easy and immediate visualization with commonly used picture viewers (the corresponding files have the extension “jpg”). DOI: http://dx.doi.org/10.7554/eLife.00109.005 [file elife00109s001.zip › elife00109s001/With_Ca++/P3_3_02.tif]

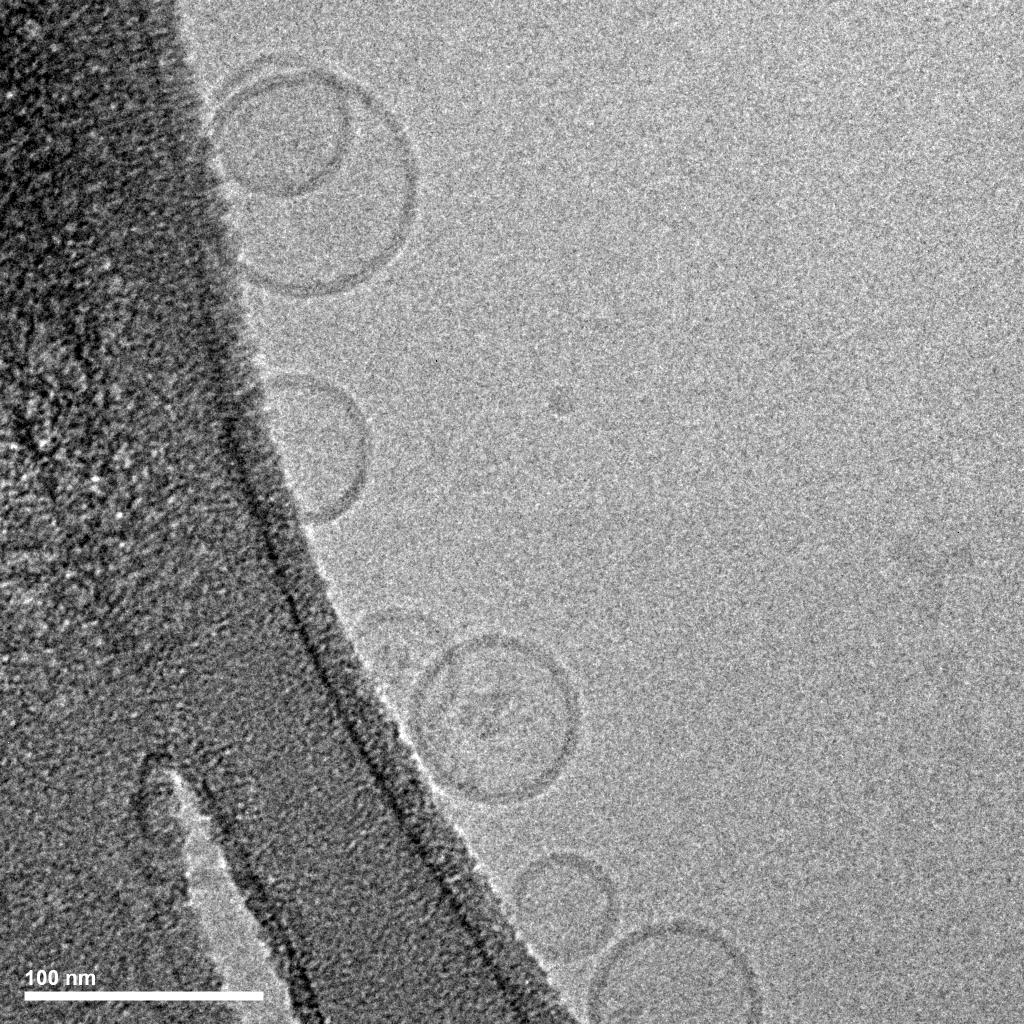

Supplement: Figure 2—source data 1. — This zip archive contains all cryo-EM images used for the quantitative analyses shown in Fig. 2. The folder named “No_Ca++” contains the images before Ca++ addition (individual files are named P3_1_**. tif or jpg), and folder named “With_Ca++” contains the images ∼35s after Ca++ addition (individual files are named P3_3_**.tif or jpg). Images were collected in low dose conditions at 200 kV acceleration voltage on a CM200 FEG electron microscope (FEI) with a 2k × 2k Gatan UltraScan 1000 camera, at 50,000× magnification and 1.5 mm underfocus. The full resolution data were exported as 16 bit “tif” files (2048 × 2048 pixels, scale 0.2 nm/pixel at specimen (the corresponding files have the extension “tif”). Note that these files cannot not be viewed with a standard picture viewer, but must be viewed with a program, such as “ImageJ”. To facilitate easier viewing, the original images were converted to smaller (1024×1024, 0.4 nm/pixel), contrast adjusted jpeg images (8 bits) for easy and immediate visualization with commonly used picture viewers (the corresponding files have the extension “jpg”). DOI: http://dx.doi.org/10.7554/eLife.00109.005 [file elife00109s001.zip › elife00109s001/With_Ca++/P3_3_04.jpg]

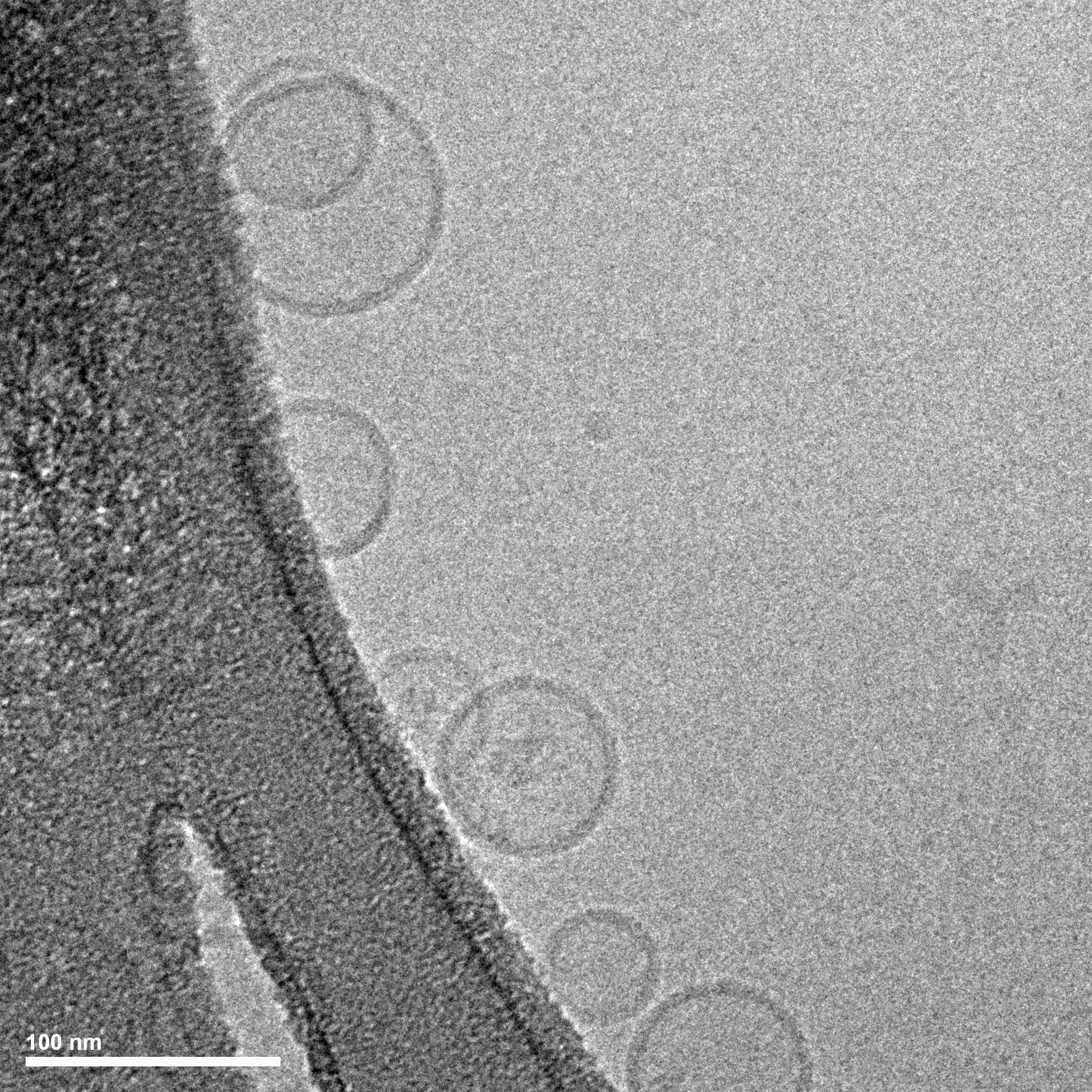

Supplement: Figure 2—source data 1. — This zip archive contains all cryo-EM images used for the quantitative analyses shown in Fig. 2. The folder named “No_Ca++” contains the images before Ca++ addition (individual files are named P3_1_**. tif or jpg), and folder named “With_Ca++” contains the images ∼35s after Ca++ addition (individual files are named P3_3_**.tif or jpg). Images were collected in low dose conditions at 200 kV acceleration voltage on a CM200 FEG electron microscope (FEI) with a 2k × 2k Gatan UltraScan 1000 camera, at 50,000× magnification and 1.5 mm underfocus. The full resolution data were exported as 16 bit “tif” files (2048 × 2048 pixels, scale 0.2 nm/pixel at specimen (the corresponding files have the extension “tif”). Note that these files cannot not be viewed with a standard picture viewer, but must be viewed with a program, such as “ImageJ”. To facilitate easier viewing, the original images were converted to smaller (1024×1024, 0.4 nm/pixel), contrast adjusted jpeg images (8 bits) for easy and immediate visualization with commonly used picture viewers (the corresponding files have the extension “jpg”). DOI: http://dx.doi.org/10.7554/eLife.00109.005 [file elife00109s001.zip › elife00109s001/With_Ca++/P3_3_04.tif]

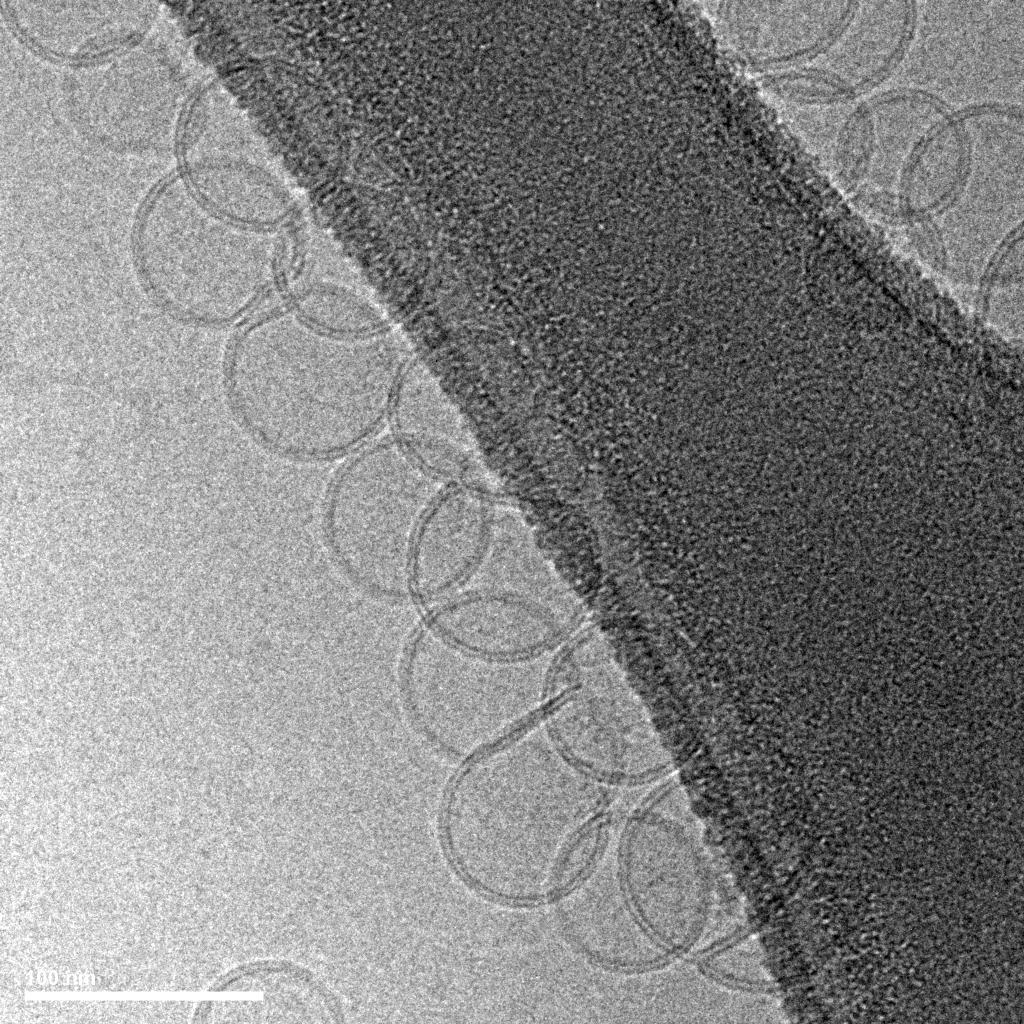

Supplement: Figure 2—source data 1. — This zip archive contains all cryo-EM images used for the quantitative analyses shown in Fig. 2. The folder named “No_Ca++” contains the images before Ca++ addition (individual files are named P3_1_**. tif or jpg), and folder named “With_Ca++” contains the images ∼35s after Ca++ addition (individual files are named P3_3_**.tif or jpg). Images were collected in low dose conditions at 200 kV acceleration voltage on a CM200 FEG electron microscope (FEI) with a 2k × 2k Gatan UltraScan 1000 camera, at 50,000× magnification and 1.5 mm underfocus. The full resolution data were exported as 16 bit “tif” files (2048 × 2048 pixels, scale 0.2 nm/pixel at specimen (the corresponding files have the extension “tif”). Note that these files cannot not be viewed with a standard picture viewer, but must be viewed with a program, such as “ImageJ”. To facilitate easier viewing, the original images were converted to smaller (1024×1024, 0.4 nm/pixel), contrast adjusted jpeg images (8 bits) for easy and immediate visualization with commonly used picture viewers (the corresponding files have the extension “jpg”). DOI: http://dx.doi.org/10.7554/eLife.00109.005 [file elife00109s001.zip › elife00109s001/With_Ca++/P3_3_06.jpg]

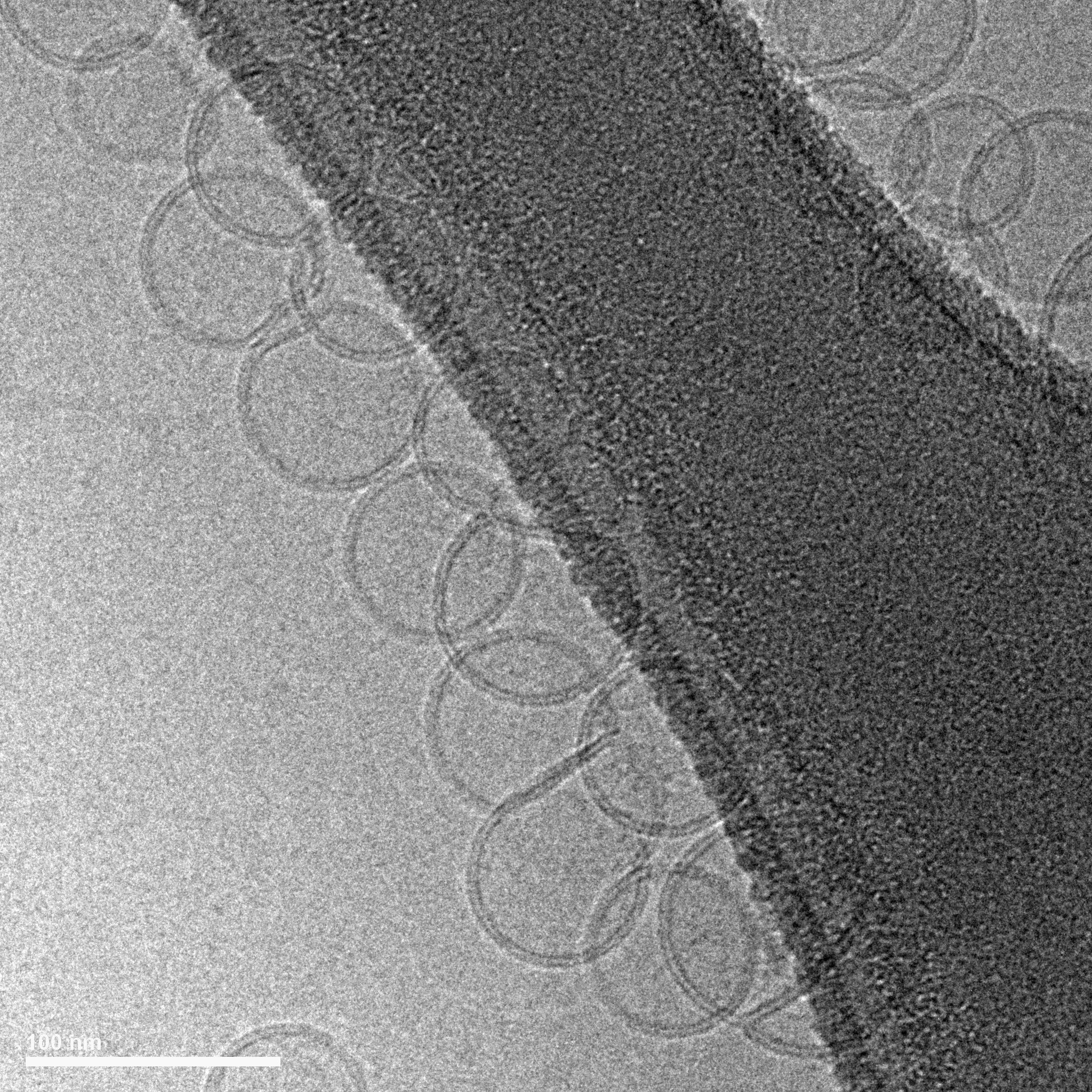

Supplement: Figure 2—source data 1. — This zip archive contains all cryo-EM images used for the quantitative analyses shown in Fig. 2. The folder named “No_Ca++” contains the images before Ca++ addition (individual files are named P3_1_**. tif or jpg), and folder named “With_Ca++” contains the images ∼35s after Ca++ addition (individual files are named P3_3_**.tif or jpg). Images were collected in low dose conditions at 200 kV acceleration voltage on a CM200 FEG electron microscope (FEI) with a 2k × 2k Gatan UltraScan 1000 camera, at 50,000× magnification and 1.5 mm underfocus. The full resolution data were exported as 16 bit “tif” files (2048 × 2048 pixels, scale 0.2 nm/pixel at specimen (the corresponding files have the extension “tif”). Note that these files cannot not be viewed with a standard picture viewer, but must be viewed with a program, such as “ImageJ”. To facilitate easier viewing, the original images were converted to smaller (1024×1024, 0.4 nm/pixel), contrast adjusted jpeg images (8 bits) for easy and immediate visualization with commonly used picture viewers (the corresponding files have the extension “jpg”). DOI: http://dx.doi.org/10.7554/eLife.00109.005 [file elife00109s001.zip › elife00109s001/With_Ca++/P3_3_06.tif]

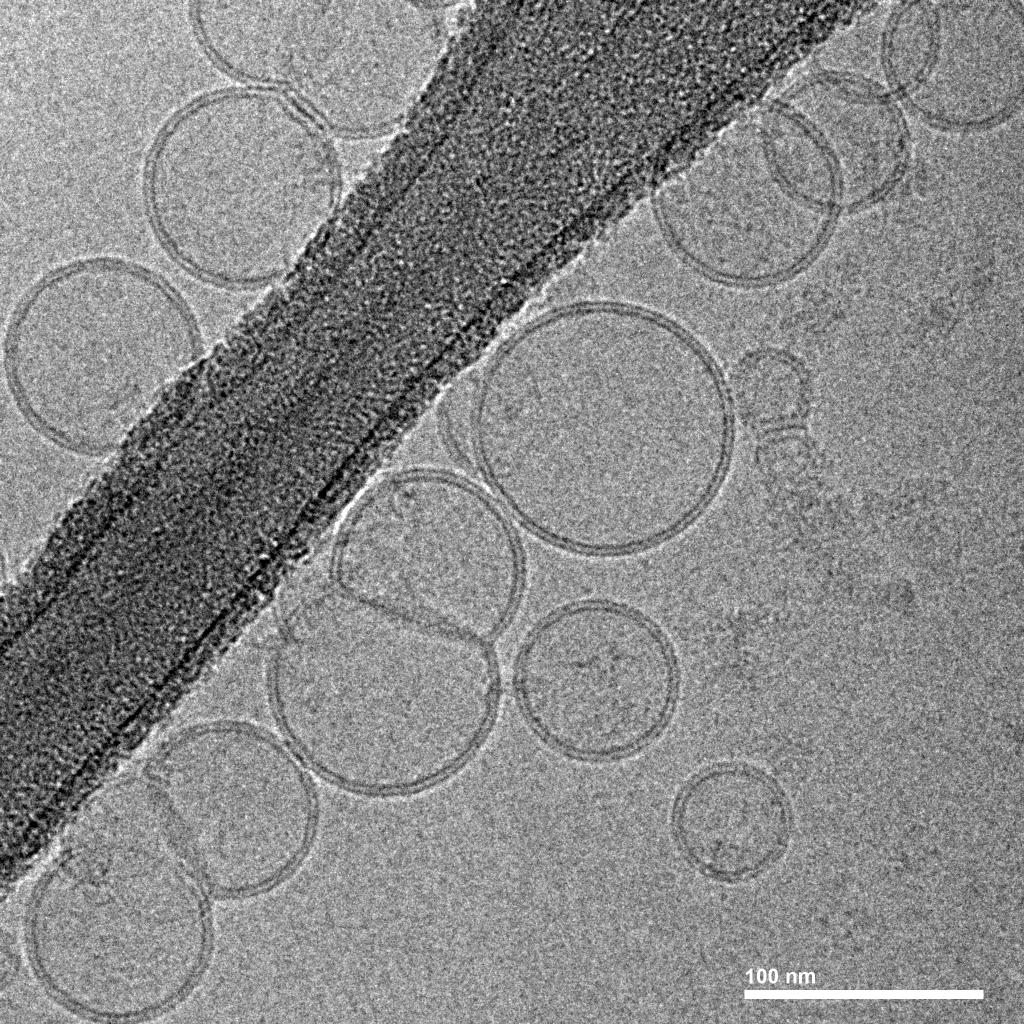

Supplement: Figure 2—source data 1. — This zip archive contains all cryo-EM images used for the quantitative analyses shown in Fig. 2. The folder named “No_Ca++” contains the images before Ca++ addition (individual files are named P3_1_**. tif or jpg), and folder named “With_Ca++” contains the images ∼35s after Ca++ addition (individual files are named P3_3_**.tif or jpg). Images were collected in low dose conditions at 200 kV acceleration voltage on a CM200 FEG electron microscope (FEI) with a 2k × 2k Gatan UltraScan 1000 camera, at 50,000× magnification and 1.5 mm underfocus. The full resolution data were exported as 16 bit “tif” files (2048 × 2048 pixels, scale 0.2 nm/pixel at specimen (the corresponding files have the extension “tif”). Note that these files cannot not be viewed with a standard picture viewer, but must be viewed with a program, such as “ImageJ”. To facilitate easier viewing, the original images were converted to smaller (1024×1024, 0.4 nm/pixel), contrast adjusted jpeg images (8 bits) for easy and immediate visualization with commonly used picture viewers (the corresponding files have the extension “jpg”). DOI: http://dx.doi.org/10.7554/eLife.00109.005 [file elife00109s001.zip › elife00109s001/With_Ca++/P3_3_07.jpg]

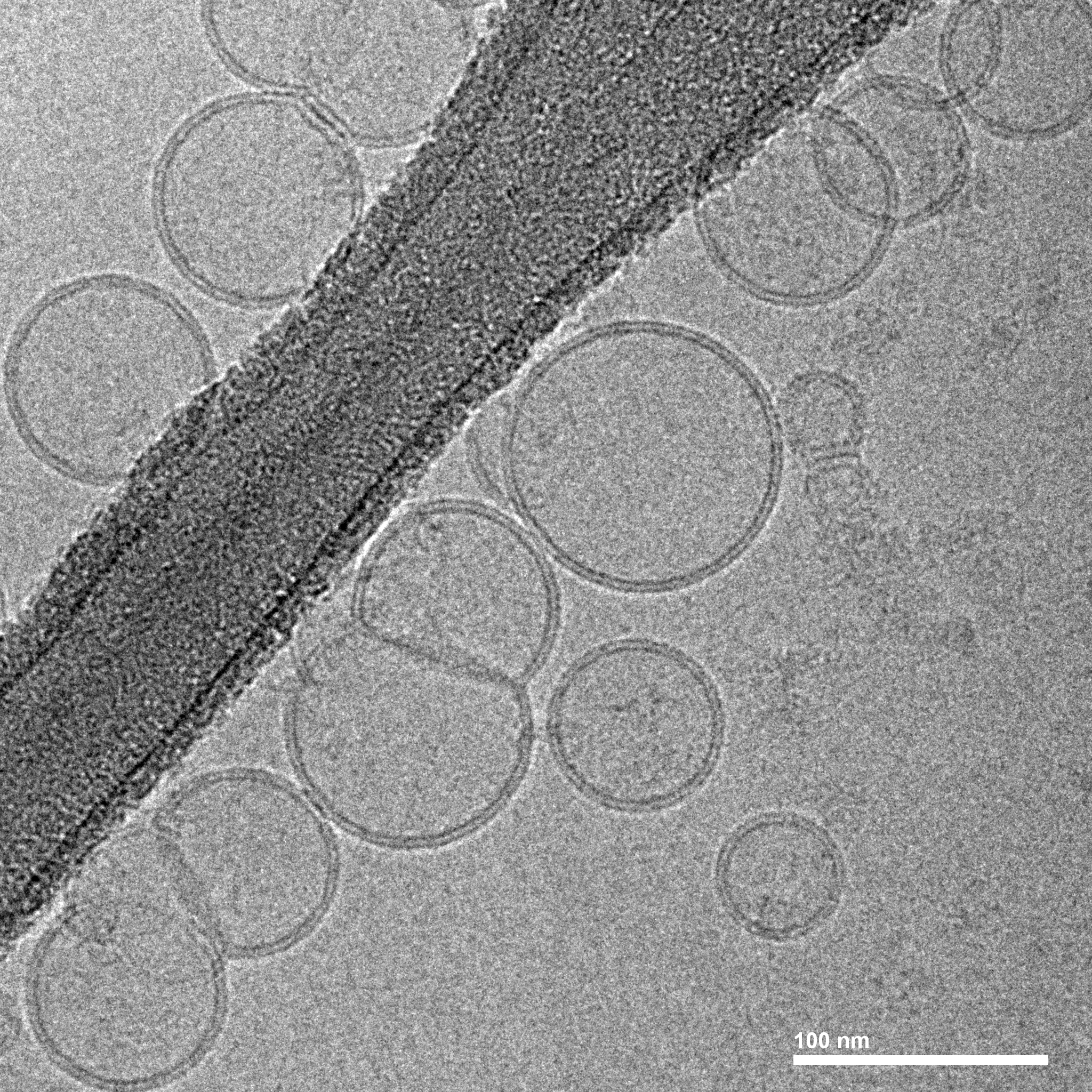

Supplement: Figure 2—source data 1. — This zip archive contains all cryo-EM images used for the quantitative analyses shown in Fig. 2. The folder named “No_Ca++” contains the images before Ca++ addition (individual files are named P3_1_**. tif or jpg), and folder named “With_Ca++” contains the images ∼35s after Ca++ addition (individual files are named P3_3_**.tif or jpg). Images were collected in low dose conditions at 200 kV acceleration voltage on a CM200 FEG electron microscope (FEI) with a 2k × 2k Gatan UltraScan 1000 camera, at 50,000× magnification and 1.5 mm underfocus. The full resolution data were exported as 16 bit “tif” files (2048 × 2048 pixels, scale 0.2 nm/pixel at specimen (the corresponding files have the extension “tif”). Note that these files cannot not be viewed with a standard picture viewer, but must be viewed with a program, such as “ImageJ”. To facilitate easier viewing, the original images were converted to smaller (1024×1024, 0.4 nm/pixel), contrast adjusted jpeg images (8 bits) for easy and immediate visualization with commonly used picture viewers (the corresponding files have the extension “jpg”). DOI: http://dx.doi.org/10.7554/eLife.00109.005 [file elife00109s001.zip › elife00109s001/With_Ca++/P3_3_07.tif]

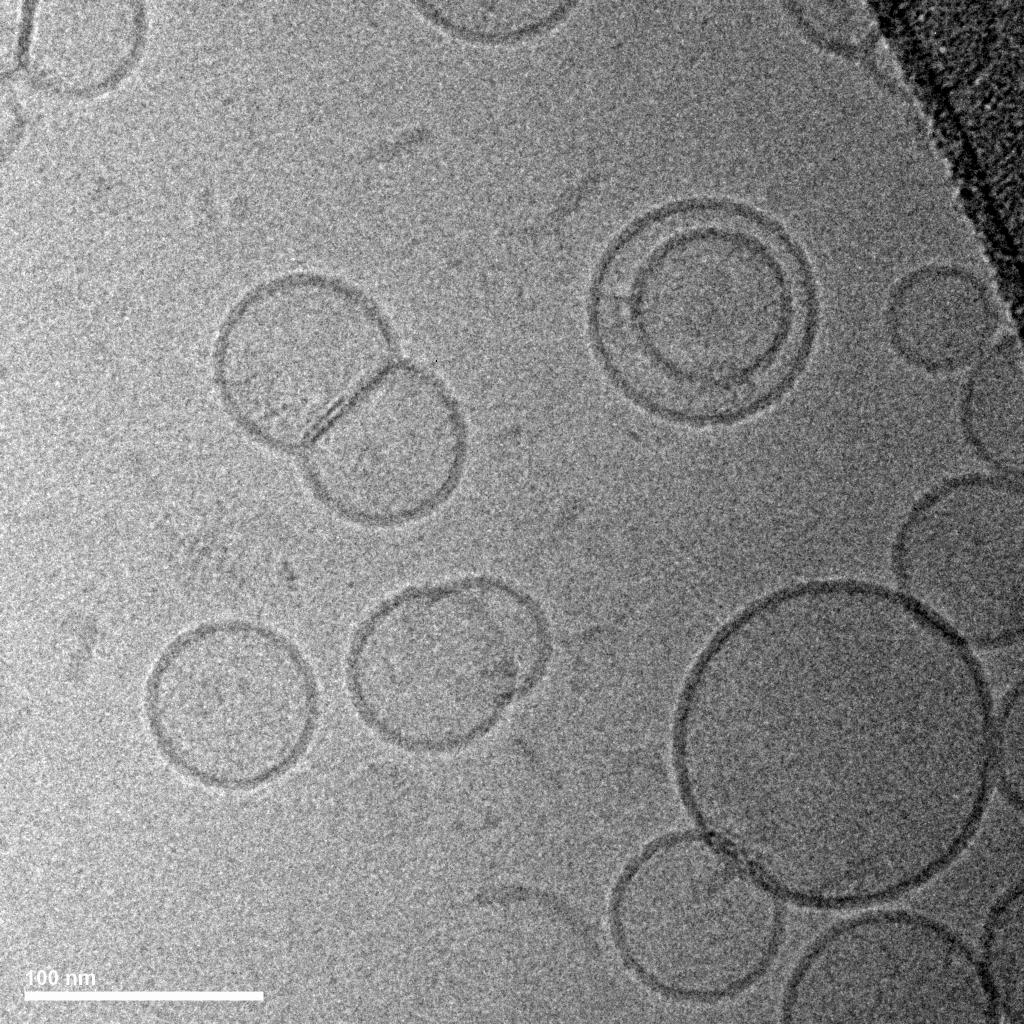

Supplement: Figure 2—source data 1. — This zip archive contains all cryo-EM images used for the quantitative analyses shown in Fig. 2. The folder named “No_Ca++” contains the images before Ca++ addition (individual files are named P3_1_**. tif or jpg), and folder named “With_Ca++” contains the images ∼35s after Ca++ addition (individual files are named P3_3_**.tif or jpg). Images were collected in low dose conditions at 200 kV acceleration voltage on a CM200 FEG electron microscope (FEI) with a 2k × 2k Gatan UltraScan 1000 camera, at 50,000× magnification and 1.5 mm underfocus. The full resolution data were exported as 16 bit “tif” files (2048 × 2048 pixels, scale 0.2 nm/pixel at specimen (the corresponding files have the extension “tif”). Note that these files cannot not be viewed with a standard picture viewer, but must be viewed with a program, such as “ImageJ”. To facilitate easier viewing, the original images were converted to smaller (1024×1024, 0.4 nm/pixel), contrast adjusted jpeg images (8 bits) for easy and immediate visualization with commonly used picture viewers (the corresponding files have the extension “jpg”). DOI: http://dx.doi.org/10.7554/eLife.00109.005 [file elife00109s001.zip › elife00109s001/With_Ca++/P3_3_09.jpg]

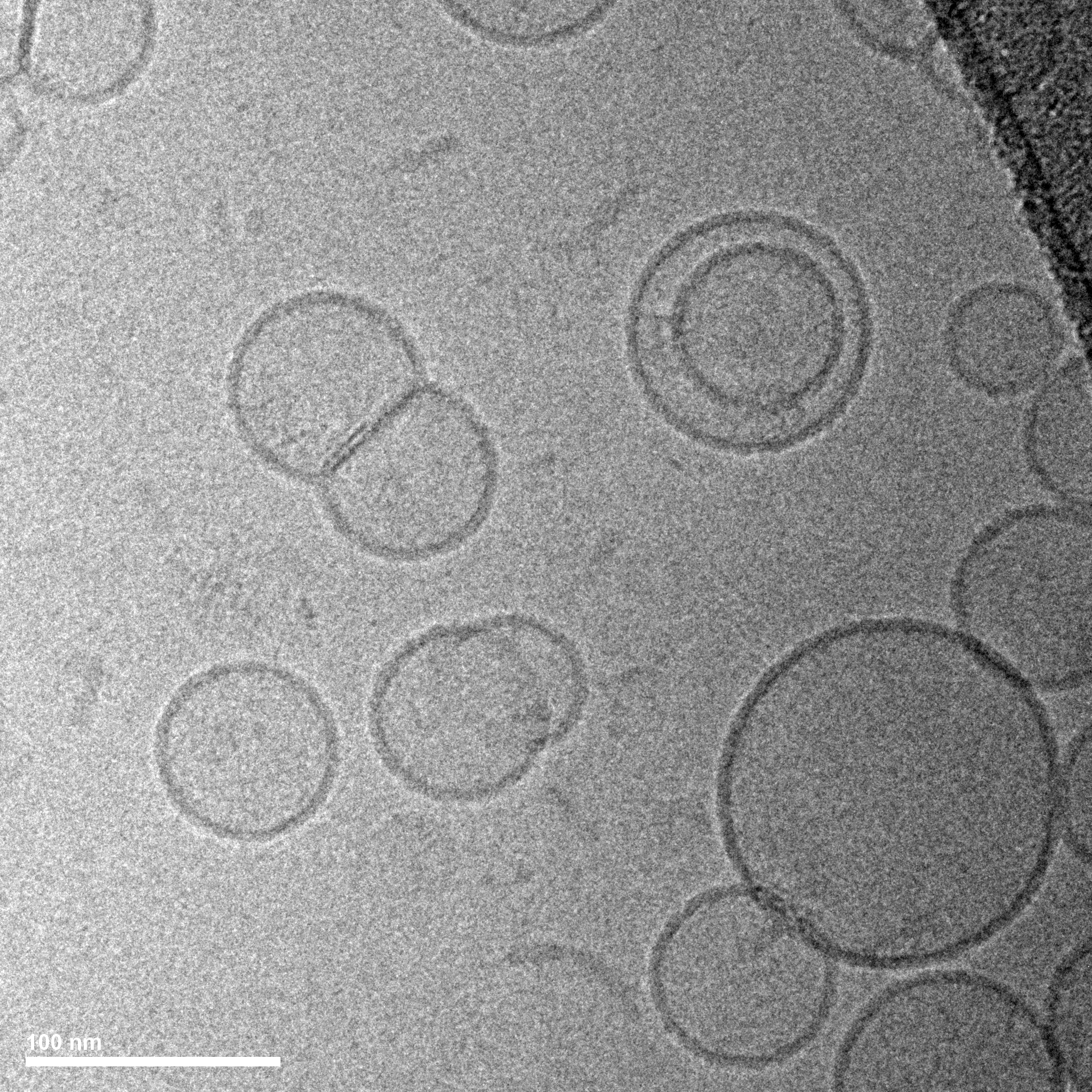

Supplement: Figure 2—source data 1. — This zip archive contains all cryo-EM images used for the quantitative analyses shown in Fig. 2. The folder named “No_Ca++” contains the images before Ca++ addition (individual files are named P3_1_**. tif or jpg), and folder named “With_Ca++” contains the images ∼35s after Ca++ addition (individual files are named P3_3_**.tif or jpg). Images were collected in low dose conditions at 200 kV acceleration voltage on a CM200 FEG electron microscope (FEI) with a 2k × 2k Gatan UltraScan 1000 camera, at 50,000× magnification and 1.5 mm underfocus. The full resolution data were exported as 16 bit “tif” files (2048 × 2048 pixels, scale 0.2 nm/pixel at specimen (the corresponding files have the extension “tif”). Note that these files cannot not be viewed with a standard picture viewer, but must be viewed with a program, such as “ImageJ”. To facilitate easier viewing, the original images were converted to smaller (1024×1024, 0.4 nm/pixel), contrast adjusted jpeg images (8 bits) for easy and immediate visualization with commonly used picture viewers (the corresponding files have the extension “jpg”). DOI: http://dx.doi.org/10.7554/eLife.00109.005 [file elife00109s001.zip › elife00109s001/With_Ca++/P3_3_09.tif]

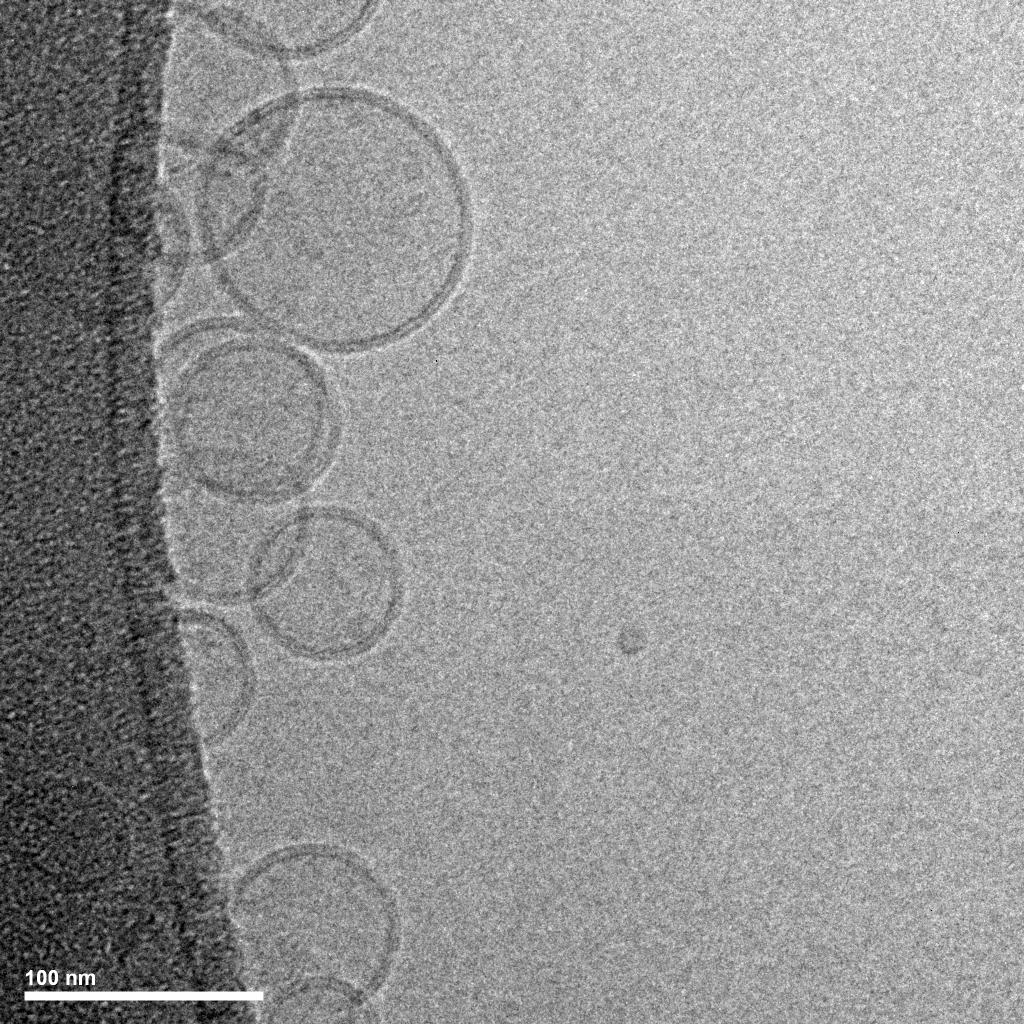

Supplement: Figure 2—source data 1. — This zip archive contains all cryo-EM images used for the quantitative analyses shown in Fig. 2. The folder named “No_Ca++” contains the images before Ca++ addition (individual files are named P3_1_**. tif or jpg), and folder named “With_Ca++” contains the images ∼35s after Ca++ addition (individual files are named P3_3_**.tif or jpg). Images were collected in low dose conditions at 200 kV acceleration voltage on a CM200 FEG electron microscope (FEI) with a 2k × 2k Gatan UltraScan 1000 camera, at 50,000× magnification and 1.5 mm underfocus. The full resolution data were exported as 16 bit “tif” files (2048 × 2048 pixels, scale 0.2 nm/pixel at specimen (the corresponding files have the extension “tif”). Note that these files cannot not be viewed with a standard picture viewer, but must be viewed with a program, such as “ImageJ”. To facilitate easier viewing, the original images were converted to smaller (1024×1024, 0.4 nm/pixel), contrast adjusted jpeg images (8 bits) for easy and immediate visualization with commonly used picture viewers (the corresponding files have the extension “jpg”). DOI: http://dx.doi.org/10.7554/eLife.00109.005 [file elife00109s001.zip › elife00109s001/With_Ca++/P3_3_10.jpg]

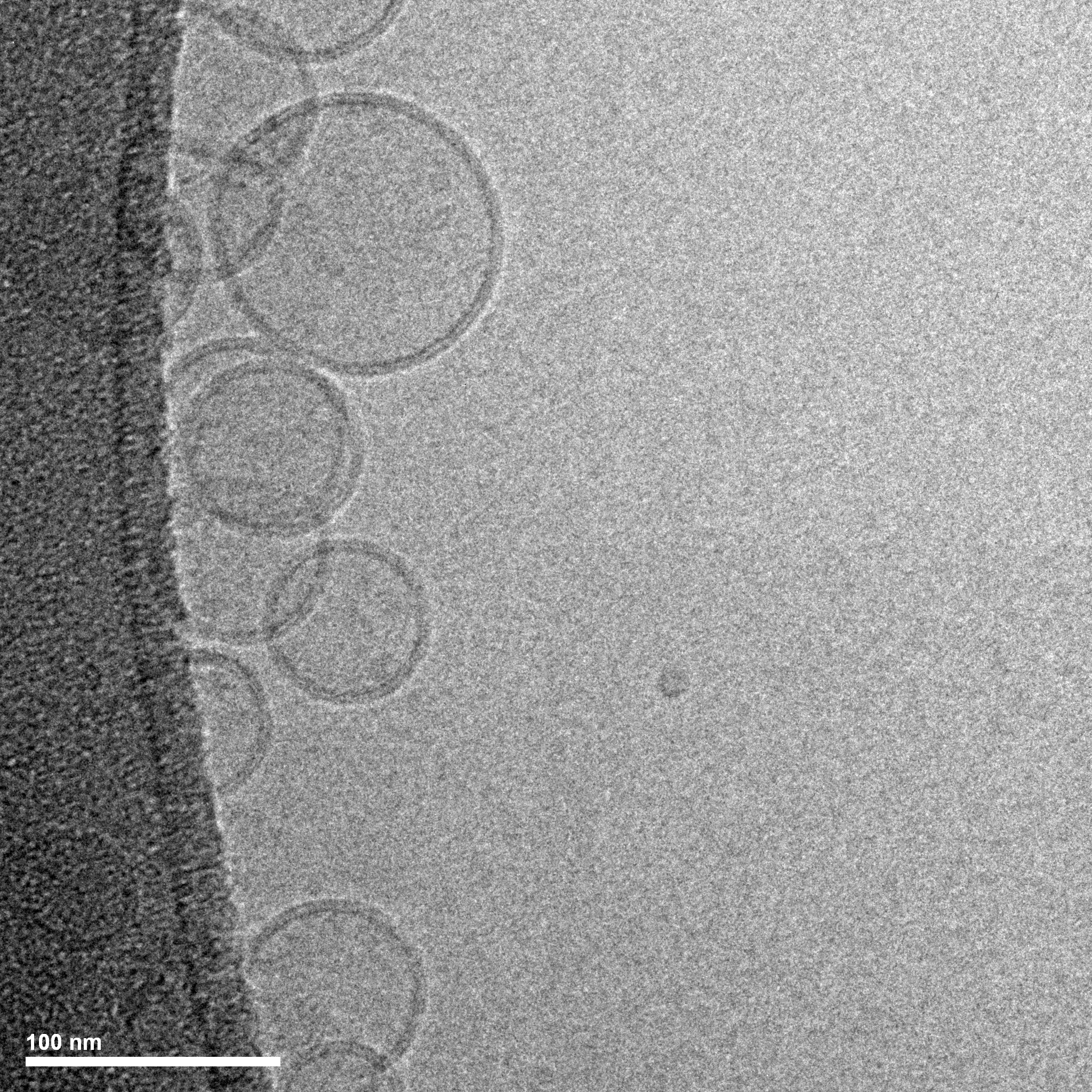

Supplement: Figure 2—source data 1. — This zip archive contains all cryo-EM images used for the quantitative analyses shown in Fig. 2. The folder named “No_Ca++” contains the images before Ca++ addition (individual files are named P3_1_**. tif or jpg), and folder named “With_Ca++” contains the images ∼35s after Ca++ addition (individual files are named P3_3_**.tif or jpg). Images were collected in low dose conditions at 200 kV acceleration voltage on a CM200 FEG electron microscope (FEI) with a 2k × 2k Gatan UltraScan 1000 camera, at 50,000× magnification and 1.5 mm underfocus. The full resolution data were exported as 16 bit “tif” files (2048 × 2048 pixels, scale 0.2 nm/pixel at specimen (the corresponding files have the extension “tif”). Note that these files cannot not be viewed with a standard picture viewer, but must be viewed with a program, such as “ImageJ”. To facilitate easier viewing, the original images were converted to smaller (1024×1024, 0.4 nm/pixel), contrast adjusted jpeg images (8 bits) for easy and immediate visualization with commonly used picture viewers (the corresponding files have the extension “jpg”). DOI: http://dx.doi.org/10.7554/eLife.00109.005 [file elife00109s001.zip › elife00109s001/With_Ca++/P3_3_10.tif]

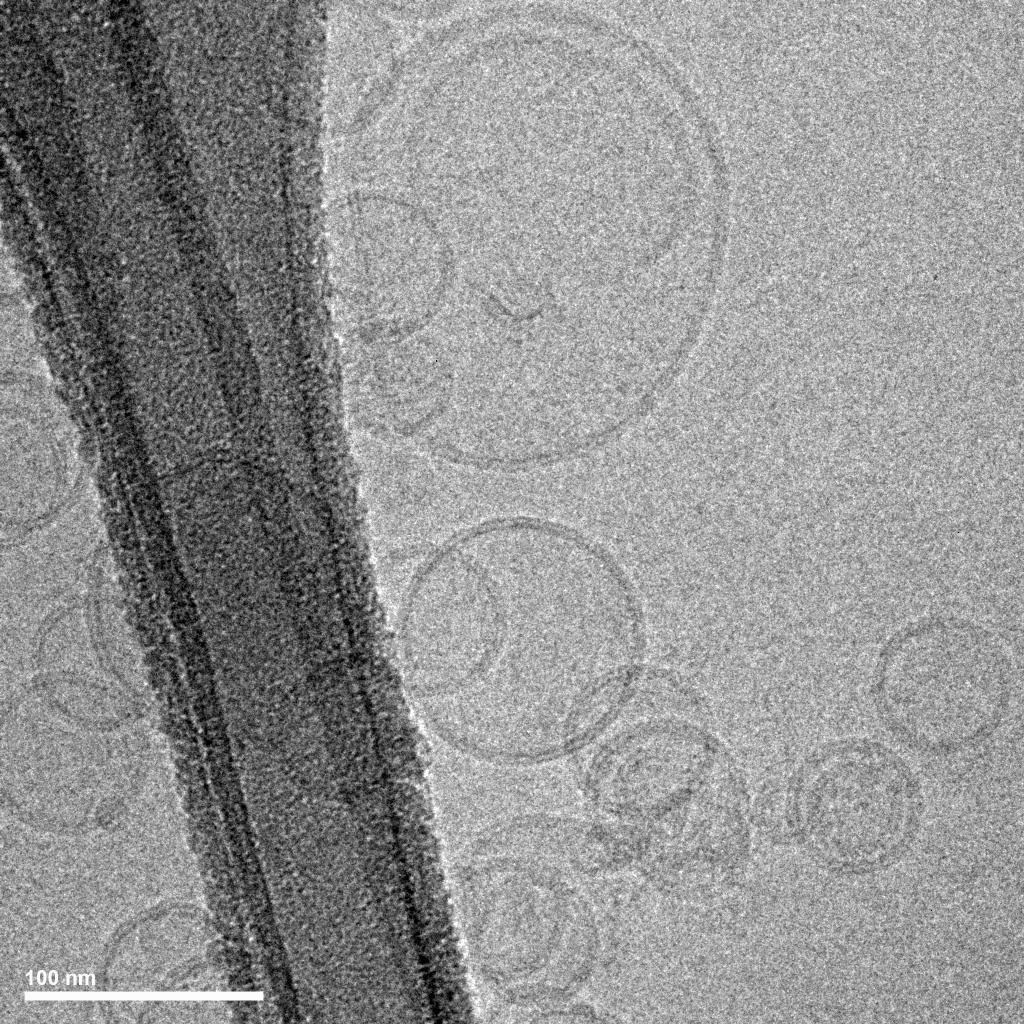

Supplement: Figure 2—source data 1. — This zip archive contains all cryo-EM images used for the quantitative analyses shown in Fig. 2. The folder named “No_Ca++” contains the images before Ca++ addition (individual files are named P3_1_**. tif or jpg), and folder named “With_Ca++” contains the images ∼35s after Ca++ addition (individual files are named P3_3_**.tif or jpg). Images were collected in low dose conditions at 200 kV acceleration voltage on a CM200 FEG electron microscope (FEI) with a 2k × 2k Gatan UltraScan 1000 camera, at 50,000× magnification and 1.5 mm underfocus. The full resolution data were exported as 16 bit “tif” files (2048 × 2048 pixels, scale 0.2 nm/pixel at specimen (the corresponding files have the extension “tif”). Note that these files cannot not be viewed with a standard picture viewer, but must be viewed with a program, such as “ImageJ”. To facilitate easier viewing, the original images were converted to smaller (1024×1024, 0.4 nm/pixel), contrast adjusted jpeg images (8 bits) for easy and immediate visualization with commonly used picture viewers (the corresponding files have the extension “jpg”). DOI: http://dx.doi.org/10.7554/eLife.00109.005 [file elife00109s001.zip › elife00109s001/With_Ca++/P3_3_11.jpg]

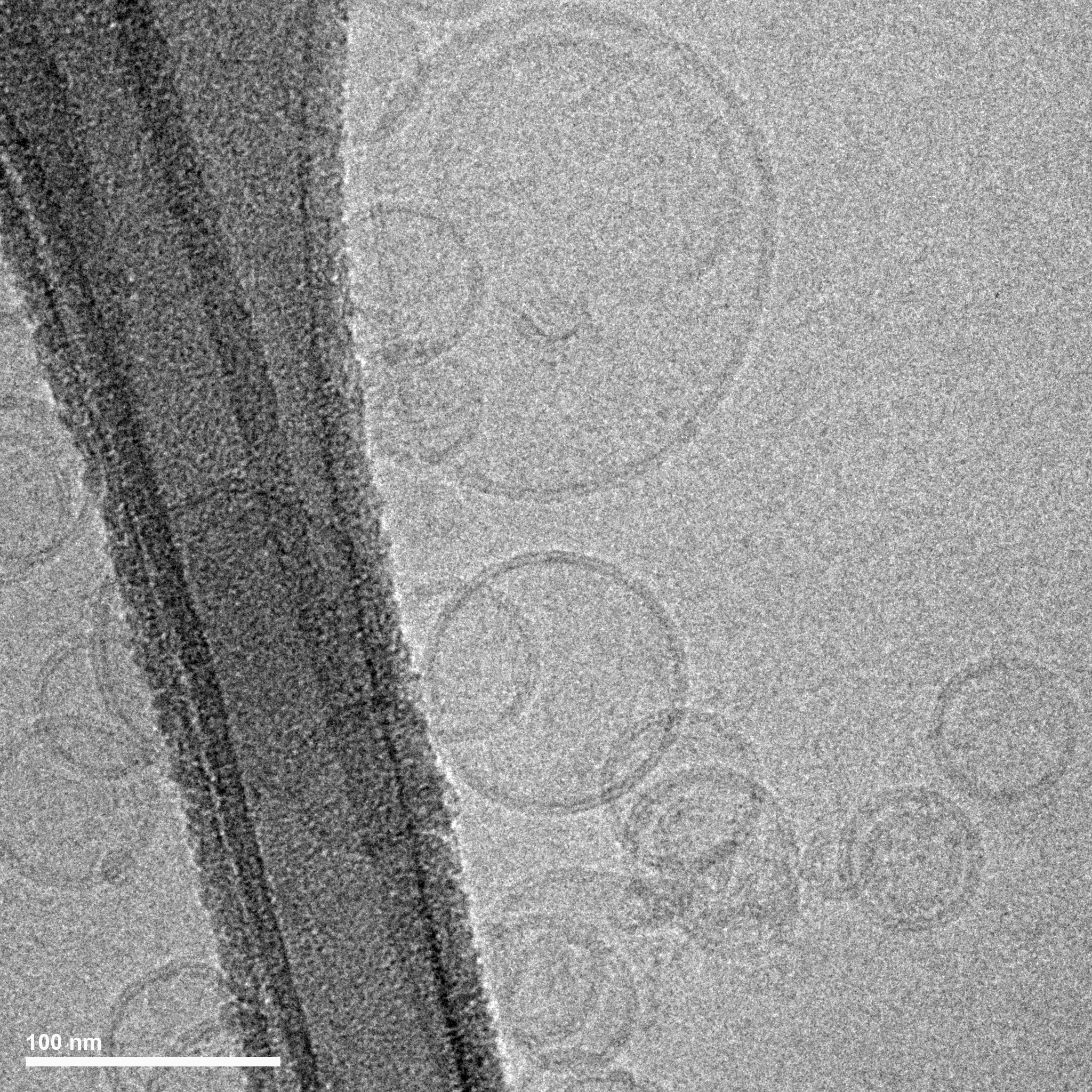

Supplement: Figure 2—source data 1. — This zip archive contains all cryo-EM images used for the quantitative analyses shown in Fig. 2. The folder named “No_Ca++” contains the images before Ca++ addition (individual files are named P3_1_**. tif or jpg), and folder named “With_Ca++” contains the images ∼35s after Ca++ addition (individual files are named P3_3_**.tif or jpg). Images were collected in low dose conditions at 200 kV acceleration voltage on a CM200 FEG electron microscope (FEI) with a 2k × 2k Gatan UltraScan 1000 camera, at 50,000× magnification and 1.5 mm underfocus. The full resolution data were exported as 16 bit “tif” files (2048 × 2048 pixels, scale 0.2 nm/pixel at specimen (the corresponding files have the extension “tif”). Note that these files cannot not be viewed with a standard picture viewer, but must be viewed with a program, such as “ImageJ”. To facilitate easier viewing, the original images were converted to smaller (1024×1024, 0.4 nm/pixel), contrast adjusted jpeg images (8 bits) for easy and immediate visualization with commonly used picture viewers (the corresponding files have the extension “jpg”). DOI: http://dx.doi.org/10.7554/eLife.00109.005 [file elife00109s001.zip › elife00109s001/With_Ca++/P3_3_11.tif]

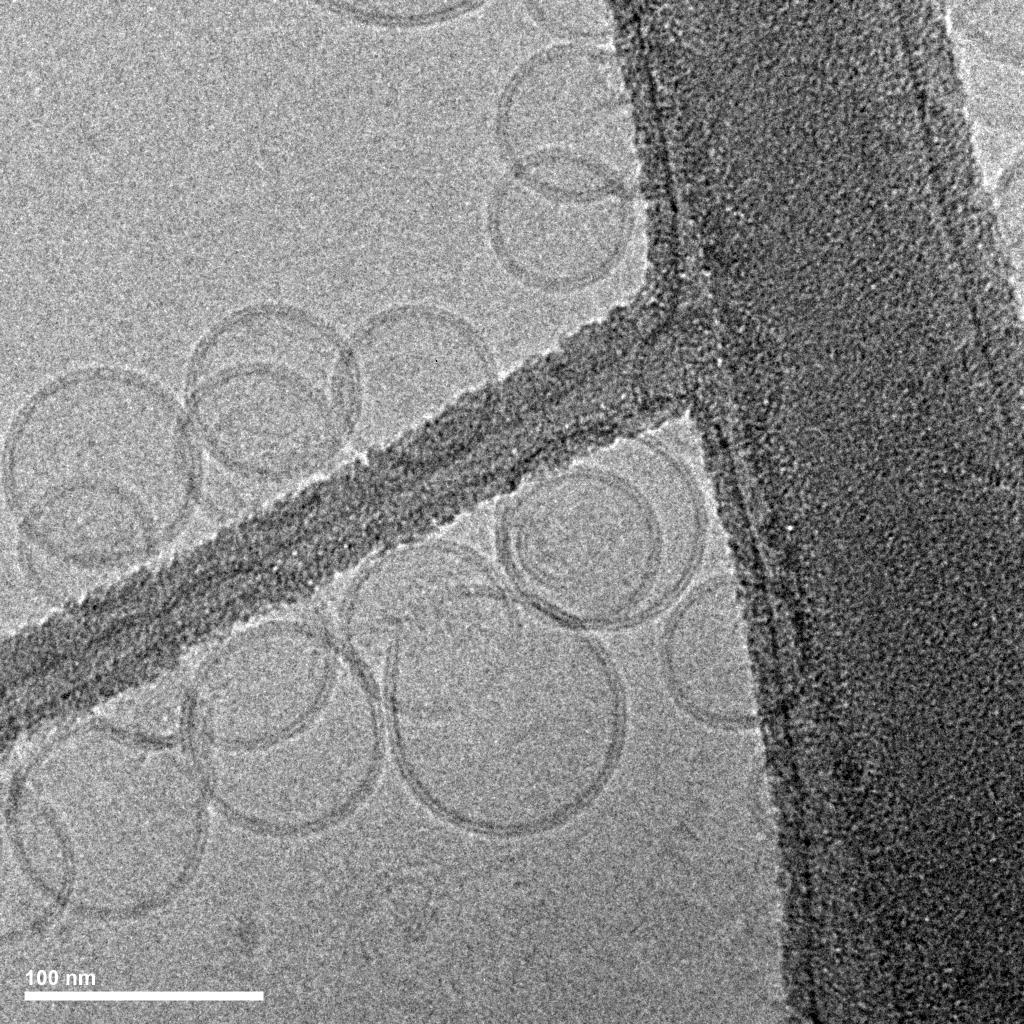

Supplement: Figure 2—source data 1. — This zip archive contains all cryo-EM images used for the quantitative analyses shown in Fig. 2. The folder named “No_Ca++” contains the images before Ca++ addition (individual files are named P3_1_**. tif or jpg), and folder named “With_Ca++” contains the images ∼35s after Ca++ addition (individual files are named P3_3_**.tif or jpg). Images were collected in low dose conditions at 200 kV acceleration voltage on a CM200 FEG electron microscope (FEI) with a 2k × 2k Gatan UltraScan 1000 camera, at 50,000× magnification and 1.5 mm underfocus. The full resolution data were exported as 16 bit “tif” files (2048 × 2048 pixels, scale 0.2 nm/pixel at specimen (the corresponding files have the extension “tif”). Note that these files cannot not be viewed with a standard picture viewer, but must be viewed with a program, such as “ImageJ”. To facilitate easier viewing, the original images were converted to smaller (1024×1024, 0.4 nm/pixel), contrast adjusted jpeg images (8 bits) for easy and immediate visualization with commonly used picture viewers (the corresponding files have the extension “jpg”). DOI: http://dx.doi.org/10.7554/eLife.00109.005 [file elife00109s001.zip › elife00109s001/With_Ca++/P3_3_14.jpg]

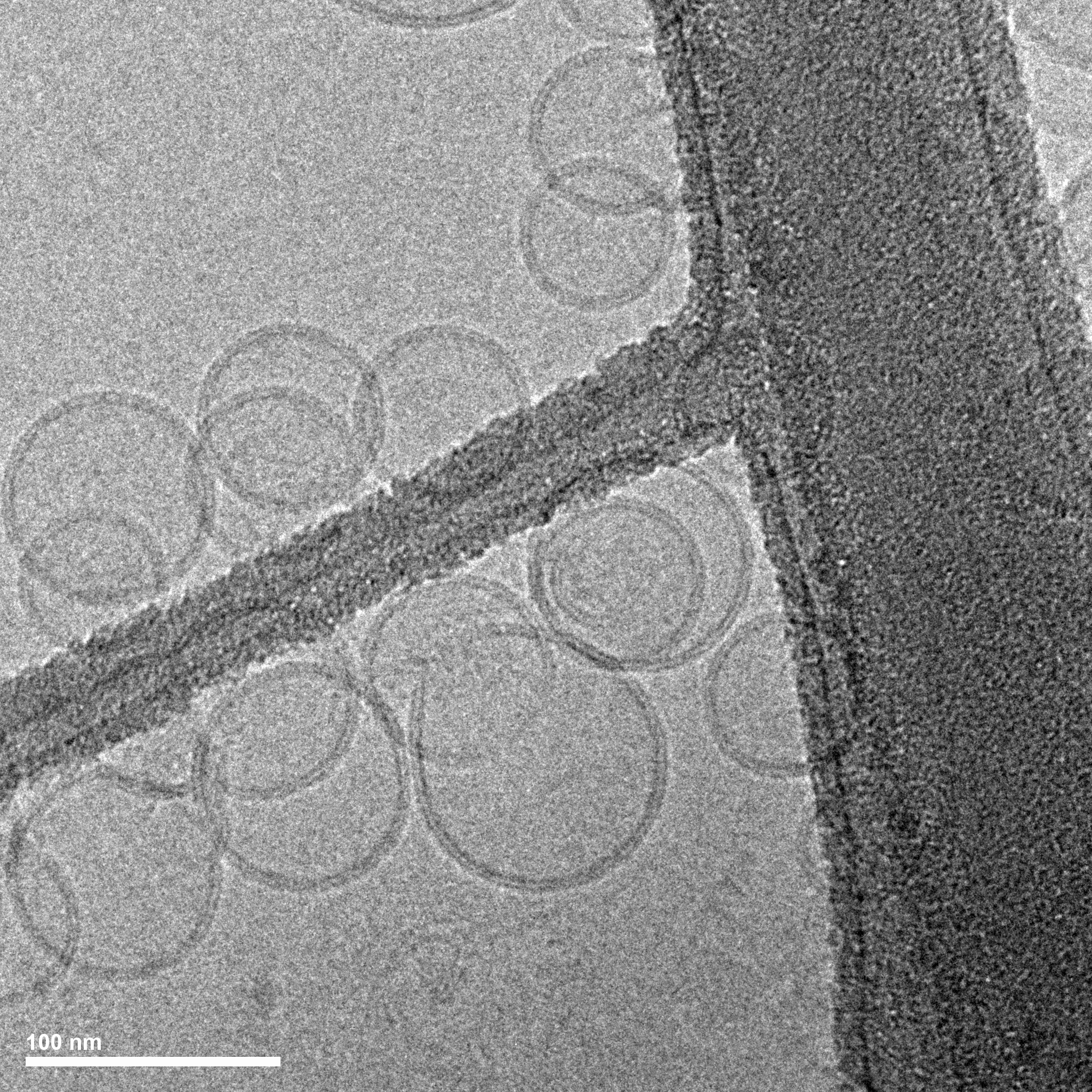

Supplement: Figure 2—source data 1. — This zip archive contains all cryo-EM images used for the quantitative analyses shown in Fig. 2. The folder named “No_Ca++” contains the images before Ca++ addition (individual files are named P3_1_**. tif or jpg), and folder named “With_Ca++” contains the images ∼35s after Ca++ addition (individual files are named P3_3_**.tif or jpg). Images were collected in low dose conditions at 200 kV acceleration voltage on a CM200 FEG electron microscope (FEI) with a 2k × 2k Gatan UltraScan 1000 camera, at 50,000× magnification and 1.5 mm underfocus. The full resolution data were exported as 16 bit “tif” files (2048 × 2048 pixels, scale 0.2 nm/pixel at specimen (the corresponding files have the extension “tif”). Note that these files cannot not be viewed with a standard picture viewer, but must be viewed with a program, such as “ImageJ”. To facilitate easier viewing, the original images were converted to smaller (1024×1024, 0.4 nm/pixel), contrast adjusted jpeg images (8 bits) for easy and immediate visualization with commonly used picture viewers (the corresponding files have the extension “jpg”). DOI: http://dx.doi.org/10.7554/eLife.00109.005 [file elife00109s001.zip › elife00109s001/With_Ca++/P3_3_14.tif]

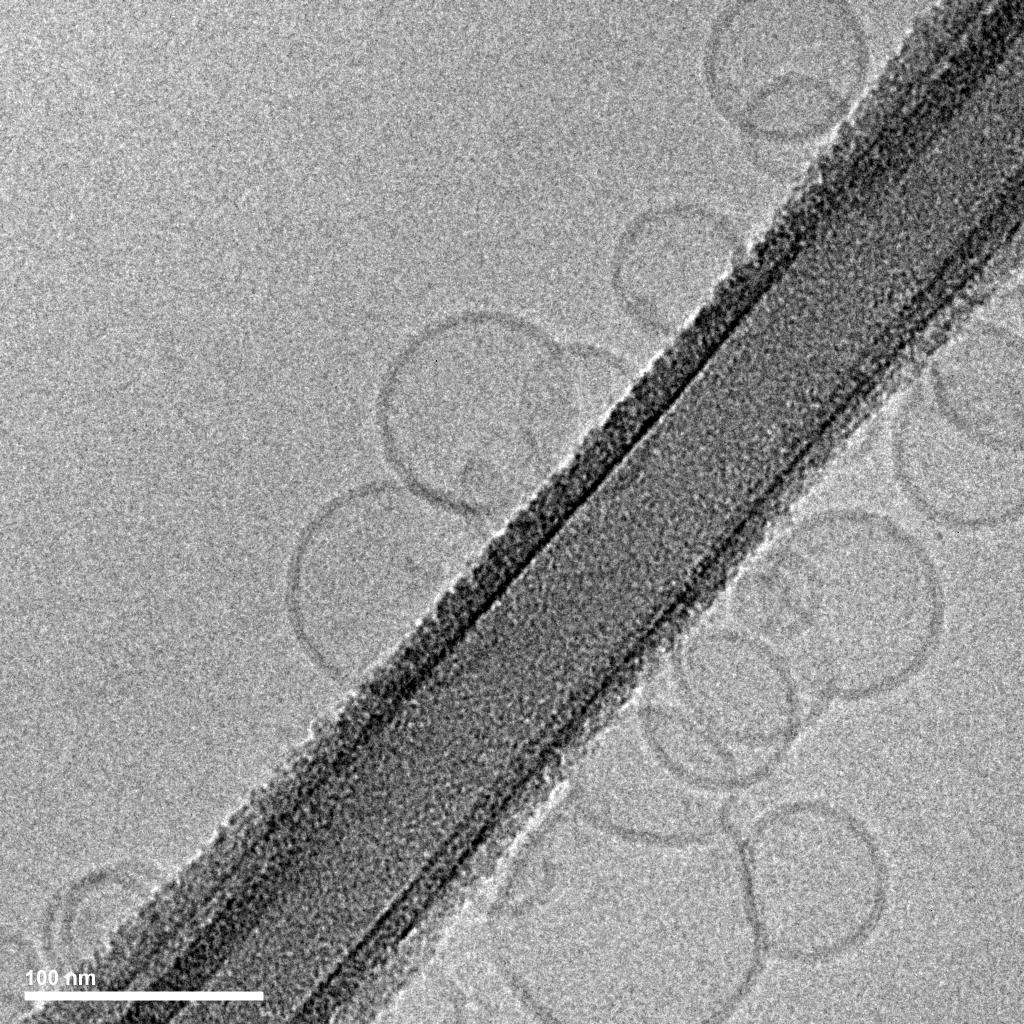

Supplement: Figure 2—source data 1. — This zip archive contains all cryo-EM images used for the quantitative analyses shown in Fig. 2. The folder named “No_Ca++” contains the images before Ca++ addition (individual files are named P3_1_**. tif or jpg), and folder named “With_Ca++” contains the images ∼35s after Ca++ addition (individual files are named P3_3_**.tif or jpg). Images were collected in low dose conditions at 200 kV acceleration voltage on a CM200 FEG electron microscope (FEI) with a 2k × 2k Gatan UltraScan 1000 camera, at 50,000× magnification and 1.5 mm underfocus. The full resolution data were exported as 16 bit “tif” files (2048 × 2048 pixels, scale 0.2 nm/pixel at specimen (the corresponding files have the extension “tif”). Note that these files cannot not be viewed with a standard picture viewer, but must be viewed with a program, such as “ImageJ”. To facilitate easier viewing, the original images were converted to smaller (1024×1024, 0.4 nm/pixel), contrast adjusted jpeg images (8 bits) for easy and immediate visualization with commonly used picture viewers (the corresponding files have the extension “jpg”). DOI: http://dx.doi.org/10.7554/eLife.00109.005 [file elife00109s001.zip › elife00109s001/With_Ca++/P3_3_15.jpg]

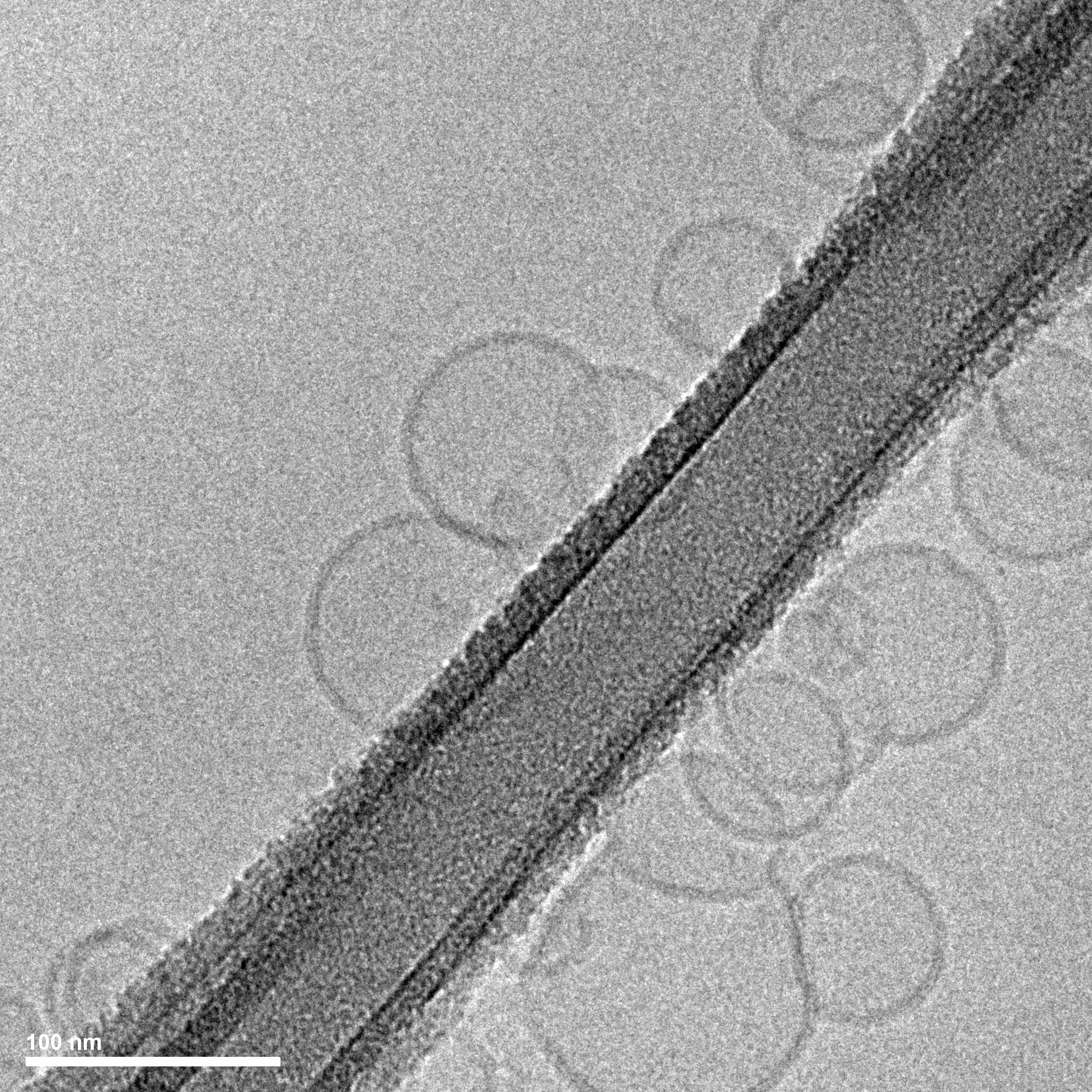

Supplement: Figure 2—source data 1. — This zip archive contains all cryo-EM images used for the quantitative analyses shown in Fig. 2. The folder named “No_Ca++” contains the images before Ca++ addition (individual files are named P3_1_**. tif or jpg), and folder named “With_Ca++” contains the images ∼35s after Ca++ addition (individual files are named P3_3_**.tif or jpg). Images were collected in low dose conditions at 200 kV acceleration voltage on a CM200 FEG electron microscope (FEI) with a 2k × 2k Gatan UltraScan 1000 camera, at 50,000× magnification and 1.5 mm underfocus. The full resolution data were exported as 16 bit “tif” files (2048 × 2048 pixels, scale 0.2 nm/pixel at specimen (the corresponding files have the extension “tif”). Note that these files cannot not be viewed with a standard picture viewer, but must be viewed with a program, such as “ImageJ”. To facilitate easier viewing, the original images were converted to smaller (1024×1024, 0.4 nm/pixel), contrast adjusted jpeg images (8 bits) for easy and immediate visualization with commonly used picture viewers (the corresponding files have the extension “jpg”). DOI: http://dx.doi.org/10.7554/eLife.00109.005 [file elife00109s001.zip › elife00109s001/With_Ca++/P3_3_15.tif]

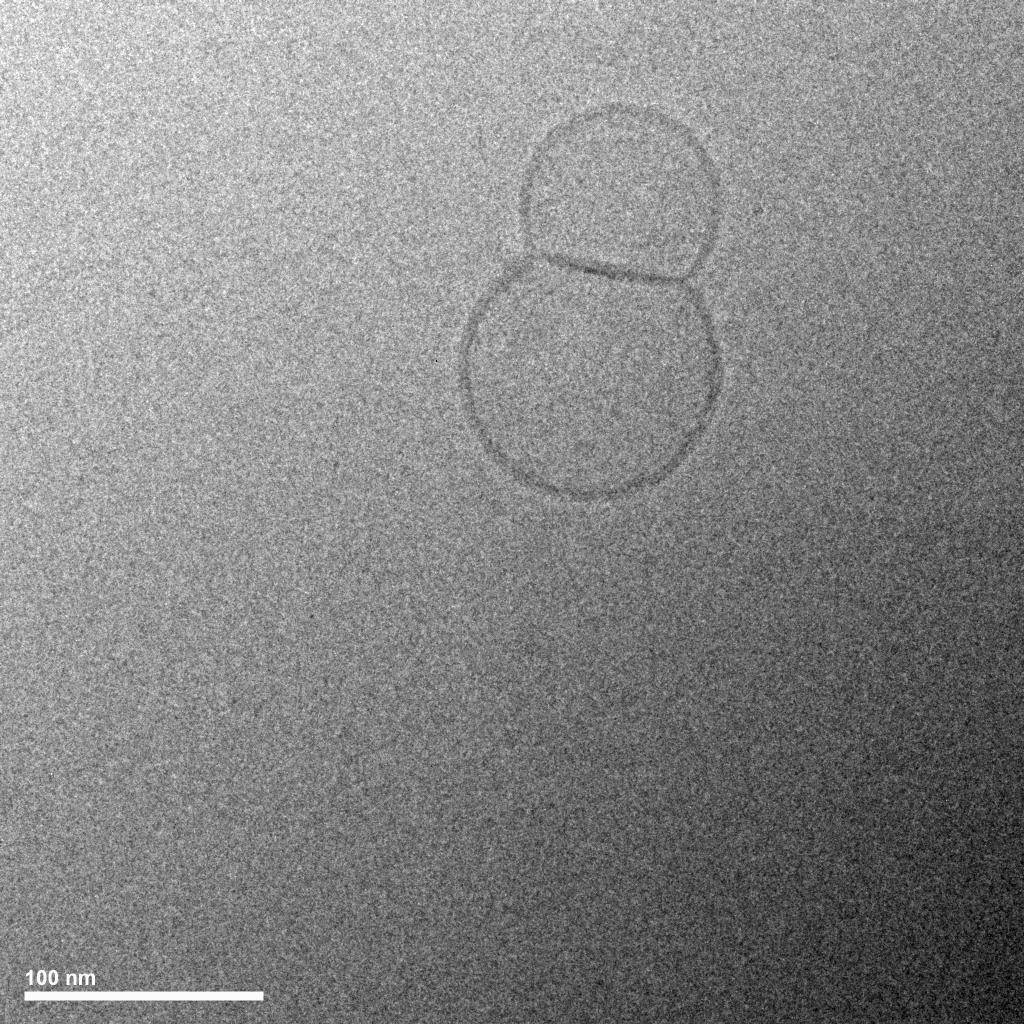

Supplement: Figure 2—source data 1. — This zip archive contains all cryo-EM images used for the quantitative analyses shown in Fig. 2. The folder named “No_Ca++” contains the images before Ca++ addition (individual files are named P3_1_**. tif or jpg), and folder named “With_Ca++” contains the images ∼35s after Ca++ addition (individual files are named P3_3_**.tif or jpg). Images were collected in low dose conditions at 200 kV acceleration voltage on a CM200 FEG electron microscope (FEI) with a 2k × 2k Gatan UltraScan 1000 camera, at 50,000× magnification and 1.5 mm underfocus. The full resolution data were exported as 16 bit “tif” files (2048 × 2048 pixels, scale 0.2 nm/pixel at specimen (the corresponding files have the extension “tif”). Note that these files cannot not be viewed with a standard picture viewer, but must be viewed with a program, such as “ImageJ”. To facilitate easier viewing, the original images were converted to smaller (1024×1024, 0.4 nm/pixel), contrast adjusted jpeg images (8 bits) for easy and immediate visualization with commonly used picture viewers (the corresponding files have the extension “jpg”). DOI: http://dx.doi.org/10.7554/eLife.00109.005 [file elife00109s001.zip › elife00109s001/With_Ca++/P3_3_16.jpg]

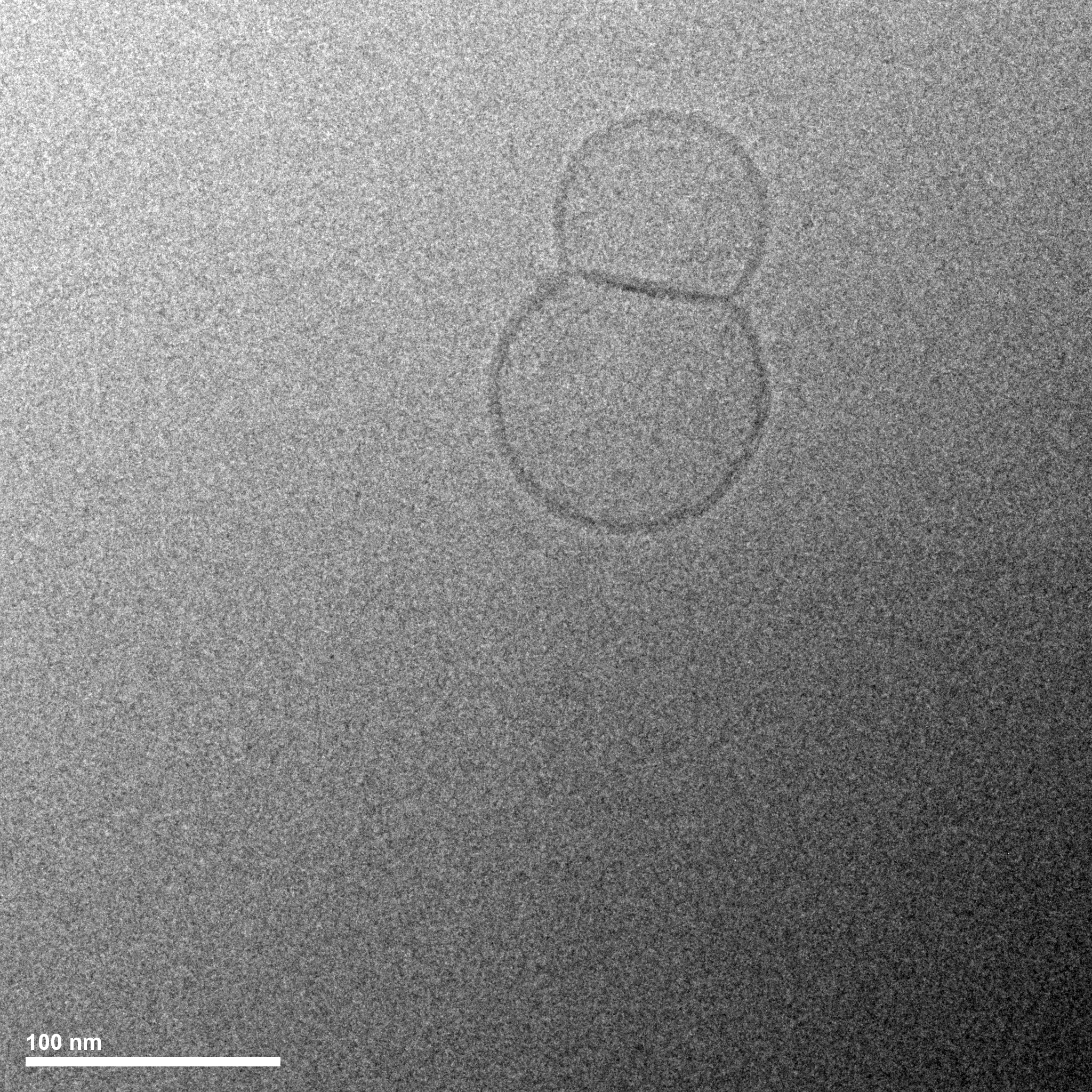

Supplement: Figure 2—source data 1. — This zip archive contains all cryo-EM images used for the quantitative analyses shown in Fig. 2. The folder named “No_Ca++” contains the images before Ca++ addition (individual files are named P3_1_**. tif or jpg), and folder named “With_Ca++” contains the images ∼35s after Ca++ addition (individual files are named P3_3_**.tif or jpg). Images were collected in low dose conditions at 200 kV acceleration voltage on a CM200 FEG electron microscope (FEI) with a 2k × 2k Gatan UltraScan 1000 camera, at 50,000× magnification and 1.5 mm underfocus. The full resolution data were exported as 16 bit “tif” files (2048 × 2048 pixels, scale 0.2 nm/pixel at specimen (the corresponding files have the extension “tif”). Note that these files cannot not be viewed with a standard picture viewer, but must be viewed with a program, such as “ImageJ”. To facilitate easier viewing, the original images were converted to smaller (1024×1024, 0.4 nm/pixel), contrast adjusted jpeg images (8 bits) for easy and immediate visualization with commonly used picture viewers (the corresponding files have the extension “jpg”). DOI: http://dx.doi.org/10.7554/eLife.00109.005 [file elife00109s001.zip › elife00109s001/With_Ca++/P3_3_16.tif]

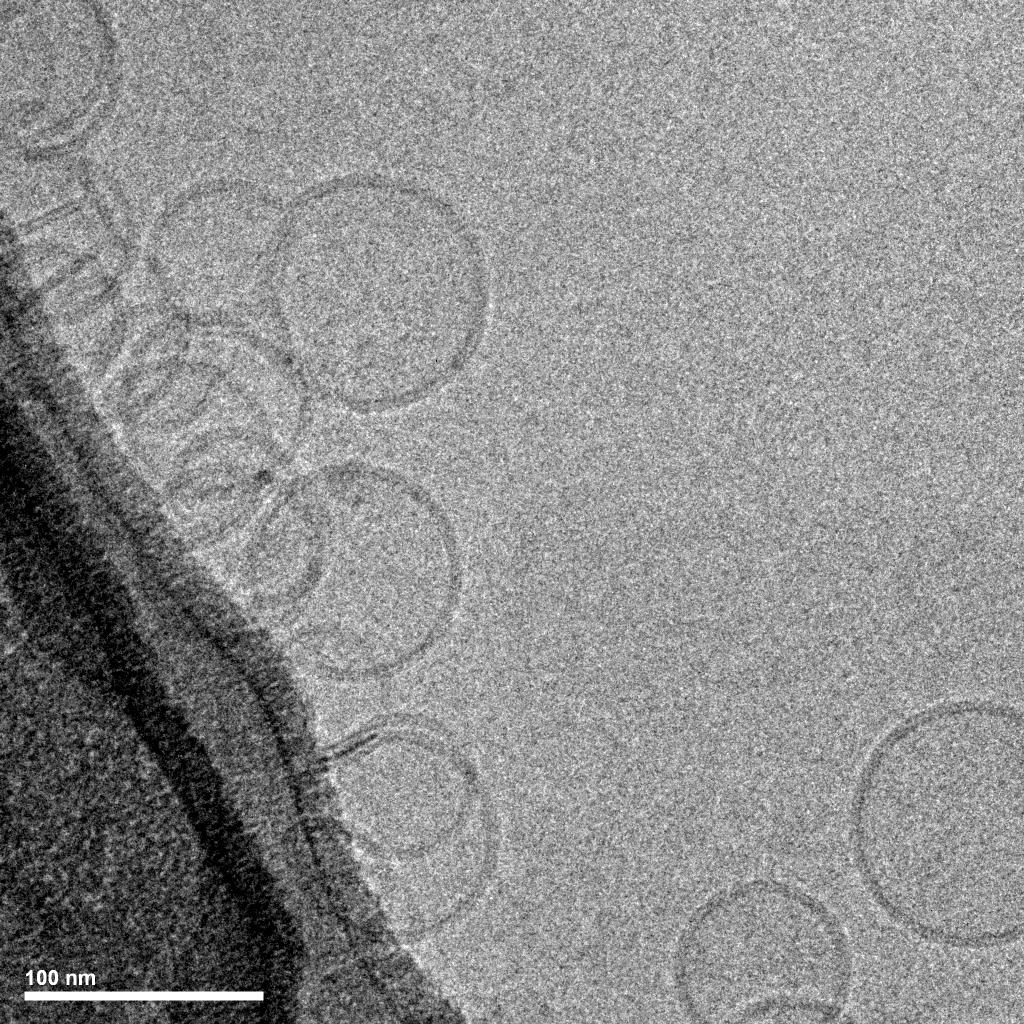

Supplement: Figure 2—source data 1. — This zip archive contains all cryo-EM images used for the quantitative analyses shown in Fig. 2. The folder named “No_Ca++” contains the images before Ca++ addition (individual files are named P3_1_**. tif or jpg), and folder named “With_Ca++” contains the images ∼35s after Ca++ addition (individual files are named P3_3_**.tif or jpg). Images were collected in low dose conditions at 200 kV acceleration voltage on a CM200 FEG electron microscope (FEI) with a 2k × 2k Gatan UltraScan 1000 camera, at 50,000× magnification and 1.5 mm underfocus. The full resolution data were exported as 16 bit “tif” files (2048 × 2048 pixels, scale 0.2 nm/pixel at specimen (the corresponding files have the extension “tif”). Note that these files cannot not be viewed with a standard picture viewer, but must be viewed with a program, such as “ImageJ”. To facilitate easier viewing, the original images were converted to smaller (1024×1024, 0.4 nm/pixel), contrast adjusted jpeg images (8 bits) for easy and immediate visualization with commonly used picture viewers (the corresponding files have the extension “jpg”). DOI: http://dx.doi.org/10.7554/eLife.00109.005 [file elife00109s001.zip › elife00109s001/With_Ca++/P3_3_18.jpg]

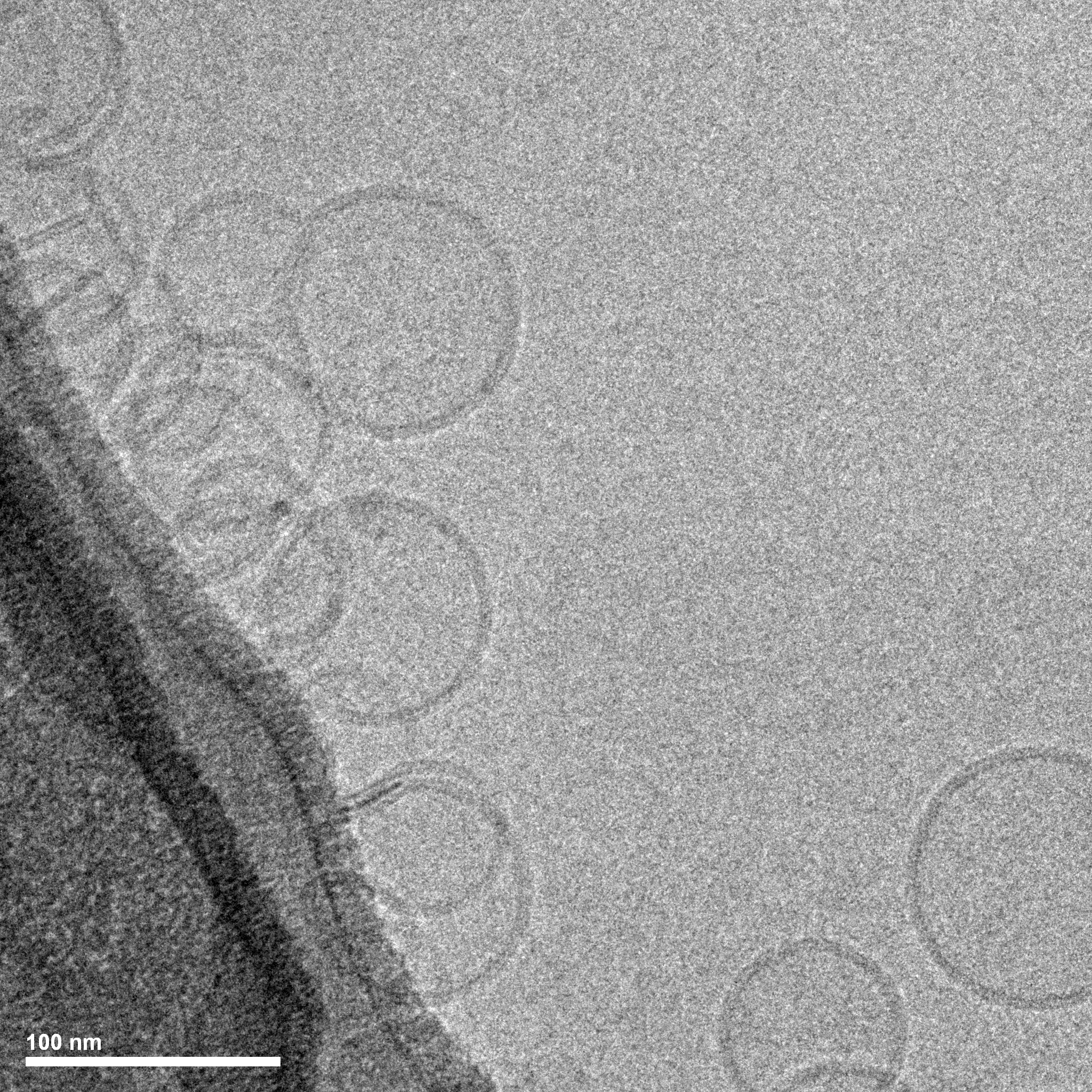

Supplement: Figure 2—source data 1. — This zip archive contains all cryo-EM images used for the quantitative analyses shown in Fig. 2. The folder named “No_Ca++” contains the images before Ca++ addition (individual files are named P3_1_**. tif or jpg), and folder named “With_Ca++” contains the images ∼35s after Ca++ addition (individual files are named P3_3_**.tif or jpg). Images were collected in low dose conditions at 200 kV acceleration voltage on a CM200 FEG electron microscope (FEI) with a 2k × 2k Gatan UltraScan 1000 camera, at 50,000× magnification and 1.5 mm underfocus. The full resolution data were exported as 16 bit “tif” files (2048 × 2048 pixels, scale 0.2 nm/pixel at specimen (the corresponding files have the extension “tif”). Note that these files cannot not be viewed with a standard picture viewer, but must be viewed with a program, such as “ImageJ”. To facilitate easier viewing, the original images were converted to smaller (1024×1024, 0.4 nm/pixel), contrast adjusted jpeg images (8 bits) for easy and immediate visualization with commonly used picture viewers (the corresponding files have the extension “jpg”). DOI: http://dx.doi.org/10.7554/eLife.00109.005 [file elife00109s001.zip › elife00109s001/With_Ca++/P3_3_18.tif]

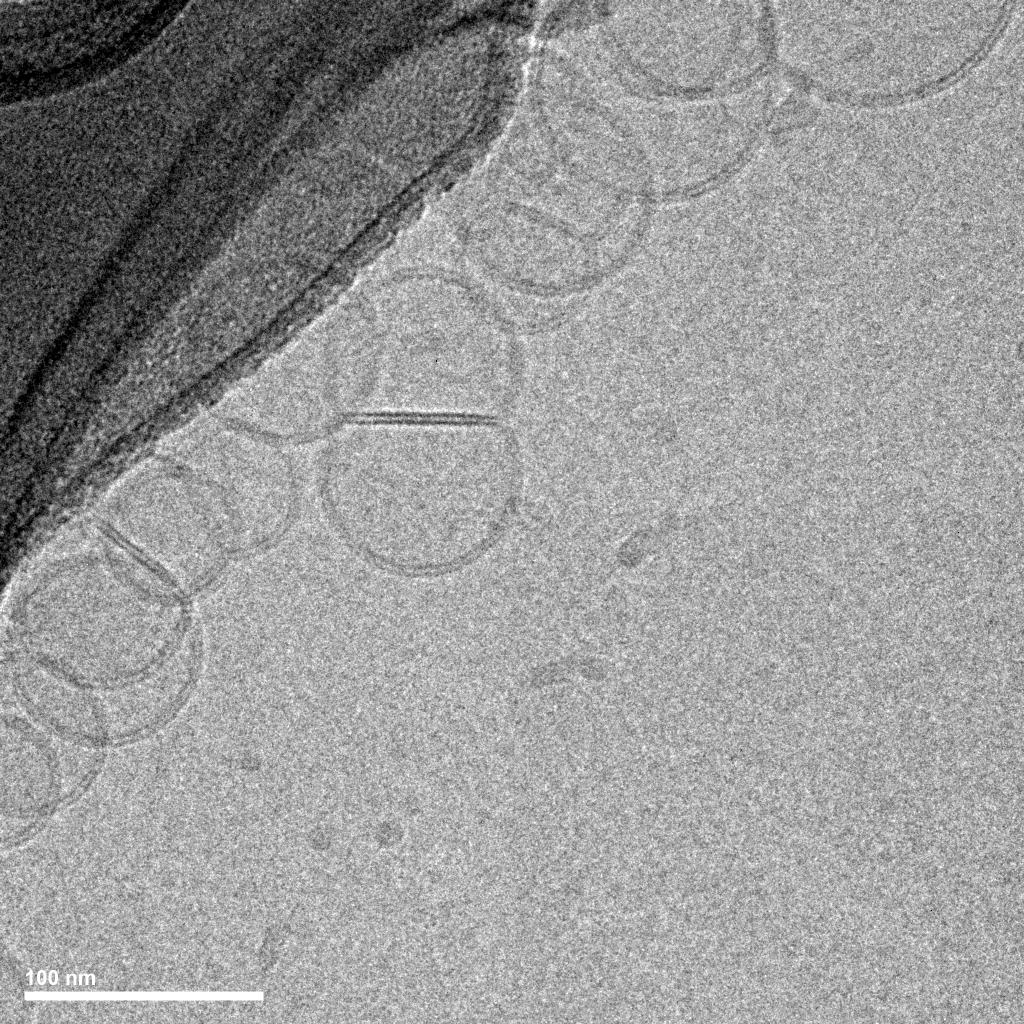

Supplement: Figure 2—source data 1. — This zip archive contains all cryo-EM images used for the quantitative analyses shown in Fig. 2. The folder named “No_Ca++” contains the images before Ca++ addition (individual files are named P3_1_**. tif or jpg), and folder named “With_Ca++” contains the images ∼35s after Ca++ addition (individual files are named P3_3_**.tif or jpg). Images were collected in low dose conditions at 200 kV acceleration voltage on a CM200 FEG electron microscope (FEI) with a 2k × 2k Gatan UltraScan 1000 camera, at 50,000× magnification and 1.5 mm underfocus. The full resolution data were exported as 16 bit “tif” files (2048 × 2048 pixels, scale 0.2 nm/pixel at specimen (the corresponding files have the extension “tif”). Note that these files cannot not be viewed with a standard picture viewer, but must be viewed with a program, such as “ImageJ”. To facilitate easier viewing, the original images were converted to smaller (1024×1024, 0.4 nm/pixel), contrast adjusted jpeg images (8 bits) for easy and immediate visualization with commonly used picture viewers (the corresponding files have the extension “jpg”). DOI: http://dx.doi.org/10.7554/eLife.00109.005 [file elife00109s001.zip › elife00109s001/With_Ca++/P3_3_20.jpg]

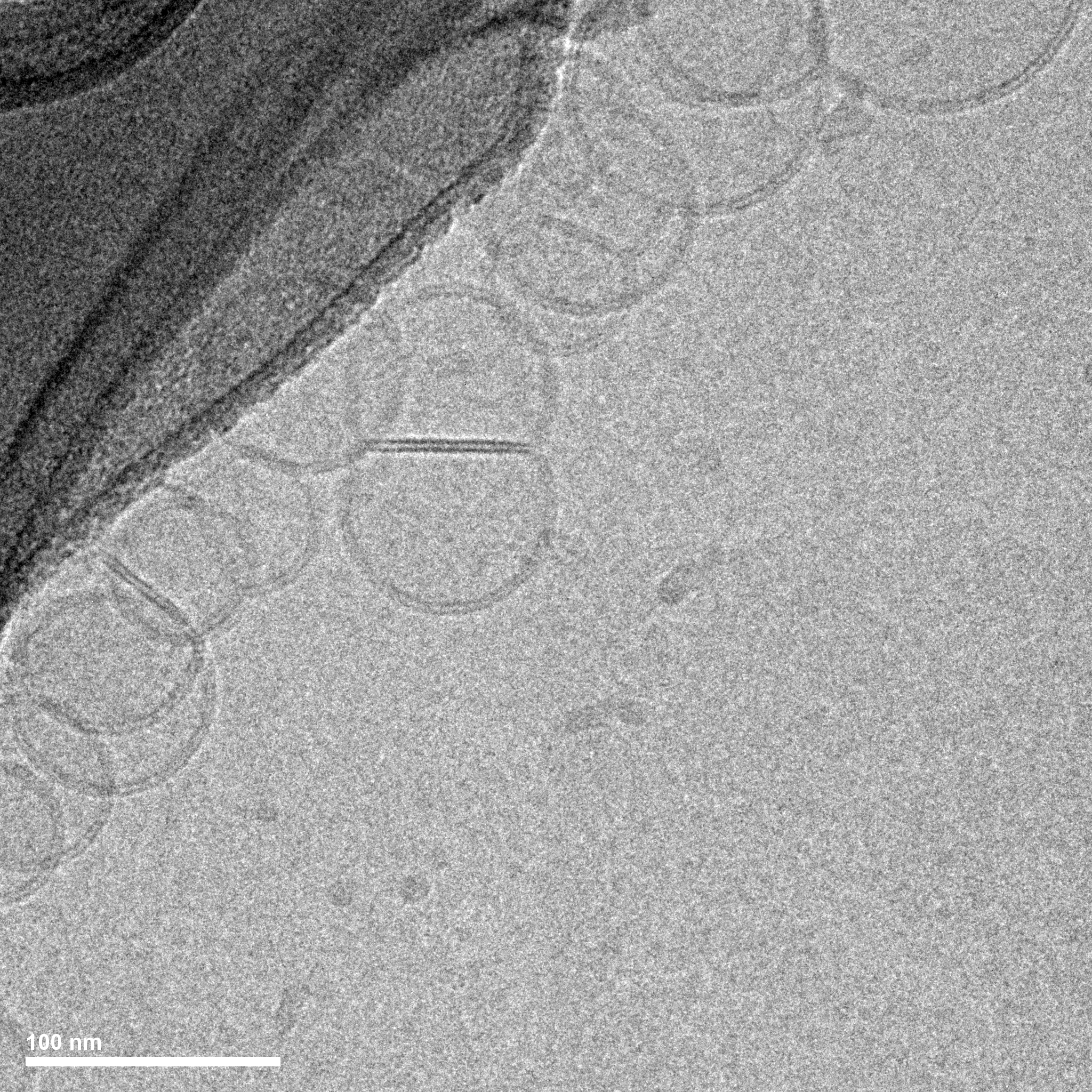

Supplement: Figure 2—source data 1. — This zip archive contains all cryo-EM images used for the quantitative analyses shown in Fig. 2. The folder named “No_Ca++” contains the images before Ca++ addition (individual files are named P3_1_**. tif or jpg), and folder named “With_Ca++” contains the images ∼35s after Ca++ addition (individual files are named P3_3_**.tif or jpg). Images were collected in low dose conditions at 200 kV acceleration voltage on a CM200 FEG electron microscope (FEI) with a 2k × 2k Gatan UltraScan 1000 camera, at 50,000× magnification and 1.5 mm underfocus. The full resolution data were exported as 16 bit “tif” files (2048 × 2048 pixels, scale 0.2 nm/pixel at specimen (the corresponding files have the extension “tif”). Note that these files cannot not be viewed with a standard picture viewer, but must be viewed with a program, such as “ImageJ”. To facilitate easier viewing, the original images were converted to smaller (1024×1024, 0.4 nm/pixel), contrast adjusted jpeg images (8 bits) for easy and immediate visualization with commonly used picture viewers (the corresponding files have the extension “jpg”). DOI: http://dx.doi.org/10.7554/eLife.00109.005 [file elife00109s001.zip › elife00109s001/With_Ca++/P3_3_20.tif]

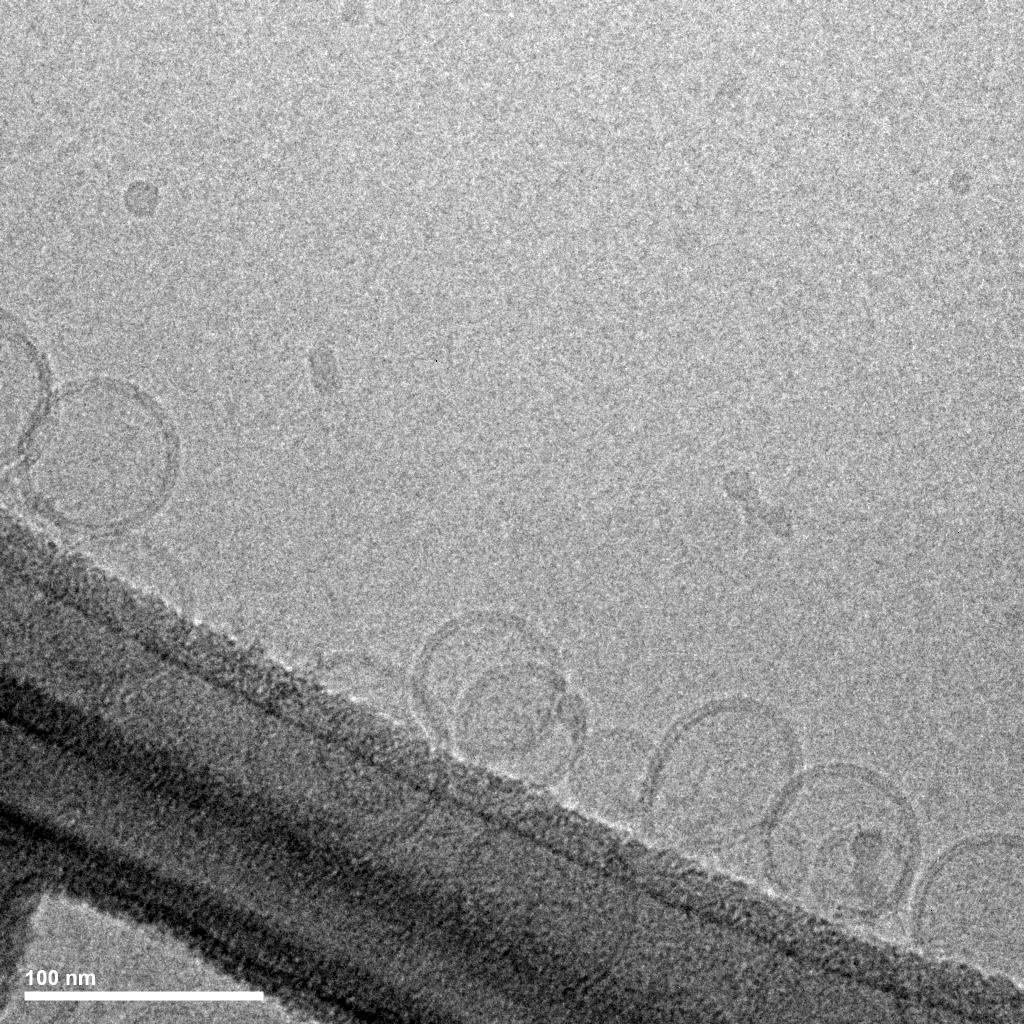

Supplement: Figure 2—source data 1. — This zip archive contains all cryo-EM images used for the quantitative analyses shown in Fig. 2. The folder named “No_Ca++” contains the images before Ca++ addition (individual files are named P3_1_**. tif or jpg), and folder named “With_Ca++” contains the images ∼35s after Ca++ addition (individual files are named P3_3_**.tif or jpg). Images were collected in low dose conditions at 200 kV acceleration voltage on a CM200 FEG electron microscope (FEI) with a 2k × 2k Gatan UltraScan 1000 camera, at 50,000× magnification and 1.5 mm underfocus. The full resolution data were exported as 16 bit “tif” files (2048 × 2048 pixels, scale 0.2 nm/pixel at specimen (the corresponding files have the extension “tif”). Note that these files cannot not be viewed with a standard picture viewer, but must be viewed with a program, such as “ImageJ”. To facilitate easier viewing, the original images were converted to smaller (1024×1024, 0.4 nm/pixel), contrast adjusted jpeg images (8 bits) for easy and immediate visualization with commonly used picture viewers (the corresponding files have the extension “jpg”). DOI: http://dx.doi.org/10.7554/eLife.00109.005 [file elife00109s001.zip › elife00109s001/With_Ca++/P3_3_21.jpg]

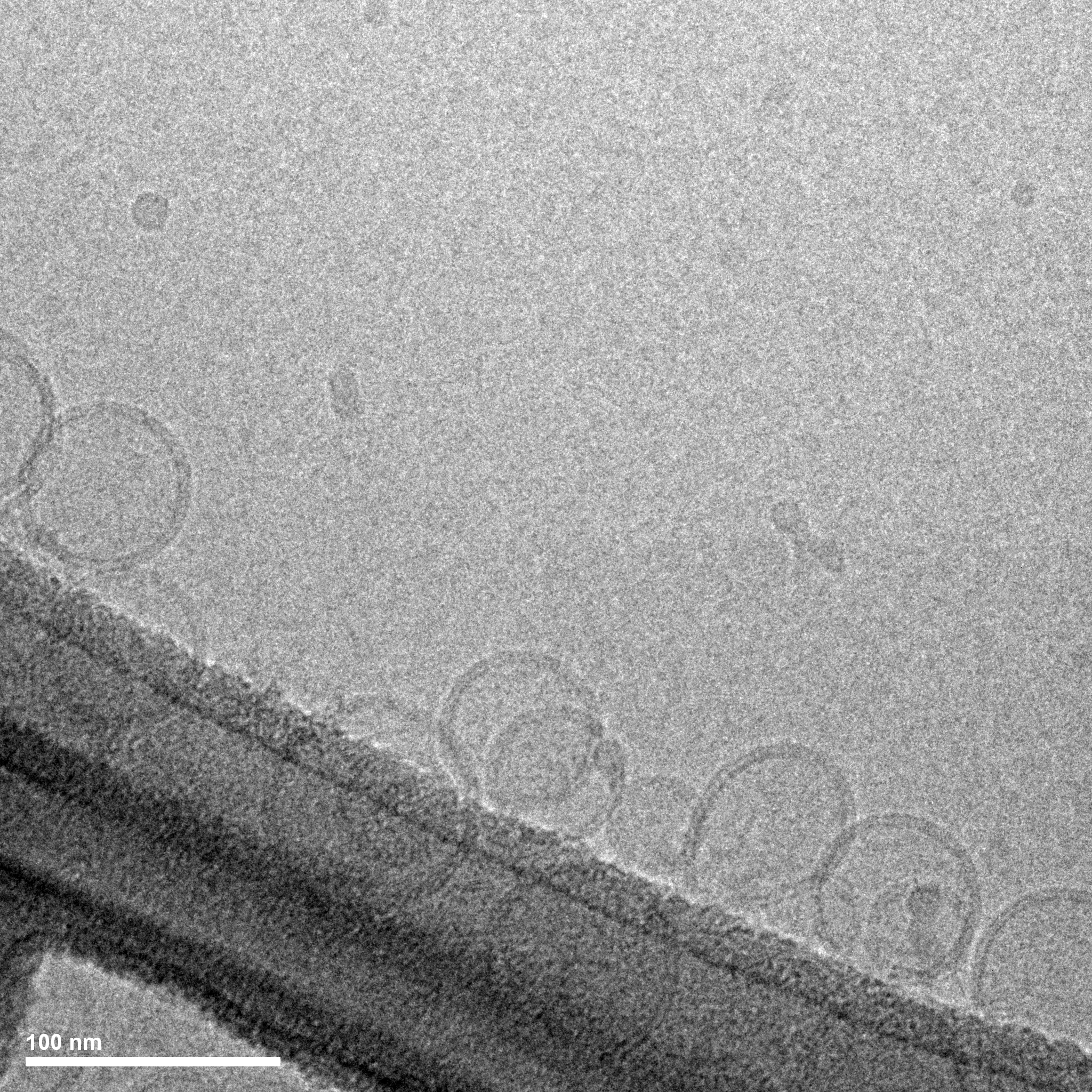

Supplement: Figure 2—source data 1. — This zip archive contains all cryo-EM images used for the quantitative analyses shown in Fig. 2. The folder named “No_Ca++” contains the images before Ca++ addition (individual files are named P3_1_**. tif or jpg), and folder named “With_Ca++” contains the images ∼35s after Ca++ addition (individual files are named P3_3_**.tif or jpg). Images were collected in low dose conditions at 200 kV acceleration voltage on a CM200 FEG electron microscope (FEI) with a 2k × 2k Gatan UltraScan 1000 camera, at 50,000× magnification and 1.5 mm underfocus. The full resolution data were exported as 16 bit “tif” files (2048 × 2048 pixels, scale 0.2 nm/pixel at specimen (the corresponding files have the extension “tif”). Note that these files cannot not be viewed with a standard picture viewer, but must be viewed with a program, such as “ImageJ”. To facilitate easier viewing, the original images were converted to smaller (1024×1024, 0.4 nm/pixel), contrast adjusted jpeg images (8 bits) for easy and immediate visualization with commonly used picture viewers (the corresponding files have the extension “jpg”). DOI: http://dx.doi.org/10.7554/eLife.00109.005 [file elife00109s001.zip › elife00109s001/With_Ca++/P3_3_21.tif]

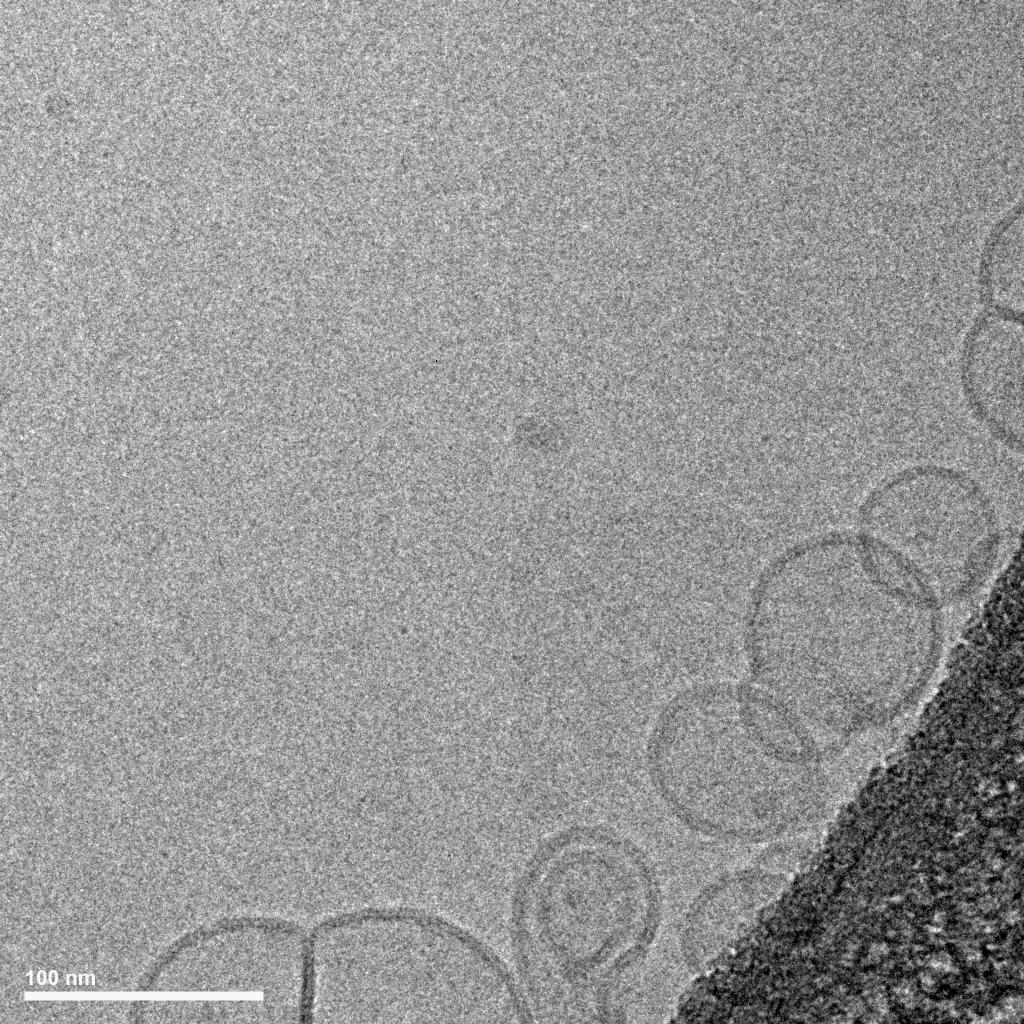

Supplement: Figure 2—source data 1. — This zip archive contains all cryo-EM images used for the quantitative analyses shown in Fig. 2. The folder named “No_Ca++” contains the images before Ca++ addition (individual files are named P3_1_**. tif or jpg), and folder named “With_Ca++” contains the images ∼35s after Ca++ addition (individual files are named P3_3_**.tif or jpg). Images were collected in low dose conditions at 200 kV acceleration voltage on a CM200 FEG electron microscope (FEI) with a 2k × 2k Gatan UltraScan 1000 camera, at 50,000× magnification and 1.5 mm underfocus. The full resolution data were exported as 16 bit “tif” files (2048 × 2048 pixels, scale 0.2 nm/pixel at specimen (the corresponding files have the extension “tif”). Note that these files cannot not be viewed with a standard picture viewer, but must be viewed with a program, such as “ImageJ”. To facilitate easier viewing, the original images were converted to smaller (1024×1024, 0.4 nm/pixel), contrast adjusted jpeg images (8 bits) for easy and immediate visualization with commonly used picture viewers (the corresponding files have the extension “jpg”). DOI: http://dx.doi.org/10.7554/eLife.00109.005 [file elife00109s001.zip › elife00109s001/With_Ca++/P3_3_23.jpg]

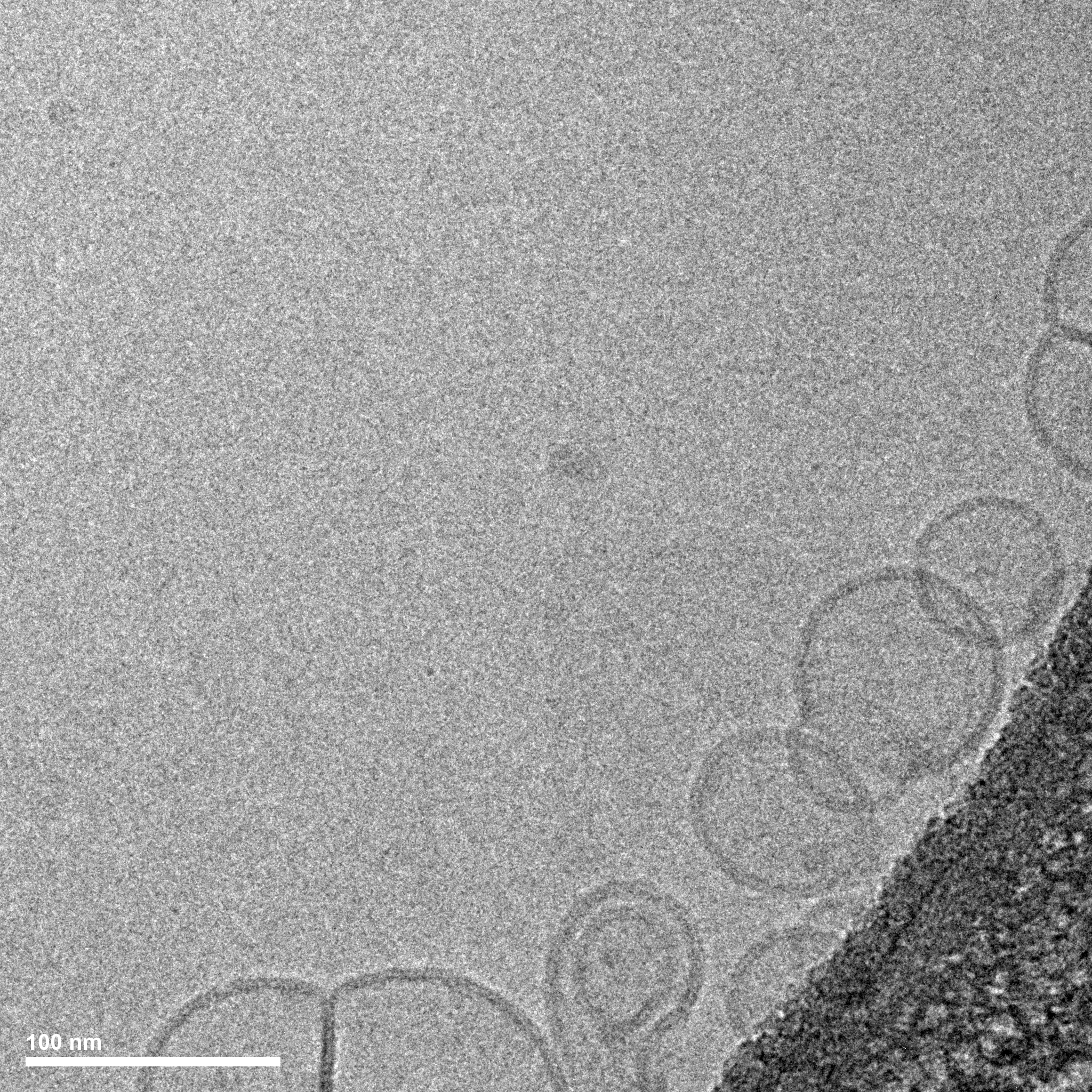

Supplement: Figure 2—source data 1. — This zip archive contains all cryo-EM images used for the quantitative analyses shown in Fig. 2. The folder named “No_Ca++” contains the images before Ca++ addition (individual files are named P3_1_**. tif or jpg), and folder named “With_Ca++” contains the images ∼35s after Ca++ addition (individual files are named P3_3_**.tif or jpg). Images were collected in low dose conditions at 200 kV acceleration voltage on a CM200 FEG electron microscope (FEI) with a 2k × 2k Gatan UltraScan 1000 camera, at 50,000× magnification and 1.5 mm underfocus. The full resolution data were exported as 16 bit “tif” files (2048 × 2048 pixels, scale 0.2 nm/pixel at specimen (the corresponding files have the extension “tif”). Note that these files cannot not be viewed with a standard picture viewer, but must be viewed with a program, such as “ImageJ”. To facilitate easier viewing, the original images were converted to smaller (1024×1024, 0.4 nm/pixel), contrast adjusted jpeg images (8 bits) for easy and immediate visualization with commonly used picture viewers (the corresponding files have the extension “jpg”). DOI: http://dx.doi.org/10.7554/eLife.00109.005 [file elife00109s001.zip › elife00109s001/With_Ca++/P3_3_23.tif]

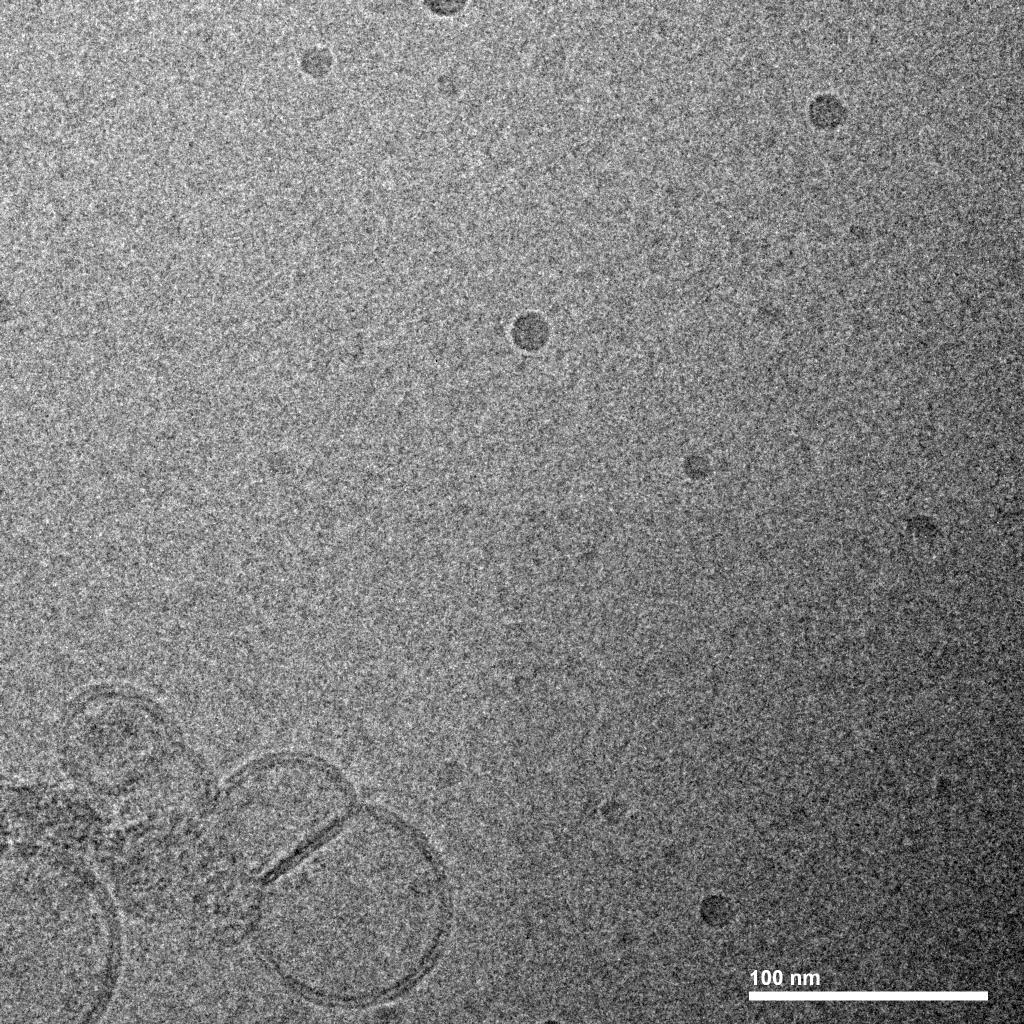

Supplement: Figure 2—source data 1. — This zip archive contains all cryo-EM images used for the quantitative analyses shown in Fig. 2. The folder named “No_Ca++” contains the images before Ca++ addition (individual files are named P3_1_**. tif or jpg), and folder named “With_Ca++” contains the images ∼35s after Ca++ addition (individual files are named P3_3_**.tif or jpg). Images were collected in low dose conditions at 200 kV acceleration voltage on a CM200 FEG electron microscope (FEI) with a 2k × 2k Gatan UltraScan 1000 camera, at 50,000× magnification and 1.5 mm underfocus. The full resolution data were exported as 16 bit “tif” files (2048 × 2048 pixels, scale 0.2 nm/pixel at specimen (the corresponding files have the extension “tif”). Note that these files cannot not be viewed with a standard picture viewer, but must be viewed with a program, such as “ImageJ”. To facilitate easier viewing, the original images were converted to smaller (1024×1024, 0.4 nm/pixel), contrast adjusted jpeg images (8 bits) for easy and immediate visualization with commonly used picture viewers (the corresponding files have the extension “jpg”). DOI: http://dx.doi.org/10.7554/eLife.00109.005 [file elife00109s001.zip › elife00109s001/With_Ca++/P3_3_24.jpg]

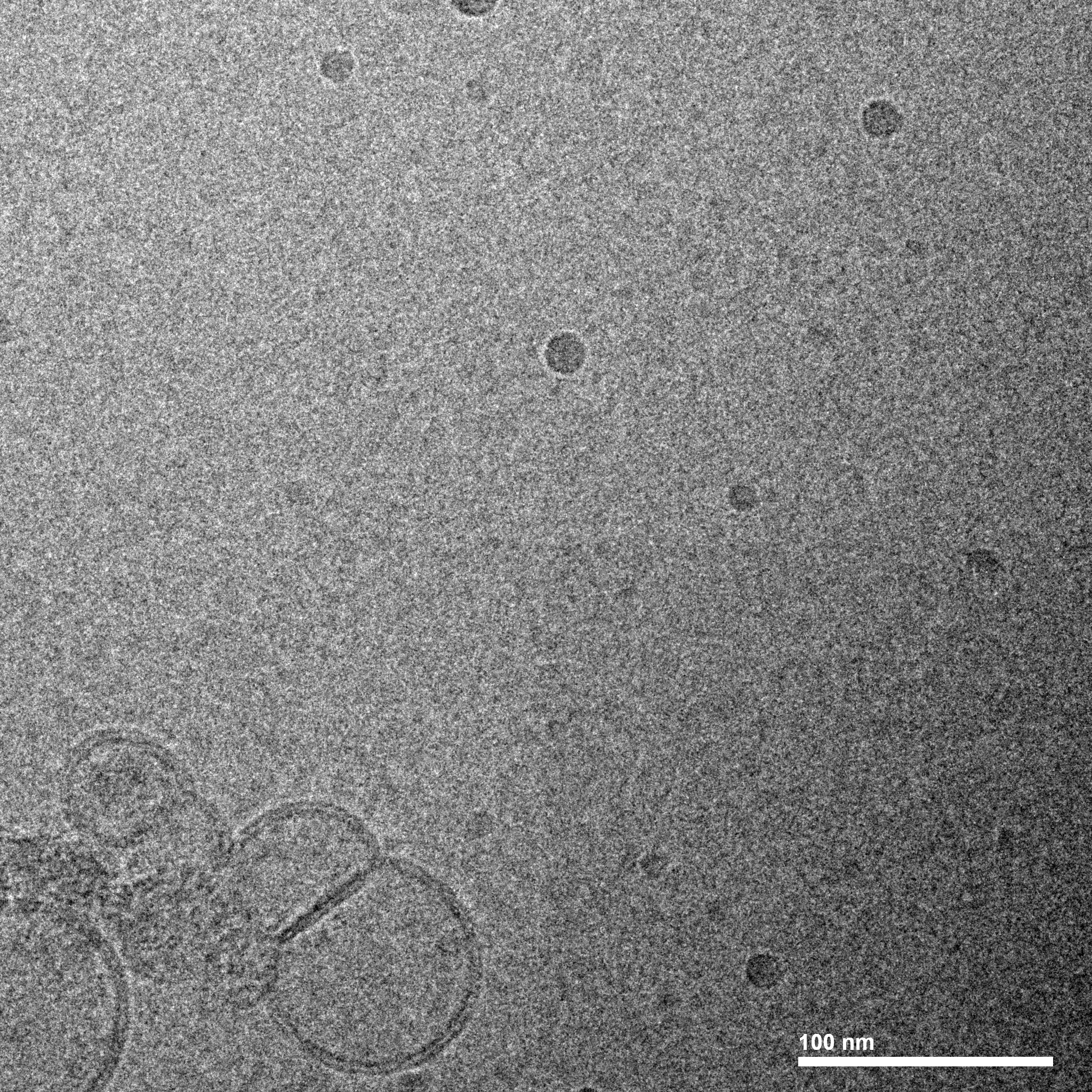

Supplement: Figure 2—source data 1. — This zip archive contains all cryo-EM images used for the quantitative analyses shown in Fig. 2. The folder named “No_Ca++” contains the images before Ca++ addition (individual files are named P3_1_**. tif or jpg), and folder named “With_Ca++” contains the images ∼35s after Ca++ addition (individual files are named P3_3_**.tif or jpg). Images were collected in low dose conditions at 200 kV acceleration voltage on a CM200 FEG electron microscope (FEI) with a 2k × 2k Gatan UltraScan 1000 camera, at 50,000× magnification and 1.5 mm underfocus. The full resolution data were exported as 16 bit “tif” files (2048 × 2048 pixels, scale 0.2 nm/pixel at specimen (the corresponding files have the extension “tif”). Note that these files cannot not be viewed with a standard picture viewer, but must be viewed with a program, such as “ImageJ”. To facilitate easier viewing, the original images were converted to smaller (1024×1024, 0.4 nm/pixel), contrast adjusted jpeg images (8 bits) for easy and immediate visualization with commonly used picture viewers (the corresponding files have the extension “jpg”). DOI: http://dx.doi.org/10.7554/eLife.00109.005 [file elife00109s001.zip › elife00109s001/With_Ca++/P3_3_24.tif]

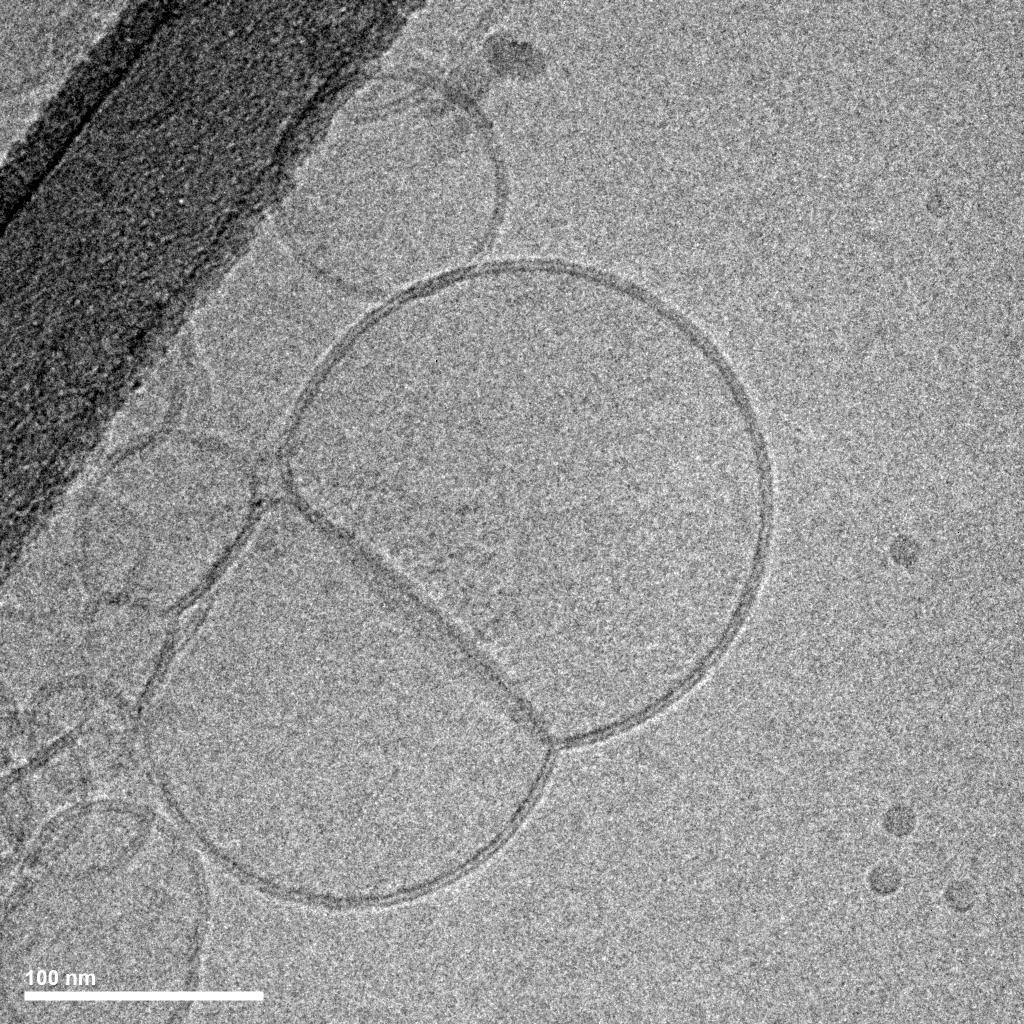

Supplement: Figure 2—source data 1. — This zip archive contains all cryo-EM images used for the quantitative analyses shown in Fig. 2. The folder named “No_Ca++” contains the images before Ca++ addition (individual files are named P3_1_**. tif or jpg), and folder named “With_Ca++” contains the images ∼35s after Ca++ addition (individual files are named P3_3_**.tif or jpg). Images were collected in low dose conditions at 200 kV acceleration voltage on a CM200 FEG electron microscope (FEI) with a 2k × 2k Gatan UltraScan 1000 camera, at 50,000× magnification and 1.5 mm underfocus. The full resolution data were exported as 16 bit “tif” files (2048 × 2048 pixels, scale 0.2 nm/pixel at specimen (the corresponding files have the extension “tif”). Note that these files cannot not be viewed with a standard picture viewer, but must be viewed with a program, such as “ImageJ”. To facilitate easier viewing, the original images were converted to smaller (1024×1024, 0.4 nm/pixel), contrast adjusted jpeg images (8 bits) for easy and immediate visualization with commonly used picture viewers (the corresponding files have the extension “jpg”). DOI: http://dx.doi.org/10.7554/eLife.00109.005 [file elife00109s001.zip › elife00109s001/With_Ca++/P3_3_26.jpg]

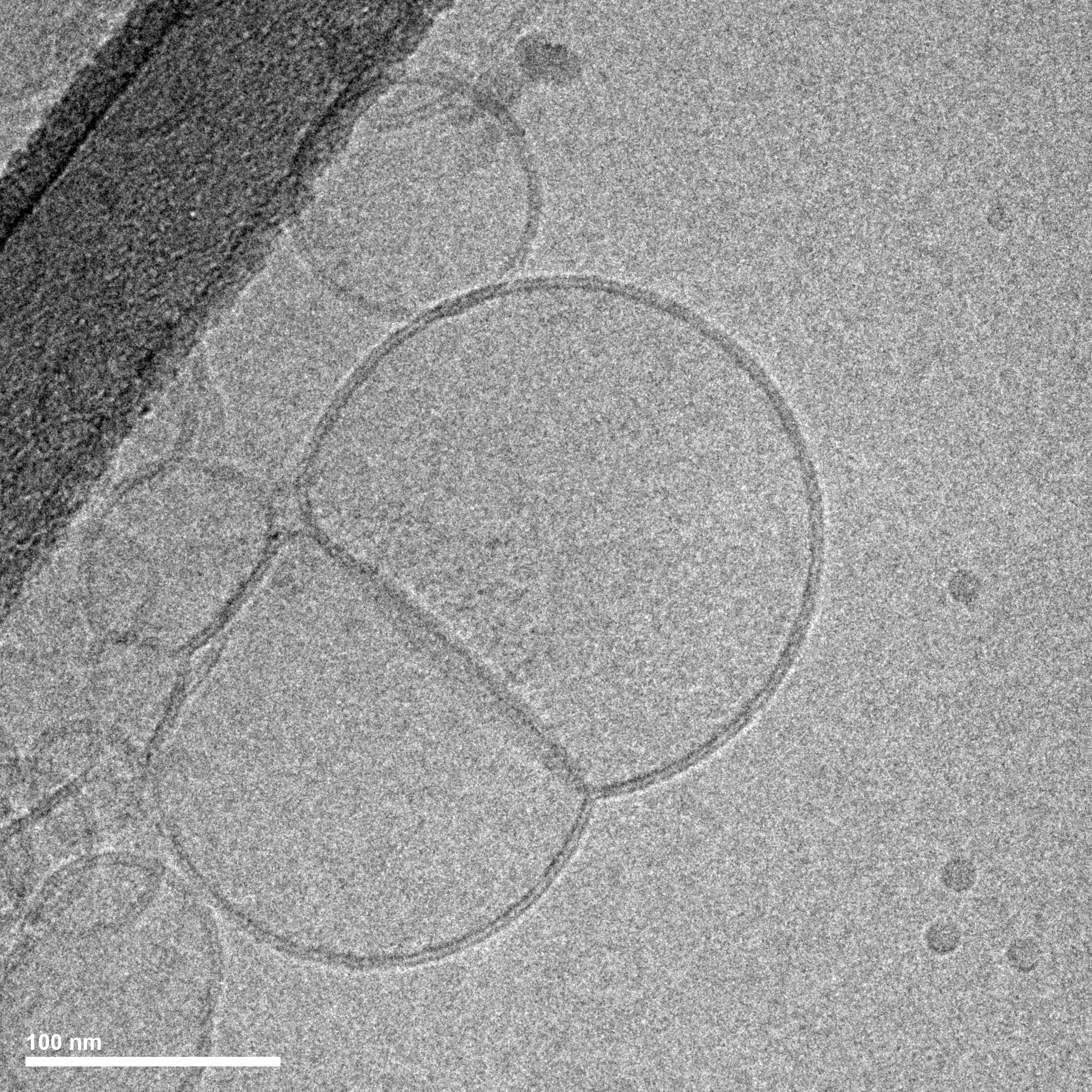

Supplement: Figure 2—source data 1. — This zip archive contains all cryo-EM images used for the quantitative analyses shown in Fig. 2. The folder named “No_Ca++” contains the images before Ca++ addition (individual files are named P3_1_**. tif or jpg), and folder named “With_Ca++” contains the images ∼35s after Ca++ addition (individual files are named P3_3_**.tif or jpg). Images were collected in low dose conditions at 200 kV acceleration voltage on a CM200 FEG electron microscope (FEI) with a 2k × 2k Gatan UltraScan 1000 camera, at 50,000× magnification and 1.5 mm underfocus. The full resolution data were exported as 16 bit “tif” files (2048 × 2048 pixels, scale 0.2 nm/pixel at specimen (the corresponding files have the extension “tif”). Note that these files cannot not be viewed with a standard picture viewer, but must be viewed with a program, such as “ImageJ”. To facilitate easier viewing, the original images were converted to smaller (1024×1024, 0.4 nm/pixel), contrast adjusted jpeg images (8 bits) for easy and immediate visualization with commonly used picture viewers (the corresponding files have the extension “jpg”). DOI: http://dx.doi.org/10.7554/eLife.00109.005 [file elife00109s001.zip › elife00109s001/With_Ca++/P3_3_26.tif]

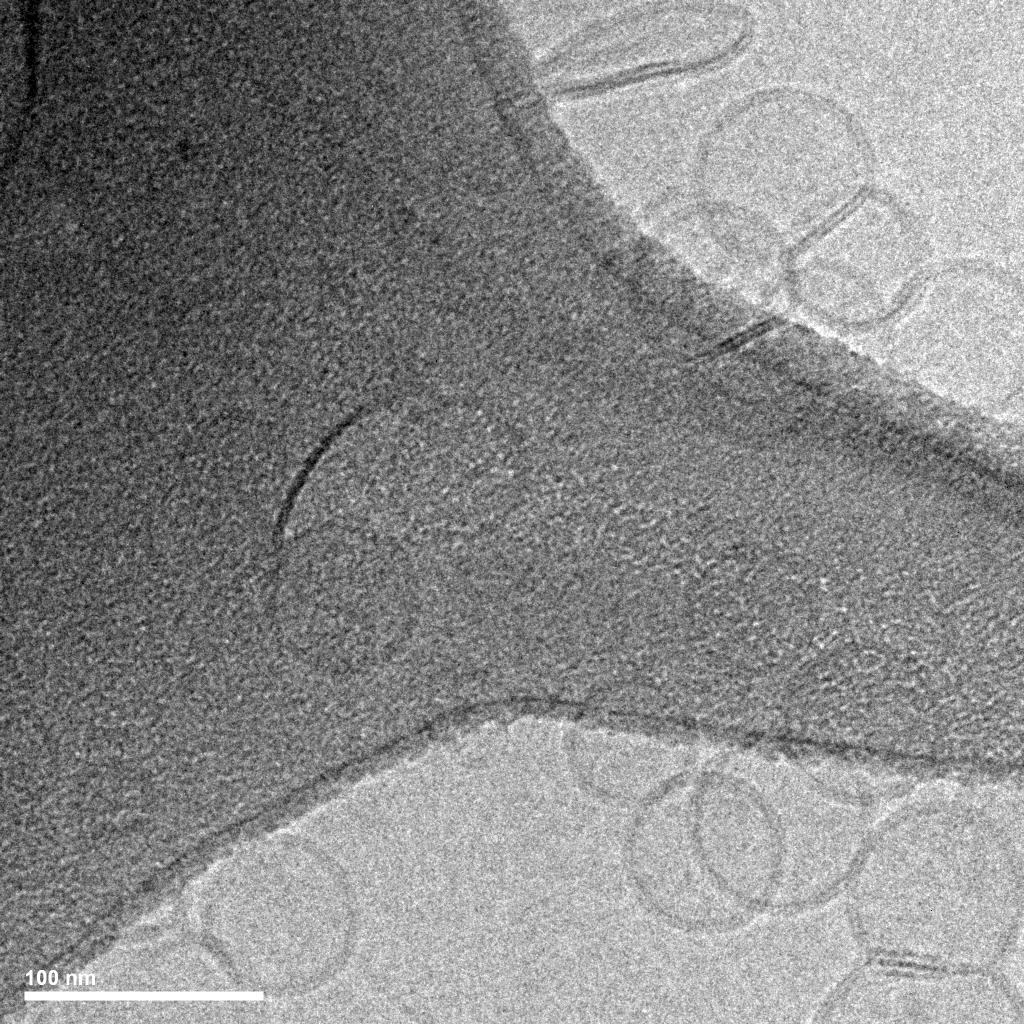

Supplement: Figure 2—source data 1. — This zip archive contains all cryo-EM images used for the quantitative analyses shown in Fig. 2. The folder named “No_Ca++” contains the images before Ca++ addition (individual files are named P3_1_**. tif or jpg), and folder named “With_Ca++” contains the images ∼35s after Ca++ addition (individual files are named P3_3_**.tif or jpg). Images were collected in low dose conditions at 200 kV acceleration voltage on a CM200 FEG electron microscope (FEI) with a 2k × 2k Gatan UltraScan 1000 camera, at 50,000× magnification and 1.5 mm underfocus. The full resolution data were exported as 16 bit “tif” files (2048 × 2048 pixels, scale 0.2 nm/pixel at specimen (the corresponding files have the extension “tif”). Note that these files cannot not be viewed with a standard picture viewer, but must be viewed with a program, such as “ImageJ”. To facilitate easier viewing, the original images were converted to smaller (1024×1024, 0.4 nm/pixel), contrast adjusted jpeg images (8 bits) for easy and immediate visualization with commonly used picture viewers (the corresponding files have the extension “jpg”). DOI: http://dx.doi.org/10.7554/eLife.00109.005 [file elife00109s001.zip › elife00109s001/With_Ca++/P3_3_29.jpg]

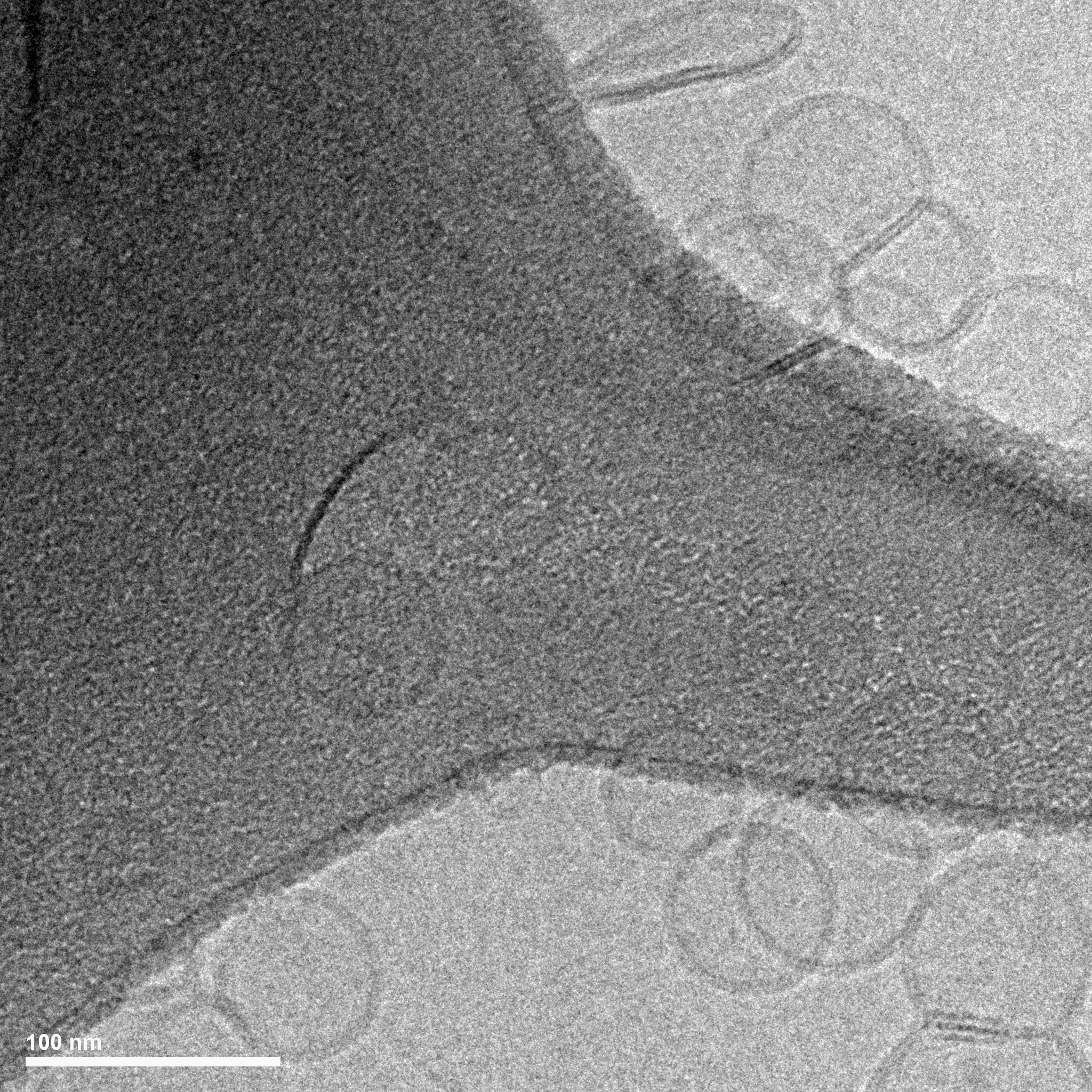

Supplement: Figure 2—source data 1. — This zip archive contains all cryo-EM images used for the quantitative analyses shown in Fig. 2. The folder named “No_Ca++” contains the images before Ca++ addition (individual files are named P3_1_**. tif or jpg), and folder named “With_Ca++” contains the images ∼35s after Ca++ addition (individual files are named P3_3_**.tif or jpg). Images were collected in low dose conditions at 200 kV acceleration voltage on a CM200 FEG electron microscope (FEI) with a 2k × 2k Gatan UltraScan 1000 camera, at 50,000× magnification and 1.5 mm underfocus. The full resolution data were exported as 16 bit “tif” files (2048 × 2048 pixels, scale 0.2 nm/pixel at specimen (the corresponding files have the extension “tif”). Note that these files cannot not be viewed with a standard picture viewer, but must be viewed with a program, such as “ImageJ”. To facilitate easier viewing, the original images were converted to smaller (1024×1024, 0.4 nm/pixel), contrast adjusted jpeg images (8 bits) for easy and immediate visualization with commonly used picture viewers (the corresponding files have the extension “jpg”). DOI: http://dx.doi.org/10.7554/eLife.00109.005 [file elife00109s001.zip › elife00109s001/With_Ca++/P3_3_29.tif]

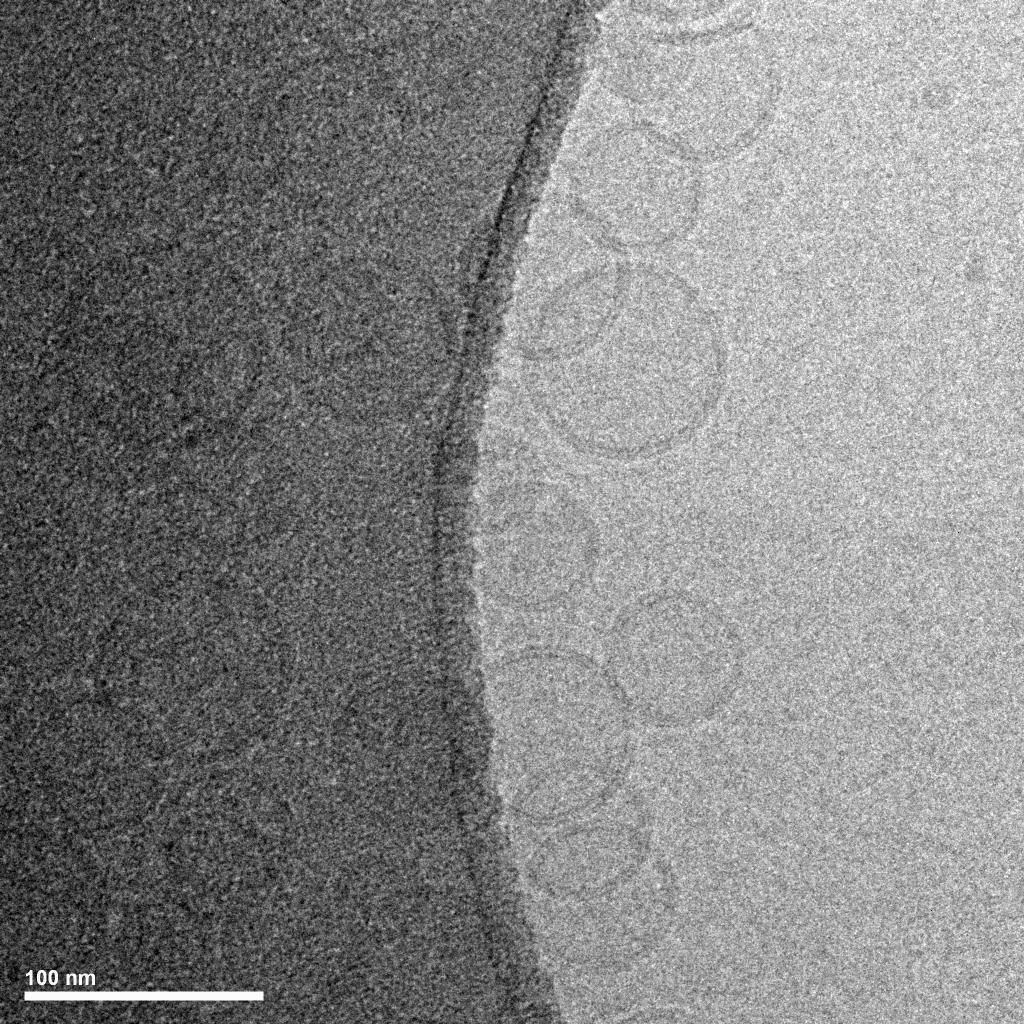

Supplement: Figure 2—source data 1. — This zip archive contains all cryo-EM images used for the quantitative analyses shown in Fig. 2. The folder named “No_Ca++” contains the images before Ca++ addition (individual files are named P3_1_**. tif or jpg), and folder named “With_Ca++” contains the images ∼35s after Ca++ addition (individual files are named P3_3_**.tif or jpg). Images were collected in low dose conditions at 200 kV acceleration voltage on a CM200 FEG electron microscope (FEI) with a 2k × 2k Gatan UltraScan 1000 camera, at 50,000× magnification and 1.5 mm underfocus. The full resolution data were exported as 16 bit “tif” files (2048 × 2048 pixels, scale 0.2 nm/pixel at specimen (the corresponding files have the extension “tif”). Note that these files cannot not be viewed with a standard picture viewer, but must be viewed with a program, such as “ImageJ”. To facilitate easier viewing, the original images were converted to smaller (1024×1024, 0.4 nm/pixel), contrast adjusted jpeg images (8 bits) for easy and immediate visualization with commonly used picture viewers (the corresponding files have the extension “jpg”). DOI: http://dx.doi.org/10.7554/eLife.00109.005 [file elife00109s001.zip › elife00109s001/With_Ca++/P3_3_31.jpg]

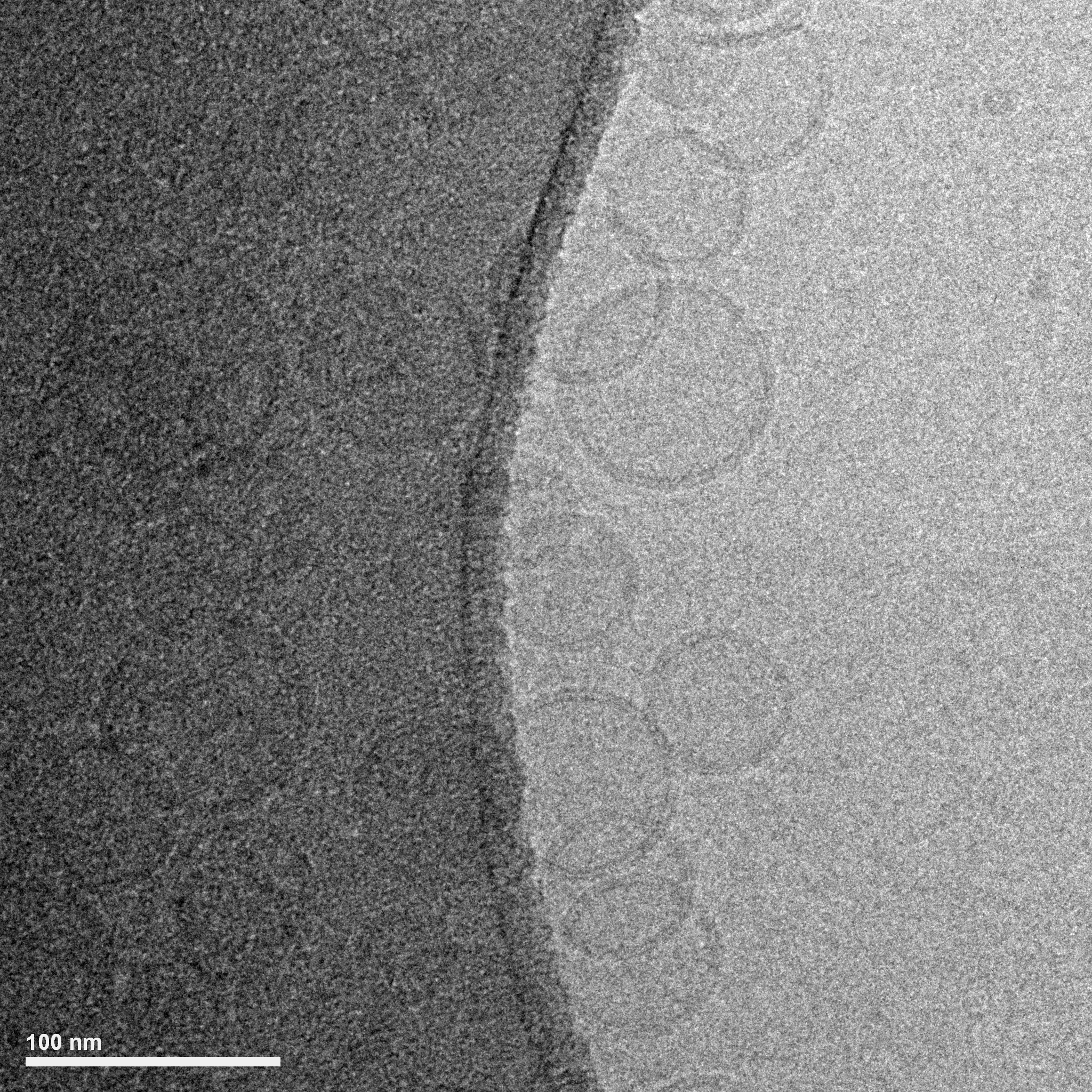

Supplement: Figure 2—source data 1. — This zip archive contains all cryo-EM images used for the quantitative analyses shown in Fig. 2. The folder named “No_Ca++” contains the images before Ca++ addition (individual files are named P3_1_**. tif or jpg), and folder named “With_Ca++” contains the images ∼35s after Ca++ addition (individual files are named P3_3_**.tif or jpg). Images were collected in low dose conditions at 200 kV acceleration voltage on a CM200 FEG electron microscope (FEI) with a 2k × 2k Gatan UltraScan 1000 camera, at 50,000× magnification and 1.5 mm underfocus. The full resolution data were exported as 16 bit “tif” files (2048 × 2048 pixels, scale 0.2 nm/pixel at specimen (the corresponding files have the extension “tif”). Note that these files cannot not be viewed with a standard picture viewer, but must be viewed with a program, such as “ImageJ”. To facilitate easier viewing, the original images were converted to smaller (1024×1024, 0.4 nm/pixel), contrast adjusted jpeg images (8 bits) for easy and immediate visualization with commonly used picture viewers (the corresponding files have the extension “jpg”). DOI: http://dx.doi.org/10.7554/eLife.00109.005 [file elife00109s001.zip › elife00109s001/With_Ca++/P3_3_31.tif]

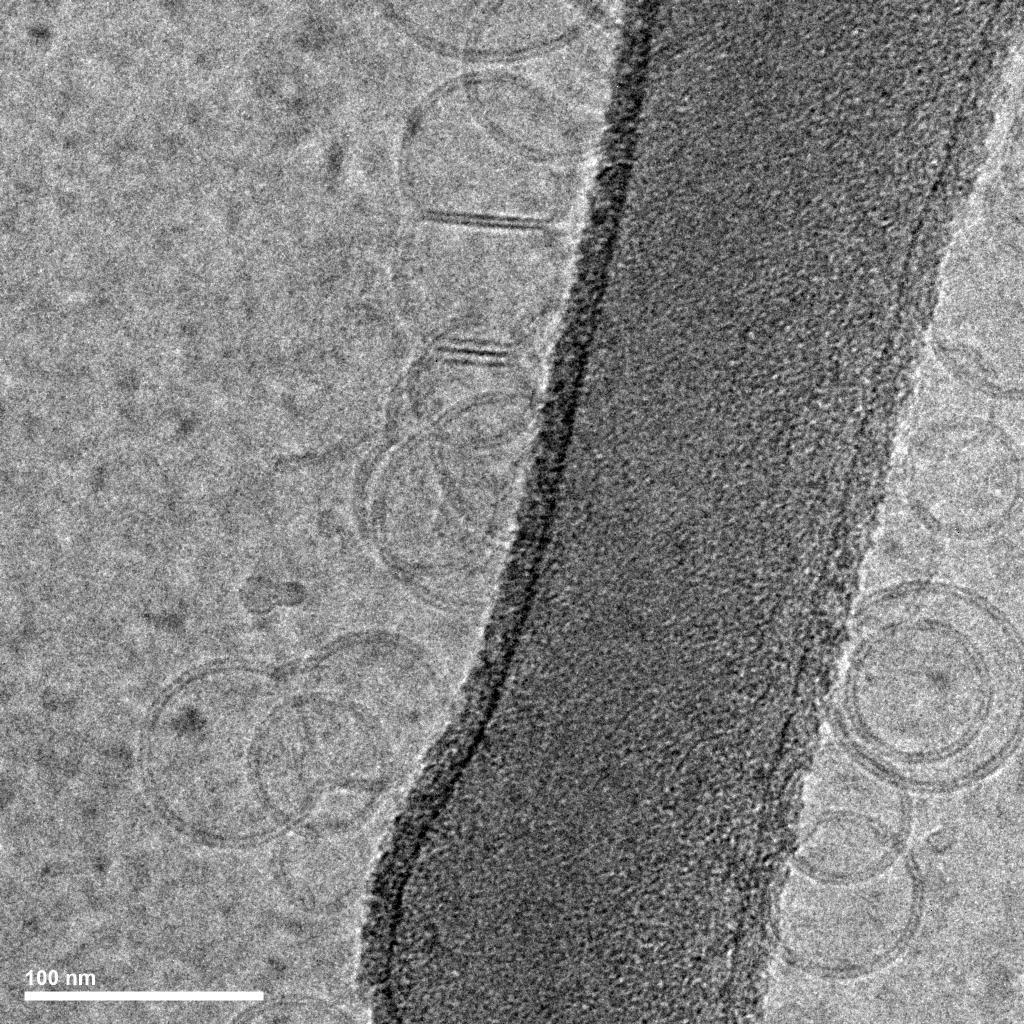

Supplement: Figure 2—source data 1. — This zip archive contains all cryo-EM images used for the quantitative analyses shown in Fig. 2. The folder named “No_Ca++” contains the images before Ca++ addition (individual files are named P3_1_**. tif or jpg), and folder named “With_Ca++” contains the images ∼35s after Ca++ addition (individual files are named P3_3_**.tif or jpg). Images were collected in low dose conditions at 200 kV acceleration voltage on a CM200 FEG electron microscope (FEI) with a 2k × 2k Gatan UltraScan 1000 camera, at 50,000× magnification and 1.5 mm underfocus. The full resolution data were exported as 16 bit “tif” files (2048 × 2048 pixels, scale 0.2 nm/pixel at specimen (the corresponding files have the extension “tif”). Note that these files cannot not be viewed with a standard picture viewer, but must be viewed with a program, such as “ImageJ”. To facilitate easier viewing, the original images were converted to smaller (1024×1024, 0.4 nm/pixel), contrast adjusted jpeg images (8 bits) for easy and immediate visualization with commonly used picture viewers (the corresponding files have the extension “jpg”). DOI: http://dx.doi.org/10.7554/eLife.00109.005 [file elife00109s001.zip › elife00109s001/With_Ca++/P3_3_32.jpg]

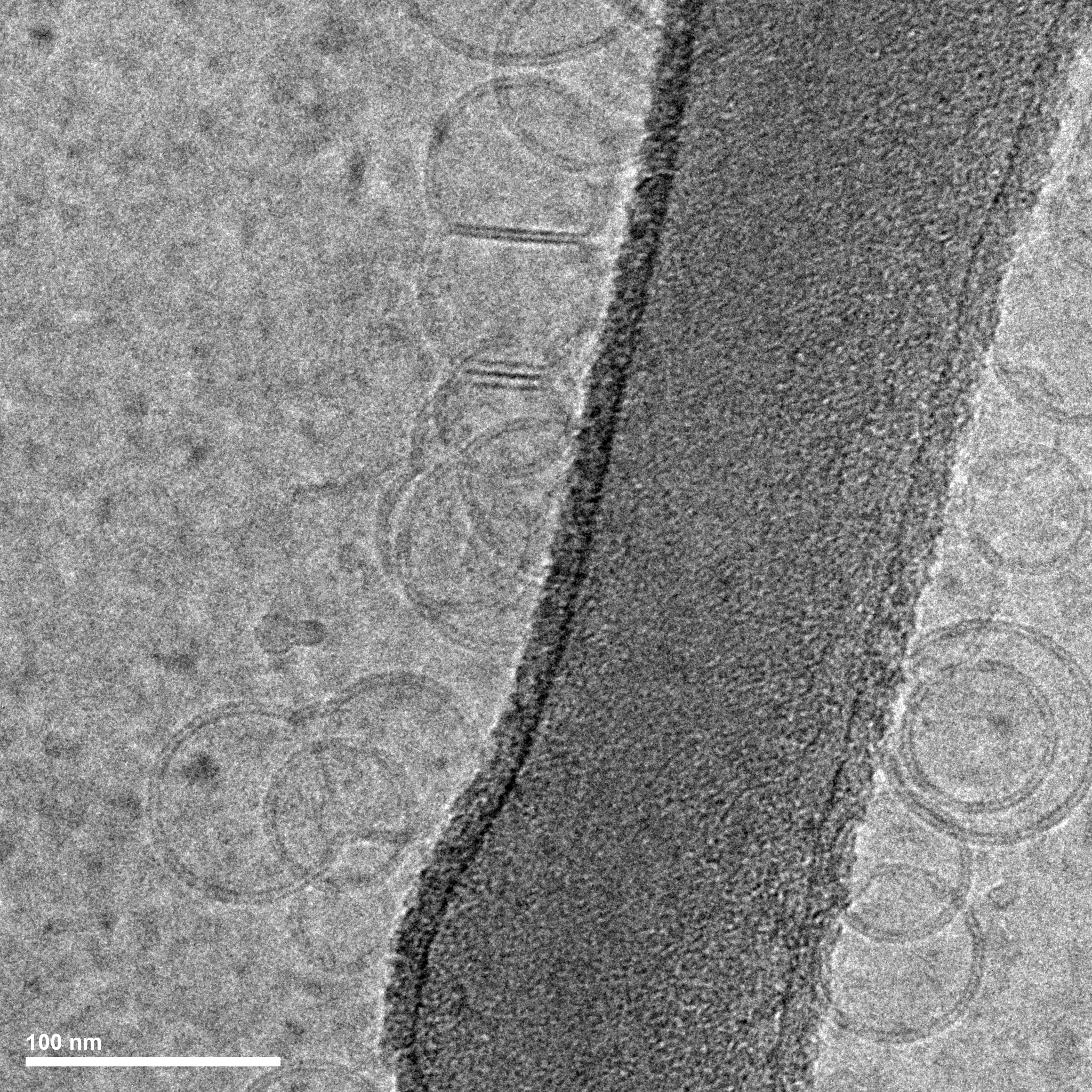

Supplement: Figure 2—source data 1. — This zip archive contains all cryo-EM images used for the quantitative analyses shown in Fig. 2. The folder named “No_Ca++” contains the images before Ca++ addition (individual files are named P3_1_**. tif or jpg), and folder named “With_Ca++” contains the images ∼35s after Ca++ addition (individual files are named P3_3_**.tif or jpg). Images were collected in low dose conditions at 200 kV acceleration voltage on a CM200 FEG electron microscope (FEI) with a 2k × 2k Gatan UltraScan 1000 camera, at 50,000× magnification and 1.5 mm underfocus. The full resolution data were exported as 16 bit “tif” files (2048 × 2048 pixels, scale 0.2 nm/pixel at specimen (the corresponding files have the extension “tif”). Note that these files cannot not be viewed with a standard picture viewer, but must be viewed with a program, such as “ImageJ”. To facilitate easier viewing, the original images were converted to smaller (1024×1024, 0.4 nm/pixel), contrast adjusted jpeg images (8 bits) for easy and immediate visualization with commonly used picture viewers (the corresponding files have the extension “jpg”). DOI: http://dx.doi.org/10.7554/eLife.00109.005 [file elife00109s001.zip › elife00109s001/With_Ca++/P3_3_32.tif]

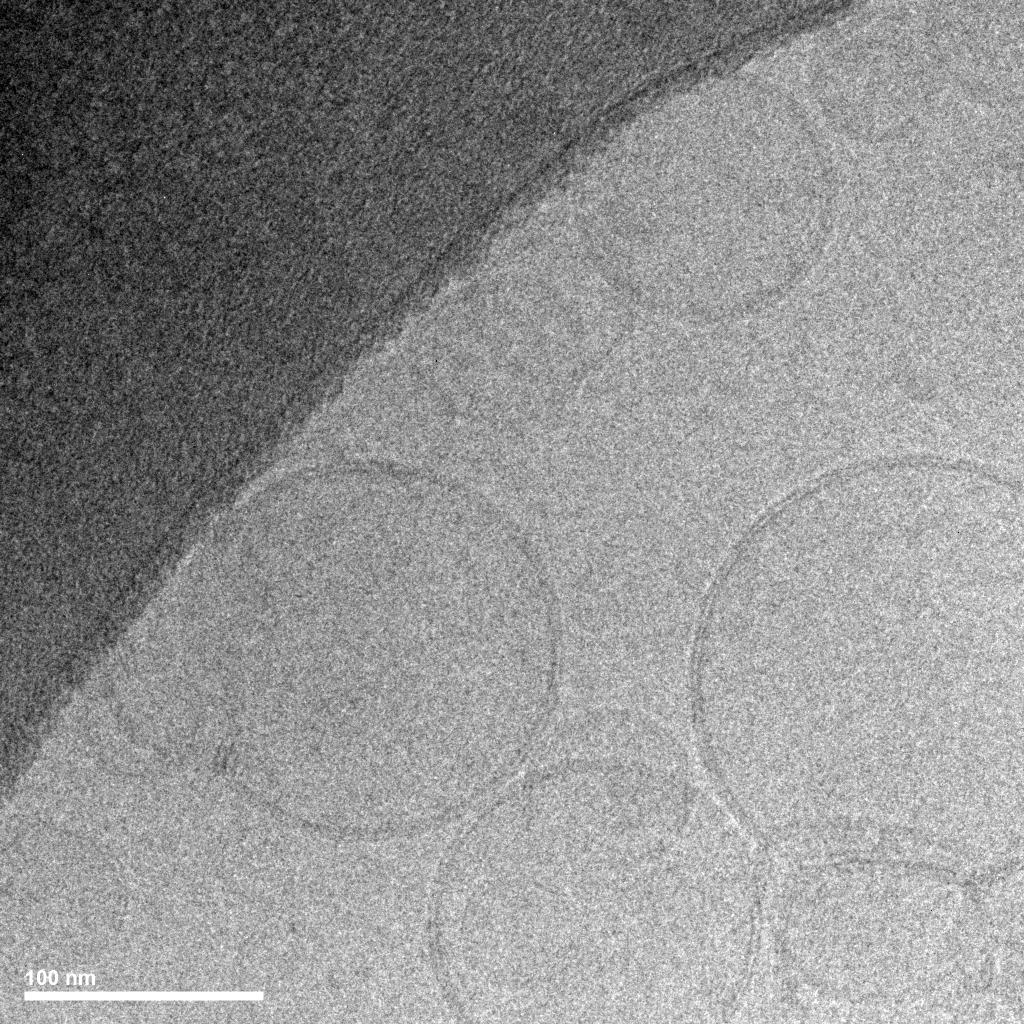

Supplement: Figure 2—source data 1. — This zip archive contains all cryo-EM images used for the quantitative analyses shown in Fig. 2. The folder named “No_Ca++” contains the images before Ca++ addition (individual files are named P3_1_**. tif or jpg), and folder named “With_Ca++” contains the images ∼35s after Ca++ addition (individual files are named P3_3_**.tif or jpg). Images were collected in low dose conditions at 200 kV acceleration voltage on a CM200 FEG electron microscope (FEI) with a 2k × 2k Gatan UltraScan 1000 camera, at 50,000× magnification and 1.5 mm underfocus. The full resolution data were exported as 16 bit “tif” files (2048 × 2048 pixels, scale 0.2 nm/pixel at specimen (the corresponding files have the extension “tif”). Note that these files cannot not be viewed with a standard picture viewer, but must be viewed with a program, such as “ImageJ”. To facilitate easier viewing, the original images were converted to smaller (1024×1024, 0.4 nm/pixel), contrast adjusted jpeg images (8 bits) for easy and immediate visualization with commonly used picture viewers (the corresponding files have the extension “jpg”). DOI: http://dx.doi.org/10.7554/eLife.00109.005 [file elife00109s001.zip › elife00109s001/With_Ca++/P3_3_33.jpg]

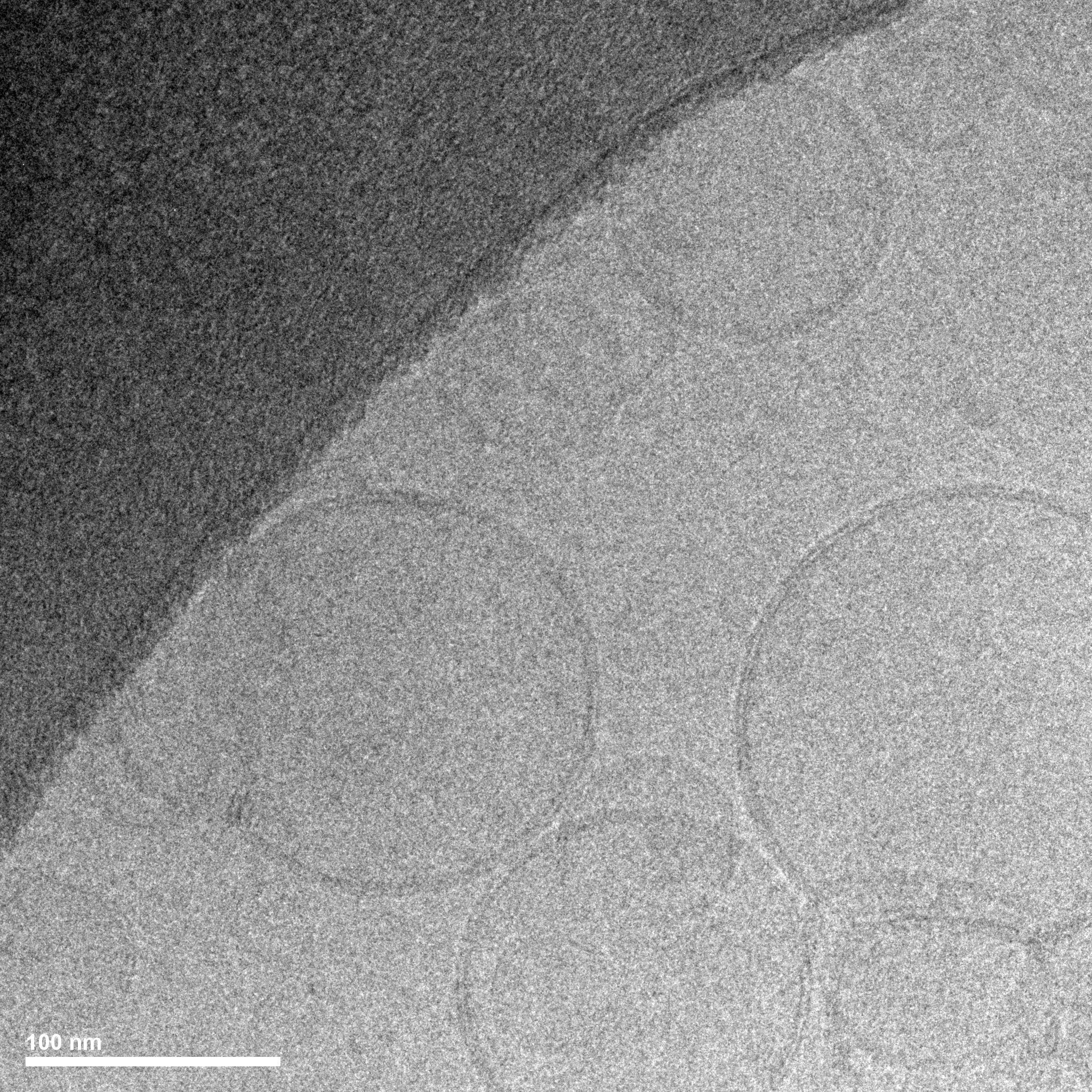

Supplement: Figure 2—source data 1. — This zip archive contains all cryo-EM images used for the quantitative analyses shown in Fig. 2. The folder named “No_Ca++” contains the images before Ca++ addition (individual files are named P3_1_**. tif or jpg), and folder named “With_Ca++” contains the images ∼35s after Ca++ addition (individual files are named P3_3_**.tif or jpg). Images were collected in low dose conditions at 200 kV acceleration voltage on a CM200 FEG electron microscope (FEI) with a 2k × 2k Gatan UltraScan 1000 camera, at 50,000× magnification and 1.5 mm underfocus. The full resolution data were exported as 16 bit “tif” files (2048 × 2048 pixels, scale 0.2 nm/pixel at specimen (the corresponding files have the extension “tif”). Note that these files cannot not be viewed with a standard picture viewer, but must be viewed with a program, such as “ImageJ”. To facilitate easier viewing, the original images were converted to smaller (1024×1024, 0.4 nm/pixel), contrast adjusted jpeg images (8 bits) for easy and immediate visualization with commonly used picture viewers (the corresponding files have the extension “jpg”). DOI: http://dx.doi.org/10.7554/eLife.00109.005 [file elife00109s001.zip › elife00109s001/With_Ca++/P3_3_33.tif]

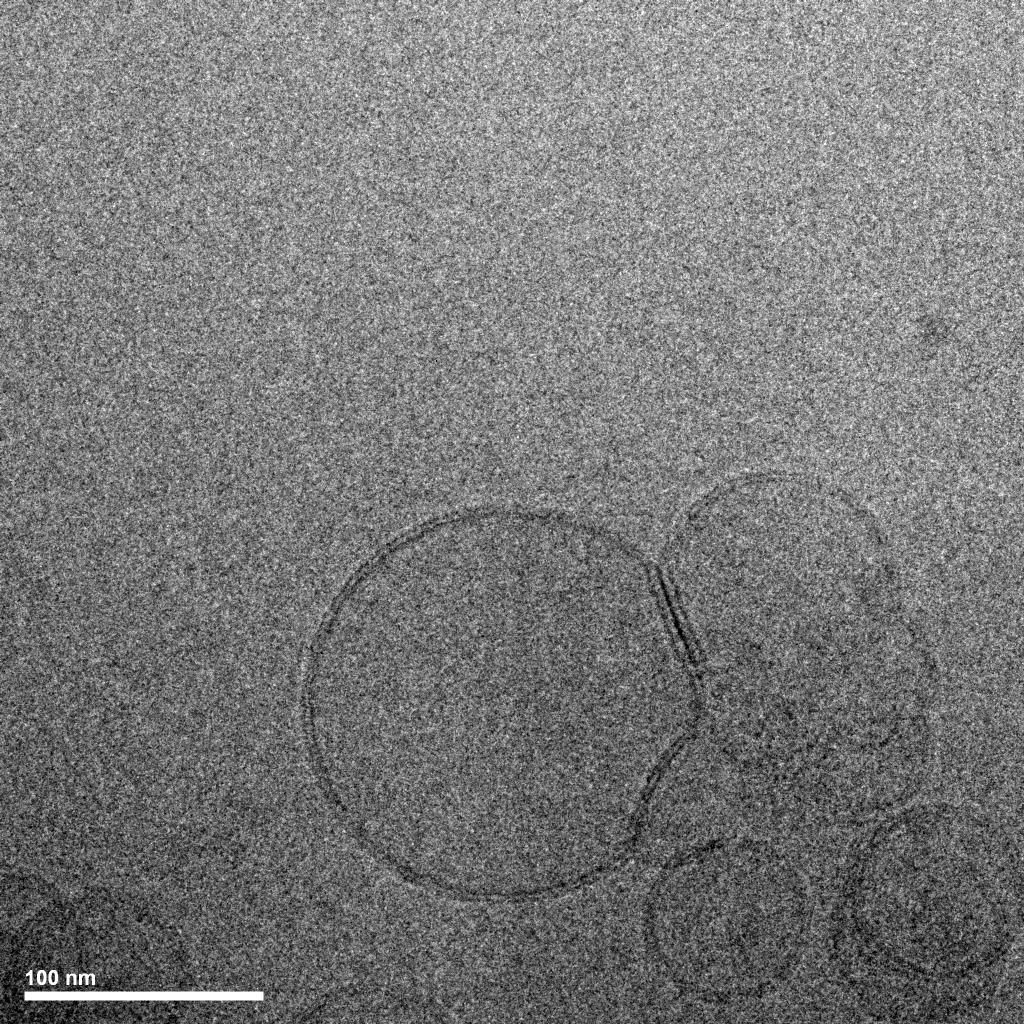

Supplement: Figure 2—source data 1. — This zip archive contains all cryo-EM images used for the quantitative analyses shown in Fig. 2. The folder named “No_Ca++” contains the images before Ca++ addition (individual files are named P3_1_**. tif or jpg), and folder named “With_Ca++” contains the images ∼35s after Ca++ addition (individual files are named P3_3_**.tif or jpg). Images were collected in low dose conditions at 200 kV acceleration voltage on a CM200 FEG electron microscope (FEI) with a 2k × 2k Gatan UltraScan 1000 camera, at 50,000× magnification and 1.5 mm underfocus. The full resolution data were exported as 16 bit “tif” files (2048 × 2048 pixels, scale 0.2 nm/pixel at specimen (the corresponding files have the extension “tif”). Note that these files cannot not be viewed with a standard picture viewer, but must be viewed with a program, such as “ImageJ”. To facilitate easier viewing, the original images were converted to smaller (1024×1024, 0.4 nm/pixel), contrast adjusted jpeg images (8 bits) for easy and immediate visualization with commonly used picture viewers (the corresponding files have the extension “jpg”). DOI: http://dx.doi.org/10.7554/eLife.00109.005 [file elife00109s001.zip › elife00109s001/With_Ca++/P3_3_35.jpg]

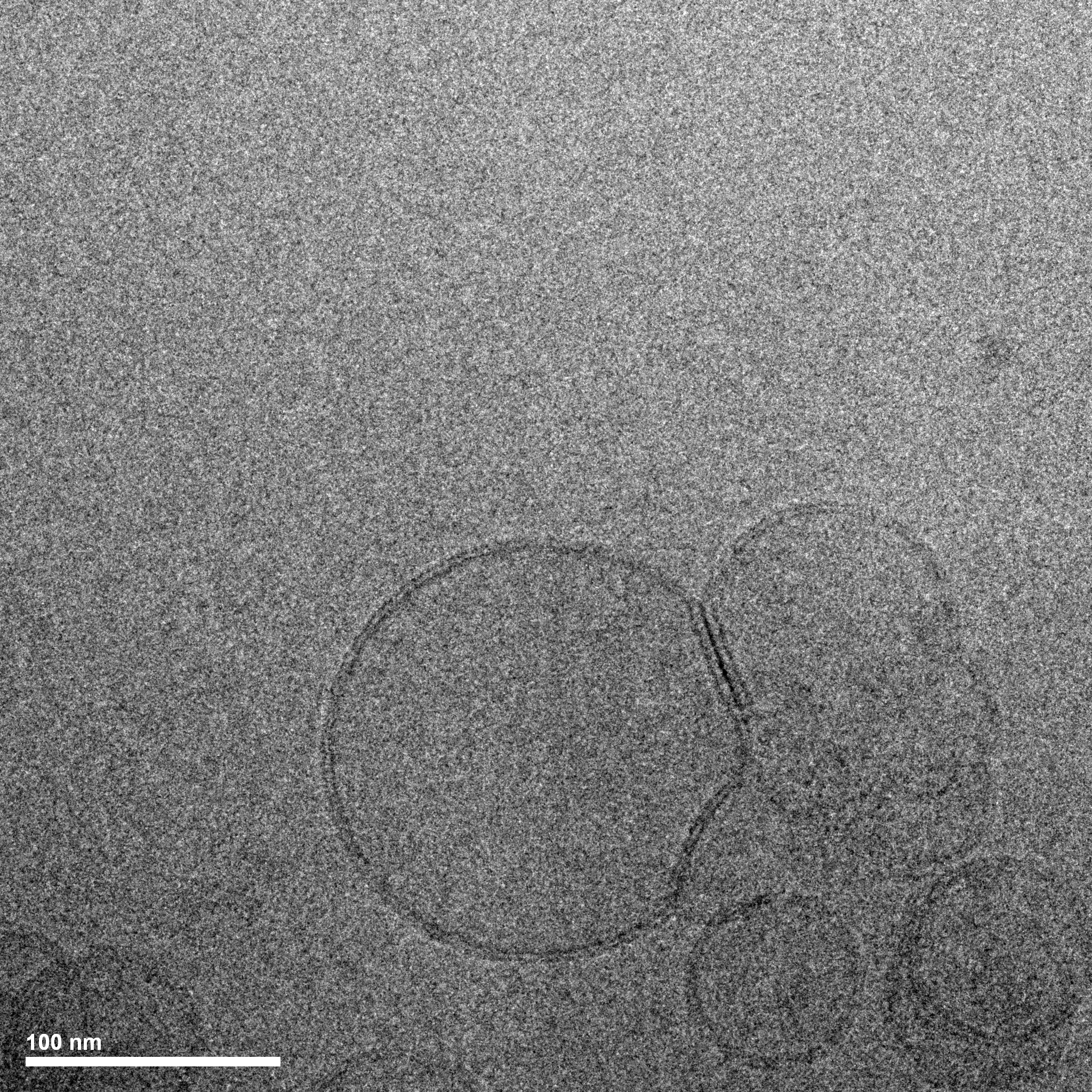

Supplement: Figure 2—source data 1. — This zip archive contains all cryo-EM images used for the quantitative analyses shown in Fig. 2. The folder named “No_Ca++” contains the images before Ca++ addition (individual files are named P3_1_**. tif or jpg), and folder named “With_Ca++” contains the images ∼35s after Ca++ addition (individual files are named P3_3_**.tif or jpg). Images were collected in low dose conditions at 200 kV acceleration voltage on a CM200 FEG electron microscope (FEI) with a 2k × 2k Gatan UltraScan 1000 camera, at 50,000× magnification and 1.5 mm underfocus. The full resolution data were exported as 16 bit “tif” files (2048 × 2048 pixels, scale 0.2 nm/pixel at specimen (the corresponding files have the extension “tif”). Note that these files cannot not be viewed with a standard picture viewer, but must be viewed with a program, such as “ImageJ”. To facilitate easier viewing, the original images were converted to smaller (1024×1024, 0.4 nm/pixel), contrast adjusted jpeg images (8 bits) for easy and immediate visualization with commonly used picture viewers (the corresponding files have the extension “jpg”). DOI: http://dx.doi.org/10.7554/eLife.00109.005 [file elife00109s001.zip › elife00109s001/With_Ca++/P3_3_35.tif]
